# Enhanced neoepitope-specific immunity following neoadjuvant PD-L1 and TGF-**β** blockade in HPV-unrelated head and neck cancer

**DOI:** 10.1172/JCI161400

**Published:** 2022-09-15

**Authors:** Jason M. Redman, Jay Friedman, Yvette Robbins, Cem Sievers, Xinping Yang, Wiem Lassoued, Andrew Sinkoe, Antonios Papanicolau-Sengos, Chyi-Chia Lee, Jennifer L. Marte, Evrim Turkbey, Wojtek Mydlarz, Arjun Joshi, Nyall R. London, Matthew Pierce, Rodney Taylor, Steven Hong, Andy Nguyen, Patrick Soon-Shiong, Jeffrey Schlom, James L. Gulley, Clint T. Allen

**Affiliations:** 1 Genitourinary Malignancy Branch and; 2 Laboratory of Tumor Immunology and Biology, National Cancer Institute (NCI), Center for Cancer Research, NIH, Bethesda, Maryland, USA.; 3 Section on Translational Tumor Immunology, National Institute on Deafness and Other Communication Disorders (NIDCD), NIH, Bethesda, Maryland, USA.; 4 Tumor Immune Microenvironment Laboratory, Genitourinary Malignancy Branch, NCI, and; 5 Laboratory of Pathology, Center for Cancer Research, NIH, Bethesda, Maryland, USA.; 6 Radiology and Imaging Sciences, NIH Clinical Center, Bethesda, Maryland, USA.; 7 Department of Otolaryngology–Head and Neck Surgery, Johns Hopkins School of Medicine, Baltimore, Maryland, USA.; 8 Division of Otolaryngology–Head and Neck Surgery, Department of Surgery, George Washington University, Washington, DC, USA.; 9 Department of Otolaryngology–Head and Neck Surgery, Georgetown University School of Medicine, Washington, DC, USA.; 10 Department of Otolaryngology–Head and Neck Surgery, University of Maryland School of Medicine, Baltimore, Maryland, USA.; 11 Department of Otolaryngology–Head and Neck Surgery, Walter Reed National Military Medical Center, Bethesda, Maryland, USA.; 12 ImmunityBio, Culver City, California, USA.

**Keywords:** Clinical Trials, Immunology, Immunotherapy

## Abstract

**BACKGROUND:**

Head and neck squamous cell carcinoma not associated with HPV (HPV-unrelated HNSCC) is associated with a high rate of recurrence and poor survival.

**METHODS:**

We conducted a clinical trial in 14 patients with newly diagnosed HPV-unrelated HNSCC to evaluate the safety and efficacy of neoadjuvant bintrafusp alfa, a bifunctional fusion protein that blocks programmed death ligand 1 (PD-L1) and neutralizes TGF-β.

**RESULTS:**

Bintrafusp alfa was well tolerated, and no treatment-associated surgical delays or complications occurred. Objective pathologic responses (PRs) were observed, and 12 of the 14 (86%) patients were alive and disease free at 1 year. Alterations in Treg infiltration and spatial distribution relative to proliferating CD8^+^ T cells indicated a reversal of Treg immunosuppression in the primary tumor. Detection of neoepitope-specific tumor T cell responses, but not virus-specific responses, correlated with the development of a PR. Detection of neoepitope-specific responses and PRs in tumors was not correlated with genomic features or tumor antigenicity but was associated with reduced pretreatment myeloid cell tumor infiltration. These results indicate that dual PD-L1 and TGF-β blockade can safely enhance tumor antigen–specific immunity and highlight the feasibility of multimechanism neoadjuvant immunotherapy for patients with HPV-unrelated HNSCC.

**CONCLUSION:**

Our studies provide insight into the ability of neoadjuvant immunotherapy to induce polyclonal neoadjuvant–specific T cell responses in tumors and suggest that features of the tumor microenvironment, such as myeloid cell infiltration, may be a major determinant of enhanced antitumor immunity following such treatment.

**TRIAL REGISTRATION:**

ClinicalTrials.gov NCT04247282.

**FUNDING:**

This work was funded by the Center for Cancer Research, the NCI, and the Intramural Research Program of the NIDCD, NIH. Bintrafusp alfa was provided by the health care business of Merck KGaA (Darmstadt, Germany), through a Cooperative Research and Development Agreement with the NCI. Additional funding was provided by ImmunityBio through a Cooperative Research and Development Agreement with the NIDCD.

## Introduction

Oncologic outcomes following standard-of-care treatment of newly diagnosed, advanced-stage head and neck squamous cell carcinoma not associated with HPV (HPV-unrelated HNSCC) are poor, with disease relapse occurring in approximately 50% of patients (1, 2). For patients who experience a relapse of disease, treatment with programmed cell death protein 1 (PD-1) pathway immune checkpoint blockade (ICB) alone or in combination with chemotherapy results in improved survival compared with chemotherapy alone (3). Preclinical mechanistic data suggest that neoadjuvant ICB may enhance tumor-specific immunity more effectively than adjuvant ICB through induction of polyclonal antigen–specific T cell responses in the presence of tumor antigen (4). Clinical studies of neoadjuvant ICB in resectable melanoma, non–small cell lung cancer, and glioblastoma demonstrated improved recurrence-free survival (RFS) following neoadjuvant ICB and revealed that pathologic responses (PRs) may correlate with durable treatment responses and survival (5–7). Initial clinical studies of neoadjuvant ICB in patients with newly diagnosed HPV-unrelated HNSCC indicated that PD-1, with or without cytotoxic T lymphocyte antigen 4 (CTLA-4) blockade, improves RFS (8–10), but tissue correlates and predictors of response have not been thoroughly studied.

TGF-β induces cell-cycle arrest in normal epithelial cells but promotes a malignant phenotype in the setting of transformed carcinoma cells (11). TGF-β also disrupts innate and adaptive immunity, suppressing cytotoxic T cell function and promoting Treg development (12). For these reasons, therapeutic neutralization of TGF-β may inhibit tumor growth and enhance antitumor immunity. Bintrafusp alfa, a bifunctional fusion protein composed of the extracellular domain of the human TGF-β receptor II (TGF-βRII or TGF-β “trap”) fused via a flexible linker to the C-terminus of each heavy chain of an IgG1 antibody blocking programmed death ligand 1 (anti–PD-L1) (13), has demonstrated significant activity in preclinical studies and is in various stages of clinical development for several cancers (14).

Here, we report the feasibility, safety, and efficacy of neoadjuvant PD-L1 and TGF-β blockade with bintrafusp alfa in patients with newly diagnosed HPV-unrelated HNSCC. We used multispectral immunofluorescence (MIF), genomic and transcriptomic sequencing, and tumor antigen–specific T cell response assays to explore correlations with PRs and to identify possible biomarkers of tumor regression following neoadjuvant treatment.

## Results

### Patient characteristics and safety.

**Figure 1:**
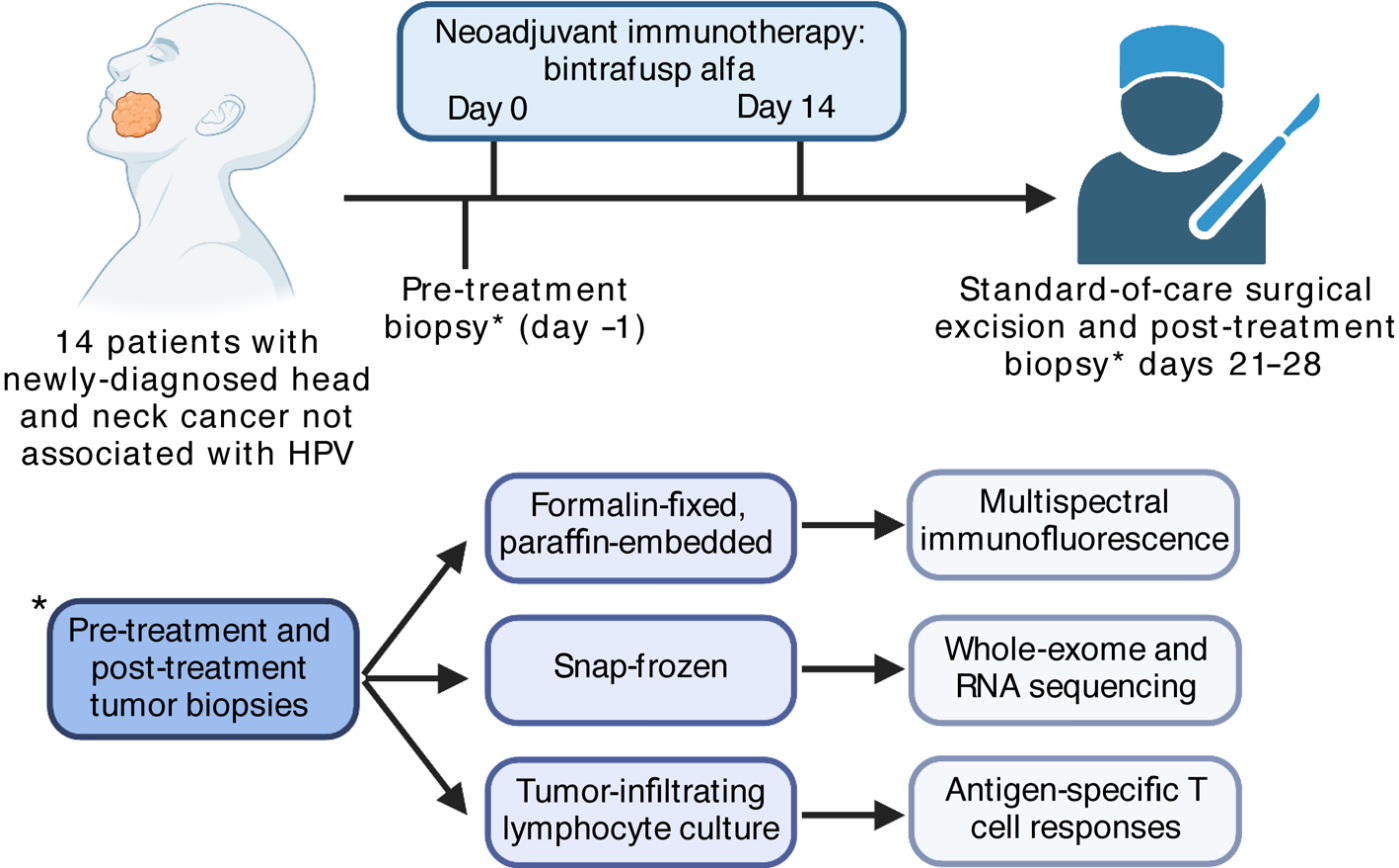
Clinical and correlative study design. Schema illustrates the neoadjuvant immunotherapy clinical trial design for the 14 study patients as well as the correlative studies performed on pre- and post-treatment tumor biopsies.

Fourteen patients were treated from June 2020 to February 2021 (Figure 1). The baseline characteristics of all 14 patients are listed in Table 1. Eleven (79%) patients had clinically or radiographically node-positive disease, and 10 (71%) had stage IV disease. The median follow-up was 14.7 months (range, 10–19 months). Twelve (86%) patients received 2 doses of bintrafusp alfa. One patient did not receive a second dose after developing a nocturnal grade 2 tumor hemorrhage attributed to inadvertent mastication of the primary tumor. Another patient did not receive a second dose because of transient grade 1 bilirubin elevation of unclear etiology. Both patients proceeded to surgery without complications. The median number of days from first neoadjuvant immunotherapy treatment to surgery was 22. None of the patients experienced a treatment-related delay in the planned surgery. Free tissue transfer reconstruction was required for 71% of patients. There was no unexpected bleeding or delayed wound healing during or following surgery.

Seventy-six percent of participants experienced an adverse event that was at least possibly related to the study treatment. Treatment-related adverse events (TRAEs) are summarized in Table 2. There were no TRAEs above grade 3. One participant developed grade 2 hyperthyroidism that improved with medical management and later required oral thyroid replacement once hypothyroidism developed. One participant developed grade 3 vasculitis that improved with corticosteroids.

### Pathologic, radiographic, and clinical responses.

**Figure 2:**
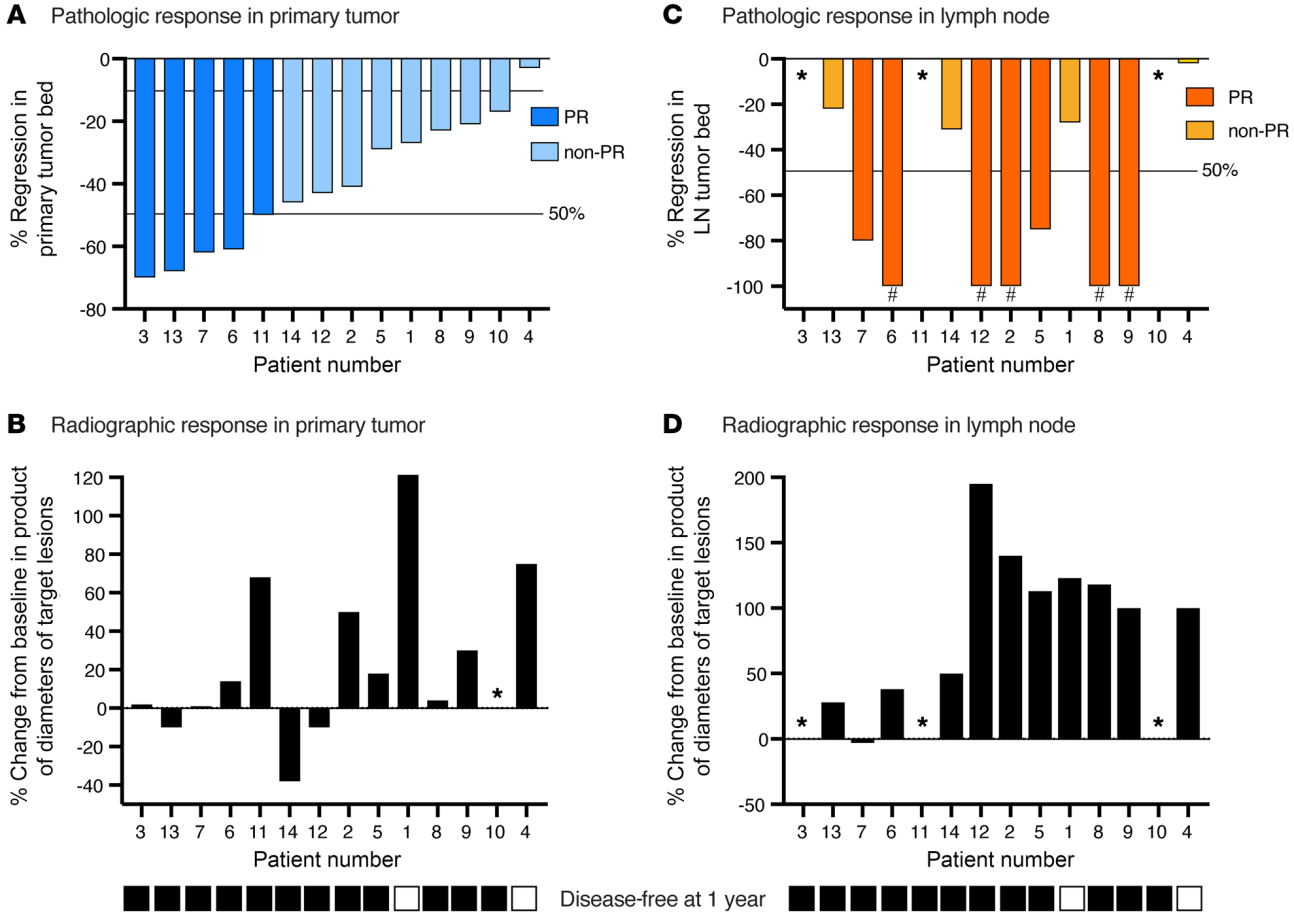
Primary tumor and LN responses. (**A**) Waterfall plot shows primary tumor PRs (*n* = 14), ranked from left to right by decreasing percentage of tumor regression within the tumor bed. A PR was defined as a pTR of 50% or greater; a non-PR was defined as a pTR of less than 50%. (**B**) Waterfall plot shows primary tumor radiographic responses (*n* = 14), calculated as the percentage of change in the product of the longest primary tumor lengths and widths, ranked from left to right in the same order as in **A**. The asterisks indicate that the primary tumor was not visible on the CT scan. (**C**) Waterfall plot shows LN PRs (*n* = 14), ranked from left to right by decreasing percentage of primary tumor regression within the tumor bed. A PR was defined as a pTR of 50% or greater. The pound signs indicate patients who had suspicious LNs on pretreatment workup but were found to be pathologically N0 and considered to have a possible LN CR. (**D**) Waterfall plot shows LN radiographic responses (*n* = 14), calculated as the percentage of change in the product of the largest bidirectional diameter of a target suspicious LN, ranked from left to right in the same order as in **A**. In patients with LNs positive for carcinoma, the volume of all positive nodes was considered. For patients with suspicious nodes negative for carcinoma pathologically, only the radiographically suspicious LN volume was considered. The asterisks indicate that no clinically suspicious or pathologically positive LNs were found. The bottom black (yes) and white (no) boxes indicate patients with a disease-free status 1 year after completing the study.

All 14 patients were scored for pathologic tumor response (pTR) in post-treatment surgical specimens. Primary tumors displayed pTRs ranging from 3% to 70% (Figure 2A; examples of primary tumor histology and pTR scoring are shown in Supplemental Figure 1, A–C; supplemental material available online with this article; https://doi.org/10.1172/JCI161400DS1). Using standardized immune-related PR definitions (15, 16), no primary tumor had a complete (100% pTR) or major (pTR ≥90%) response, and 5 tumors showed a PR (pTR ≥50%), yielding an overall primary tumor PR rate of 36%. The primary tumor volume on CT imaging increased in 10 (71%) patients and decreased in 3 (21%) patients (Figure 2B). We found no obvious correlation between the magnitude of the pTR and changes in the radiographic volume of the primary tumors following treatment.

Five patients with clinical or radiographic evidence of nodal disease before treatment (patients 2, 6, 8, 9, and 12) harbored no viable tumor upon post-treatment pathologic analysis of their lymph nodes (LNs), suggesting that a pathologic complete response (pCR) in the LNs may have occurred (Figure 2C and Supplemental Figure 2). These CRs are only speculative, because although the specificity of PET avidity for nodal metastasis is very high (17), the presence of carcinoma in these LNs was not proven before treatment with a biopsy. Two additional patients (patients 5 and 7) with pathologically proven LN disease had a 50% or higher pTR in the LNs. Ten of 11 (91%) patients with clinically suspicious or pathologically proven nodal disease demonstrated increased volume on CT imaging after treatment compared with before treatment (Figure 2D), including 5 patients with suspicious LNs at diagnosis that were pathologically without nodal disease (N0). We found no correlation between the LN pTR and the change in CT volume. Defining discordant responses as primary tumor and confirmed or possible LN responses that differed by more than 20% for an individual patient, we found that 8 (73%) patients had discordant responses (Supplemental Figure 3). Considering all evaluable surgical tissues, 6 of 14 (43%) patients definitively displayed at least a partial PR (pPR) in the primary tumor or LN. If patients whose pretreatment suspicious LNs were found to be pathologically N0 had true CRs in the neck, 10 of 14 (71%) patients had at least a pPR in the primary tumor or LN.

Two patients had locoregional disease recurrence within 1 year of completing the study (disease in patient 1 recurred at 7 months; disease in patient 4 recurred at 9 months), resulting in a 1-year RFS rate of 86%. Both patients died of complications due to locoregional recurrence, and neither patient had evidence of distant metastatic disease at the time of death.

### Assessment of primary tumor TGF-β pathway signaling.

**Figure 3:**
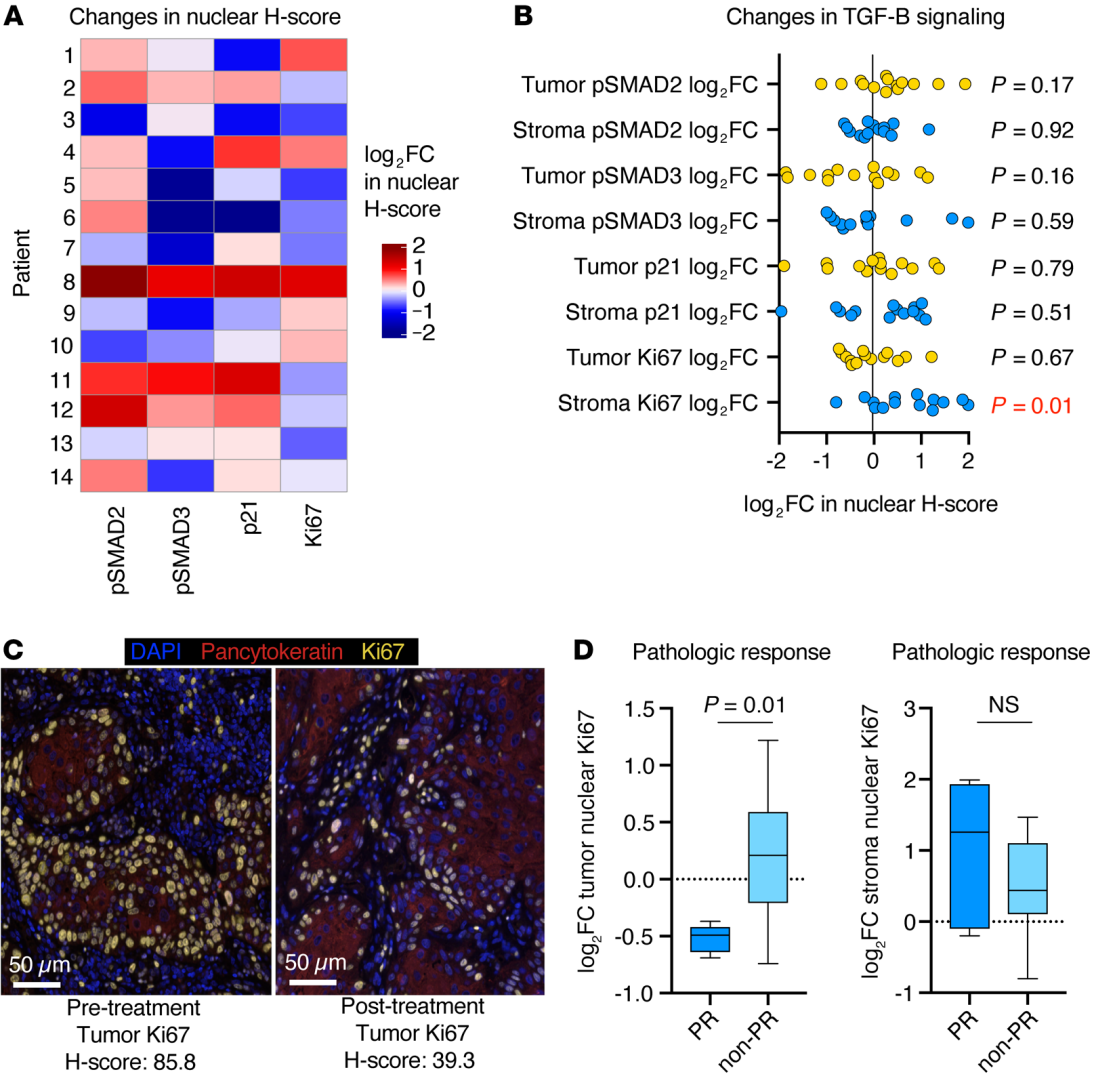
MIF analysis of tumor TGF-β pathway activation. (**A**) Heatmap shows patterns of log_2_ FC in nuclear H-scores of TGF-β pathway signaling proteins after treatment compared with before treatment for each patient (*n* = 14). (**B**) Dot plot shows the log_2_ FC in nuclear H-scores of TGF-β pathway signaling proteins in the stroma or tumor parenchyma after treatment compared with before treatment (*n* = 14). Most stromal cells were immune (mean, 79%; range, 59%–93%), based on size and morphology. Significance was determined with a 1-sample *t* test; the *P* value in red is significant. (**C**) Representative photomicrographs of pre- and post-treatment tumor Ki67 expression for patient 13, measured by immunofluorescence. Scale bars: 50 μm. (**D**) Box-and-whisker plots show the log_2_ FC in the tumor or stromal nuclear Ki67 H-score after treatment compared with before treatment in patients who did (*n* = 5) or did not (*n* = 9) have a PR. Significance was determined with a 2-tailed Mann-Whitney *U* test.

Pre- and post-treatment primary tumor biopsies were available for correlative analysis for all 14 patients. We performed MIF analysis to evaluate changes in TGF-β pathway signaling (Supplemental Figure 4, A and B). In nonmalignant epithelial cells with intact TGF-β pathway signaling, TGF-β is antiproliferative, and neutralization is expected to result in decreased tumor cell phosphorylated SMAD2 (p-SMAD2), p-SMAD3, and p21 and increased Ki67 (a marker of proliferation) (18). We observed this pattern of changes in only 2 (14%) patients (Figure 3A, patients 9 and 10). Instead, tumors displayed inconsistent patterns of changes, with decreased expression of tumor cell p-SMAD2, p-SMAD3, and p21 in 4 (29%), 7 (50%), and 5 (37%) samples, respectively, and an increase in tumor cell Ki67 expression in 5 (37%) samples. Conversely, stromal nuclei, consisting primarily of immune cells, had increased Ki67 expression in 11 (79%) samples (Figure 3B). Patients who developed a primary tumor PR after treatment showed significantly reduced tumor cell Ki67 expression after treatment compared with patients who did not have a PR in the primary tumor (Figure 3, C and D).

We performed whole-exome sequencing (WES) to determine the underlying mutational profile of primary tumors and whether pretreatment tumors harbored mutations in core TGF-β signaling genes (19). The mutational profiles of these tumors were generally consistent with profiles of carcinogen-associated head and neck cancers reported previously (Supplemental Figure 5) (20). One or more mutations in core TGF-β signaling genes (19) were observed in 13 (93%) tumors, but only 1 mutation in 1 patient was in silico–predicted to be functionally deleterious (polyphen analysis) (Supplemental Figure 6A). We found no correlation between the number of mutated core TGF-β signaling genes and the PR (Supplemental Figure 6B). Changes in TGF-β target gene expression were measured with bulk RNA-Seq (19). No clear patterns of transcriptional changes were evident after treatment with bintrafusp alfa, possibly because of the difficulty of studying individual signaling pathways in bulk RNA-Seq data (Supplemental Figure 6C).

Overall, these results suggest that TGF-β signaling was uncoupled from direct control of primary tumor cell proliferation through mechanisms that were independent of canonical TGF-β pathway genomic alterations. These data also indicate that the most common cumulative primary tumor effect of dual PD-L1 and TGF-β blockade with bintrafusp alfa treatment was a reduction of tumor cell proliferation and increased proliferation of cells in the stromal compartment, possibly indicating immune activation.

### Measurement of immune cell density and spatial localization.

**Figure 4:**
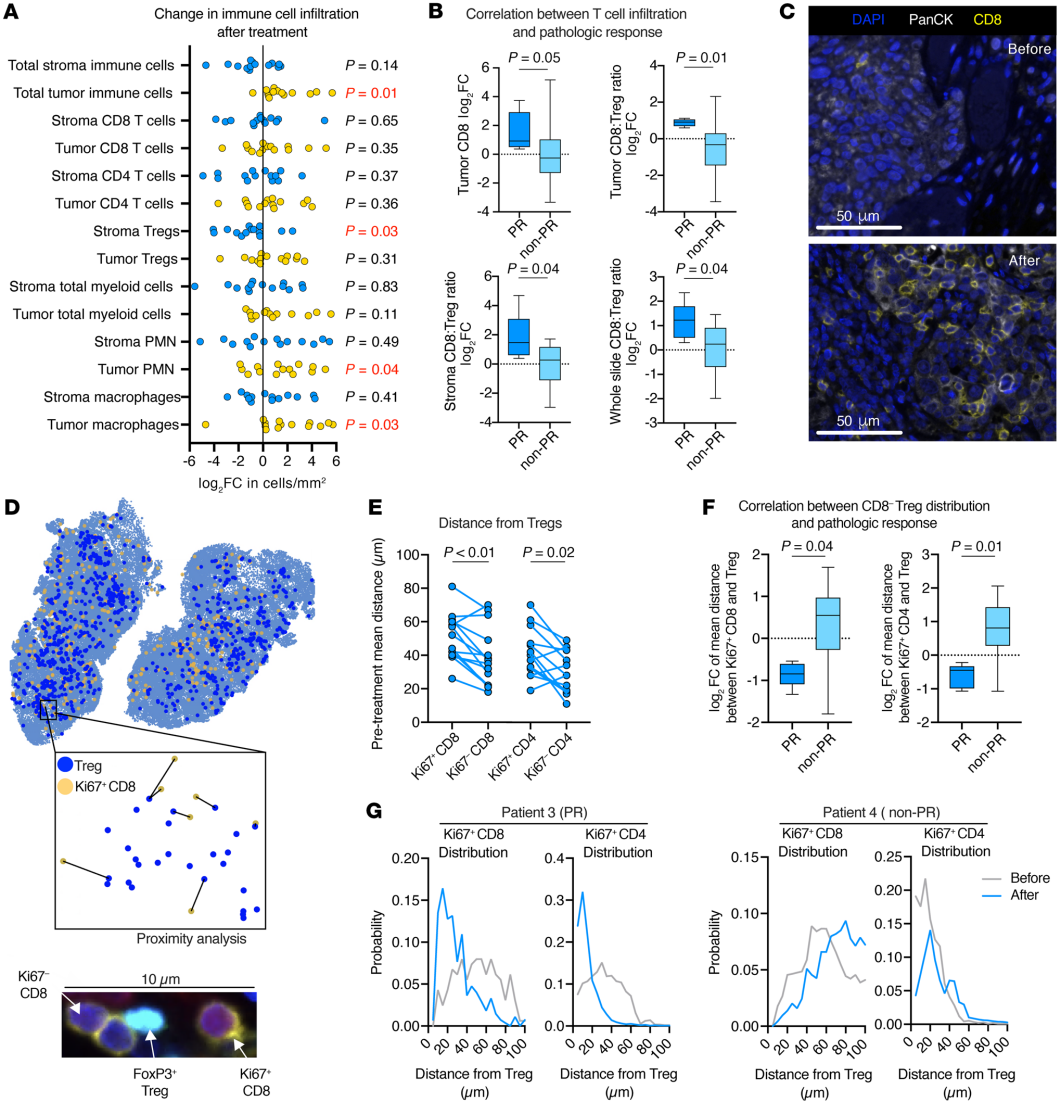
MIF analysis of tumor T cell infiltration and spatial distribution. (**A**) Dot plot shows the log_2_ FC in immune cell density (cells/mm^2^) in the stroma or tumor parenchyma after treatment compared with before treatment (*n* = 14). Significance was determined with a 1-sample *t* test; *P* values in red are significant. (**B**) Box-and-whisker plots show the log_2_ FC in tumor CD8^+^ density as well as the tumor, stroma, and whole-slide CD8/Treg ratio after treatment compared with before treatment in patients who did (*n* = 5) or did not (*n* = 9) show a PR. Significance was determined with a 2-tailed Mann-Whitney *U* test. (**C**) Representative high-magnification photomicrographs of CD8^+^ staining for patient 3, who had a large increase in tumor CD8^+^ T cell infiltration after treatment compared with before treatment. Scale bars: 50 μm. (**D**) Representative image of a HALO spatial plot used to perform proximity analysis (inset) of FoxP3^–^ T cells and FoxP3^+^ Tregs. A representative photomicrograph of a Treg directly interacting with a Ki67^–^CD8^+^ T cell is shown below. (**E**) Assessment of FoxP3^–^CD8^+^ or CD4^+^ T cells within a 100 μM radius of all FoxP3^+^CD4^+^ Tregs, with dot plot showing the mean distance between Ki67^+^ or Ki67^–^ CD8^+^ or CD4^+^ T cells and Tregs in pretreatment tumors as determined by MIF (*n* = 14). Lines connect pre- and post-treatment measurements from individual tumors. Significance was determined with a Wilcoxon signed-rank test. (**F**) Assessment of all FoxP3^–^CD8^+^ or CD4+ T cells within a 100 μM radius of all FoxP3^+^CD4^+^ Tregs. Box-and-whisker plots show the log_2_ FC in the mean distance between Ki67^+^CD8^+^ or CD4^+^ T cells and Tregs after treatment compared with before treatment in patients who did (*n* = 5) or did not (*n* = 9) have a PR. Significance determined with a 2-tailed Mann-Whitney *U* test. (**G**) Distribution plots show the probability that a Ki67^+^CD8^+^ or CD4^+^ T cell will be at a given distance from a Treg in pretreatment (gray line) and post-treatment (blue line) tumors. Patient 3 is a representative example of a patient who developed a PR; patient 4 did not develop a PR.

To study immune responses in primary tumors, we first used MIF to measure changes in the infiltration and localization of T cells and myeloid cells (Supplemental Figure 4C). The immune cell phenotypes are listed in Supplemental Table 1. We observed an increase in total immune cell infiltration into the tumor parenchyma, indicating a more inflamed tumor phenotype after treatment (Figure 4A). Stromal immune cell density was inconsistently increased or decreased, except for stromal Tregs, which were reduced in 12 (86%) tumors, and myeloid cell infiltration into the tumor parenchyma, which was frequently increased after treatment compared with before treatment. We observed no correlation between pretreatment tumor or stromal CD8^+^ or CD4^+^ T cell or Treg density and a PR. Pretreatment tumor cell PD-L1 expression also did not predict a PR (Supplemental Figure 7). We assessed the changes in T cell density following treatment and found that increased tumor parenchyma infiltration of CD8^+^ T cells and an increased CD8/Treg ratio within the tumor parenchyma or stroma after treatment compared with before treatment correlated with a PR (Figure 4B, representative photomicrographs shown in Figure 4C).

Although we did not observe consistent changes in the density of proliferating CD8^+^ or CD4^+^ T cells after treatment (Supplemental Figure 8A), analysis of digitized MIF images allowed for the study of spatial relationships between proliferating or nonproliferating FoxP3^–^CD8^+^ or FoxP3^–^CD4^+^ T cells and FoxP3^+^CD4^+^ Tregs (Figure 4D). Before treatment, replicating Ki67^+^CD8^+^ or Ki67^+^CD4^+^ T cells were, on average, a greater distance from Tregs than were nonproliferating Ki67^–^CD8^+^ or Ki67^–^CD4^+^ T cells (Figure 4E). After treatment, the mean distance between proliferating CD8^+^ or CD4^+^ T cells and Tregs was reduced in patients who had a PR but was not reduced in patients who did not (Figure 4F). We observed no change in the mean distance between nonproliferating T cells and Tregs (Supplemental Figure 8B). Measurement of the probability that a proliferating CD8^+^ or CD4^+^ T cell is a given distance from a Treg revealed a shift to the left of the distribution curve in patients who experienced a PR (e.g., patient 3); no shift was observed in those who did not have a PR (e.g., patient 4) (Figure 4G). We did not find this to be the case when considering the distribution curves of nonproliferating T cells around Tregs (Supplemental Figure 8C). These findings offer correlative evidence that Tregs were immunosuppressive in pretreatment tumors, and shifts in distribution curves after treatment suggest that Treg immunosuppression was inhibited in patients who experience a PR.

**Figure 5:**
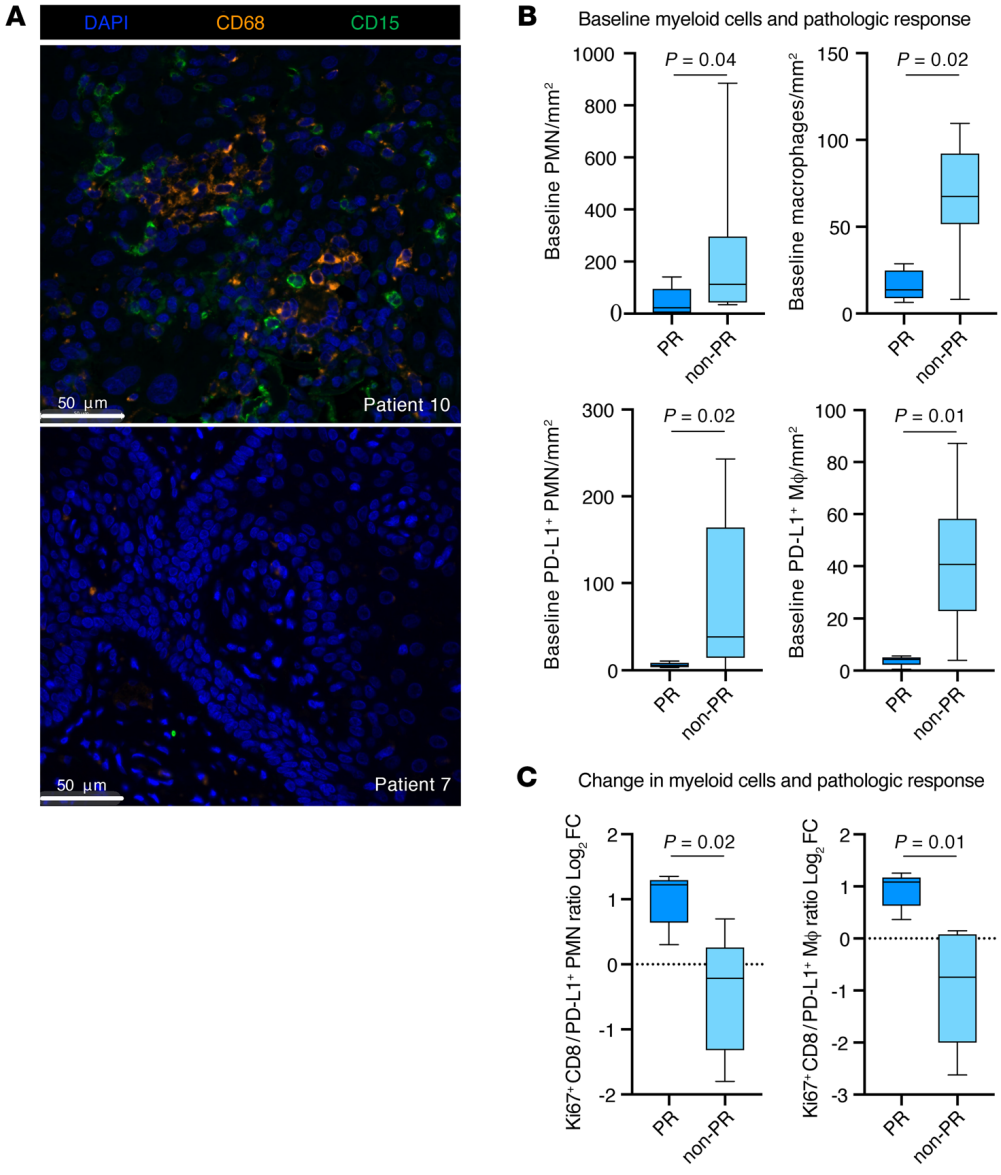
MIF analysis of tumor myeloid cell infiltration. (**A**) Representative photomicrographs of pretreatment tumor myeloid cell infiltration measured by immunofluorescence. Patient 10 showed high myeloid infiltration, and patient 7 showed low infiltration. Scale bars: 50 μm. (**B**) Box-and-whisker plots show quantification of pretreatment density (cells/mm^2^) of PD-L1^+^ or PD-L1^–^ PMNs or macrophages in patients who did (*n* = 5) or did not (*n* = 9) have a PR. Significance determined with a 2-tailed Mann-Whitney *U* test. (**C**) Box- and-whisker plots show the log_2_ FC in the stromal Ki67^+^ to PD-L1^+^ PMN or macrophage ratio after treatment compared with before treatment in patients who did (*n* = 5) or did not (*n* = 9) have a PR. Significance was determined with a 2-tailed Mann-Whitney *U* test. MΦ, macrophage.

We also performed myeloid MIF to study the density of neutrophilic cells (polymorphonuclear cells [PMNs]) and macrophages (Supplemental Figure 4D). Although the pretreatment density of T cells was not correlated with the development of a PR after treatment, a reduction in the pretreatment density of PD-L1^+^ or PD-L1^–^ PMNs or macrophages in the stroma predicted a PR (Figure 5, A and B). We assessed changes with treatment and found that an increased Ki67^+^CD8^+^/PD-L1^+^ PMN or Ki67^+^CD8^+^/PD-L1^+^ macrophage ratio after treatment compared with before treatment correlated with a PR (Figure 5C).

Using bulk RNA-Seq data, we assessed the expression of a selected set of immune-related genes found to be predictive of a response to neoadjuvant pembrolizumab (8) for correlation with a PR following treatment with bintrafusp alfa. No clear pattern between expression of this gene set in pretreatment samples and the development of a PR was observed (Supplemental Figure 9A). Most tumors (11 of 14, 79%) displayed an increase in expression of these immune-related genes after treatment with bintrafusp alfa, but there was no clear correlation between the magnitude of the increase after treatment and the development of a PR (Supplemental Figure 9B). Immune deconvolution (FarDeep) was performed on bulk RNA-Seq data to estimate immune cell infiltration (21). FarDeep analysis estimated an increase in immune infiltration in tumors that displayed a greater PR (Supplemental Figure 9C). A strong positive correlation existed between the FarDeep estimation of changes in CD8^+^ infiltration and the direct measurement of changes in CD8^+^ cell density by MIF (Supplemental Figure 9D). We did not observe a positive correlation for CD4^+^ infiltration.

### Determination of tumor antigen–specific T cell responses.

**Figure 6:**
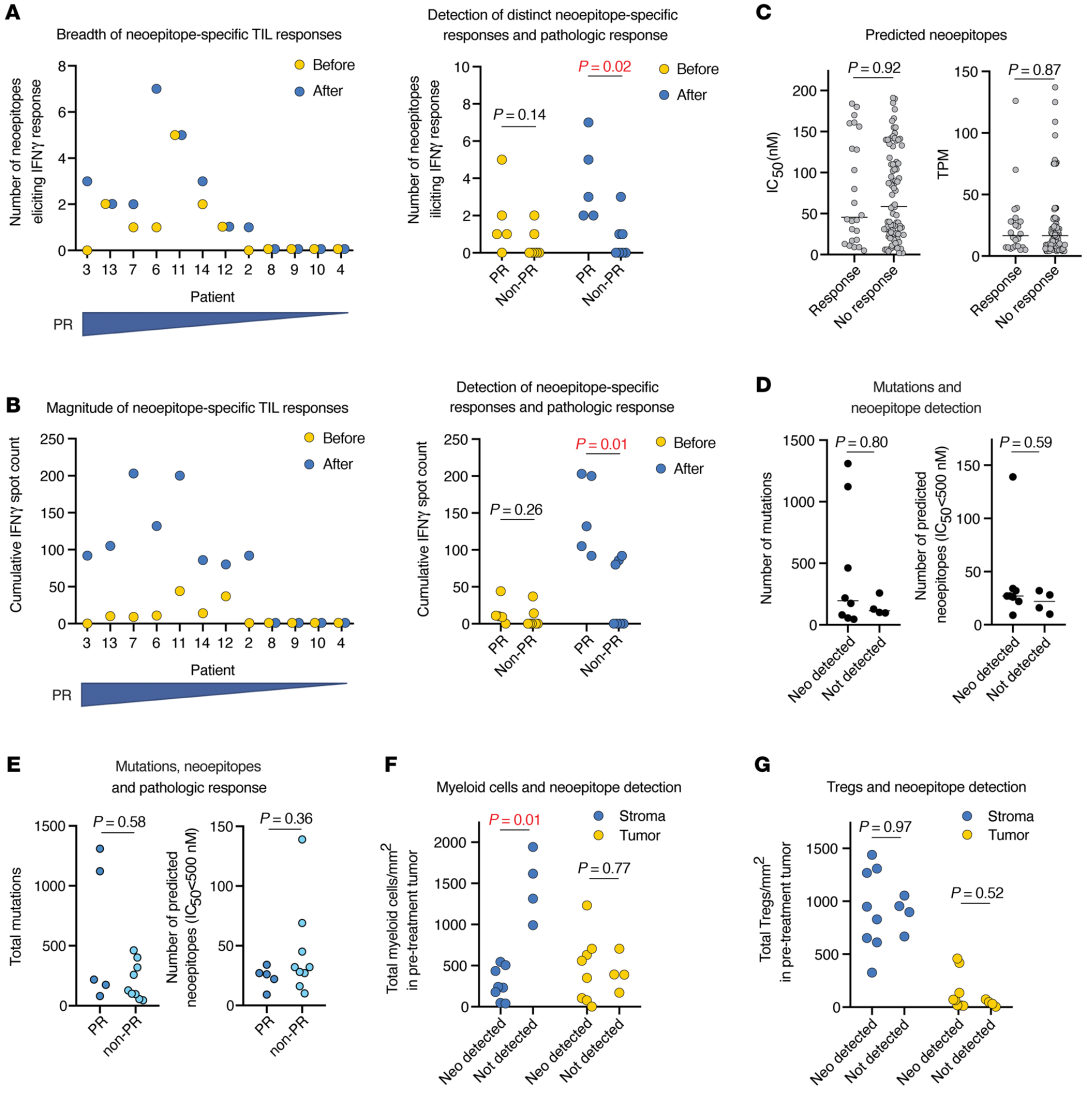
Neoepitope-specific TIL responses. (**A**) Dot plot on the left shows the number of distinct neoepitopes eliciting positive IFN-γ responses in an ELISpot analysis of pre-treatment and post-treatment TILs for each patient (*n* = 12), ranked from left to right by decreasing percentage of tumor regression within the tumor bed. Dot plot on the right shows the same in patients who did (*n* = 5) or did not (*n* = 7) have a PR. Significance was determined with a 2-tailed Mann-Whitney *U* test. (**B**) Dot plot on the left shows the number of neoepitope-specific cumulative IFN-γ spots in an ELISpot analysis of pre- and post-treatment TILs for each patient (*n* = 12), ranked from left to right by decreasing percentage of tumor regression within the tumor bed. Dot plot on the right shows the same in patients who did (*n* = 5) or did not (*n* = 7) have a PR. Significance was determined with a 2-tailed Mann-Whitney *U* test. (**C**) Dot plots show the in silico–predicted IC_50_ and TPM counts for putative neoepitopes that elicited (*n* = 24) or did not elicit (*n* = 84) IFN-γ responses in TILs. Horizontal bar indicates the median. Significance was determined with a 2-tailed Mann-Whitney *U* test. (**D**) Dot plots show the total number of mutations and total number of predicted neoepitopes (IC_50_ <500 nM) in tumors that did (*n* = 8) or did not (*n* = 4) display detectable neoepitope-specific T cell responses in TILs. Significance was determined with a 2-tailed Mann-Whitney *U* test. (**E**) Dot plots show the total number of mutations and total number of predicted neoepitopes (IC_50_ <500 nM) in patients who did (*n* = 5) or did not (*n* = 97) have a PR. Significance was determined with a 2-tailed Mann-Whitney *U* test. (**F**) Dot plot shows pretreatment tumor quantification of total stromal or tumor myeloid cells in patients whose TILs did (*n* = 8) or did not (*n* = 4) display detectable neoepitope-specific T cell responses. Significance was determined with a 2-tailed Mann-Whitney *U* test. (**G**) Dot plot shows pretreatment tumor quantification of stromal cells or tumor Tregs from patients whose TILs did (*n* = 8) or did not (*n* = 4) display detectable neoepitope-specific T cell responses. Significance was determined with a 2-tailed Mann-Whitney *U* test.

We further studied changes in immune activation by determining whether treatment with bintrafusp alfa induced new or expanded existing tumor antigen–specific T cell responses and if such changes correlated with a PR. Tumor-infiltrating lymphocytes (TILs) cultured from pre- and post-treatment tumors from 12 patients were assessed for IFN-γ production upon coculturing with autologous antigen-presenting cells loaded with in silico–predicted candidate patient-specific, mutation-derived neoepitopes or common viral antigens. The high-affinity candidate neoepitopes selected from each patient for synthesis (see Methods for criteria) are listed in Supplemental Table 2. Among all patients, we detected neoepitope-specific T cell responses among 24 of 108 (22%) predicted neoepitopes. All neoepitope-specific T cell responses were detectable (spot count >0) in post-treatment TILs. Of 24 neoepitope-specific responses in post-treatment TILs, 12 (50%) were detected (spot count >0) in patient-matched pretreatment TILs. Nineteen of 24 (80%) neoepitope-specific TIL responses were increased (difference >5 IFN-γ spots) after treatment compared with before treatment. For individual patients, a greater number of post-treatment neoepitopes for which responses were measured and a greater cumulative neoepitope-specific magnitude of response (IFN-γ spot count) each correlated with a primary tumor PR (Figure 6, A and B). T cell responses to common viral antigens (CEF) were detected in 6 of 12 (50%) patients. We found no correlation between a virus-specific T cell response and a primary tumor PR (Supplemental Figure 10). From these data, we conclude that neoadjuvant PD-L1 and TGF-β blockade increased detectable neoepitope-specific immunity in 8 of 12 (67%) patients and that detection of neoepitope-specific responses, but not responses to viral antigens, correlated with the development of a PR.

Neoepitope-specific responses were not detected in 4 of 12 (33%) patients. No correlation existed between the in silico–predicted neoepitope IC_50_ or the neoepitope expression level and the detection of a neoepitope-specific T cell response (Figure 6C). The mutational burden and number of predicted neoepitopes with an IC_50_ of less than 500 nM did not significantly differ in patients with or without detected neoepitope-specific responses (Figure 6D) or a PR (Figure 6E). These data indicate that antigenicity was not a major determinant of the ability to detect neoepitope-specific responses in this data set. Considering the pretreatment tumor microenvironment, reduced stromal myeloid cell density predicted the detection of 1 or more neoepitope-specific responses after neoadjuvant treatment (Figure 6F). The density of Treg in the tumor or stroma did not predict a response (Figure 6G). Thus, pretreatment tumor myeloid cell infiltration may represent a biomarker that predicts the ability to detect neoepitope-specific T cell responses and the associated development of a PR after neoadjuvant treatment.

## Discussion

Here, we report what to our knowledge is the first study to describe the clinical results and tissue correlative analyses of neoadjuvant PD-L1 and TGF-β blockade in patients with newly diagnosed HPV-unrelated HNSCC. We found that 1 or 2 neoadjuvant doses of bintrafusp alfa were well tolerated in this patient population. Treatment-related adverse events that delayed standard-of-care surgery were not observed, consistent with prior studies of neoadjuvant PD-1 blockade with or without CTLA-4 blockade (8–10, 22). Although our study had a small sample size, the 1-year RFS of 86% with neoadjuvant bintrafusp alfa is similar to that observed in similar patient populations treated with neoadjuvant nivolumab with or without ipilimumab (9) or a neoadjuvant plus the adjuvant pembrolizumab (8). Clinical development of bintrafusp alfa in the relapse setting has focused primarily on patients with HPV-associated cancer (23, 24). Here, we provide evidence of activity in cancer not associated with HPV in the up-front, neoadjuvant treatment setting, consistent with preclinical studies demonstrating efficacy in multiple cancer types (13, 14). Improved RFS is the goal of neoadjuvant treatment. Longer-term follow-up and larger studies are needed to determine whether the addition of TGF-β neutralization to neoadjuvant PD-(L)1 blockade improves RFS to a greater degree than PD-(L)1 blockade alone.

Neoadjuvant trial designs allow for extensive analyses of pre- and post-treatment tumor samples. Our studies validate the ability of neoadjuvant bintrafusp alfa to enhance polyclonal neoepitope-specific T cell responses in tumors and provide correlative evidence for a reduction in tumor Treg density and immunosuppressive function. We were unable to conclude whether the enhanced T cell responses resulted directly from activation of cytotoxic T cells or indirectly through inhibition of Tregs. Both are possibilities, given the known biologic roles of PD-(L)1 immune checkpoint signaling and TGF-β (12, 25).

The ability to detect neoepitope-specific T cell responses and the development of a PR following neoadjuvant treatment did not correlate with mutational burden or the expression or IC_50_ of predicted neoepitopes. This may be due to the relatively small sample size in our study. Yet, our finding that pretreatment myeloid cell density predicted both the development of a PR and the ability to detect neoepitope-specific T cell responses in tumors suggests that characteristics of the tumor microenvironment may also be a significant determinant of neoepitope-specific T cell function and PR in addition to genomic features such as mutational burden or antigenicity. This also indicates that the quantitative measurement of tumor myeloid infiltration may serve as a biomarker of response for neoadjuvant ICB. Tumor-infiltrating myeloid cells have been experimentally proven to be immunosuppressive in HNSCC (26–28). If validated in larger clinical data sets, this finding suggests that concurrent neoadjuvant inhibition of myeloid cell tumor trafficking or function may be a rational strategy to improve PRs and clinical outcomes with neoadjuvant immunotherapy.

T cells targeting neoepitopes in the tumor microenvironment are highly exhausted (29, 30). We believe our finding that only half of the validated neoepitope-specific T cell responses were detectable in pretreatment TILs is significant because it indicates that neoepitope-specific T cell profiling may be incomplete if the T cells being studied are from the microenvironment of untreated tumors (29, 31). Although challenging because of very low clonotype frequencies, detection of tumor antigen specificity in peripheral blood may yield a more comprehensive list of patient-specific neoepitopes if TILs cultured after ICB are unavailable for study (6, 32).

TGF-β is a pleiotropic cytokine with different effects on epithelial, stromal, and immune cells (33). Our results indicate that TGF-β neutralization reduced tumor cell proliferation in most patients, a finding that is the opposite of that observed following TGF-β neutralization in normal squamous mucosa and papillomas (18). Possibly related to tumor cell responses to effector molecules from T cells (34), evidence of reduced tumor cell proliferation was similarly observed following neoadjuvant ICB in patients with glioblastoma (7). Mutations predicted to alter the function of key TGF-β pathway components were infrequent, suggesting that disrupted antiproliferative TGF-β signaling may be driven by an epigenetic mechanism in these tumors. Radiographic changes alone demonstrated that some tumors and LNs had enlarged during this short treatment window, but pathologic analysis revealed a regression of viable tumor. The discordance between radiographic and pathologic assessments may be due to tumor inflammation (6, 9). Although the contribution of TGF-β neutralization to the observed tumor antigen–specific immune activation in our study is unclear, our data indicate that the net effect of TGF-β neutralization with PD-L1 blockade in newly diagnosed HPV-unrelated HNSCC is induction of antitumor immunity. Because of the variable biology among malignancies, the effect of bintrafusp alfa treatment on diverse tumor types in the newly diagnosed or relapsed setting may need to be studied individually.

The presence of detectable tumor antigen–specific T cells in the primary tumor correlated with a PR in the primary tumor but not in the LNs. Considering possible pCRs in LNs and true responses, most patients with disease in the LNs developed a stronger PR in the LNs compared with the PR observed in the primary tumor. If tumor antigen–specific T cell responses in the tumor reflect systemic antitumor immunity, a more immune-permissive microenvironment within LNs may be an explanation for these observed discordant PRs (35). Testing of this hypothesis would require similar antigen-specific response studies to be performed on TILs from primary tumors and LNs cultured separately for each patient. This should be explored in future neoadjuvant immunotherapy studies with sampling of LNs before and after treatment to allow both confirmation of the presence of carcinoma within suspicious nodes as well as the study of antigen-specific T cell responses.

Tumor antigen–specific T cells within the tumor microenvironment often express tissue retention markers such as CD103 and display evidence of activation and exhaustion with expression of PD-1, Tim3, Lag3, and others (29, 30). The isolation and study of TILs before and after neoadjuvant immunotherapy treatment with technologies such as single-cell RNA-Seq and T cell receptor–sequencing (TCR-Seq) could allow tracking of changes in individual T cell clonotypes. Such data could provide further insight into the baseline characteristics of tumor antigen–specific T cell clones within tumors that expand and activate with neoadjuvant immunotherapy, with some of these features possibly representing additional biomarkers of response.

Limitations of our study exist. The small sample size and 1-year follow-up make it difficult to draw conclusions about whether the rates of a PR or clinical benefit with PD-L1 and TGF-β blockade are superior to those observed with PD-(L)1 blockade alone. Validation of responses to PD-L1 and TGF-β blockade in additional cancer types would support further clinical development in the neoadjuvant realm. Also, our single-arm design did not allow us to determine whether a similar enhancement of neoepitope-specific responses in TIL occurs with PD-(L)1 blockade alone or whether TGF-β neutralization is required. Whether neoadjuvant PD-(L)1 blockade without TGF-β neutralization enhances neoepitope-specific tumor T cells should be studied. Our small sample size and low recurrence rate make it difficult to study predictors of recurrence. Both patients who experienced a recurrence of disease within 1 year had increased tumor volume radiographically during the treatment period. On-treatment tumor growth should be considered as a possible indicator of a higher risk of recurrence after surgery in larger, future neoadjuvant studies. Additionally, given the limitations of the number of markers that could be studied together in a single slide, we were unable to perform in-depth relationship analyses between subsets of myeloid cells and proliferating T cells. Such data could provide important correlative data supporting the immunosuppressive role of myeloid cells in these tumors, and experimental limitations for such work could be overcome with emerging spatial proteomic technologies that would allow the study of dozens of markers per slide.

Our current study provides a framework for experimental approaches that can be used to study important correlates that aid in understanding the mechanism(s) of neoadjuvant treatments, such as immune cell infiltration and spatial localization in tumor sections as well as tumor antigen–specific T cell responses in cultured TILs. Given the safety, early efficacy, and correlatives indicating enhancement of neoepitope-specific T cell responses we observed following treatment with bintrafusp alfa, future HNSCC clinical studies assessing whether similar findings are observed following PD-(L)1 blockade without TGF-β neutralization are warranted. Such studies could inform the design of future neoadjuvant immunotherapy trials that would maximally balance safety and enhance antitumor immunity for this highly lethal disease.

## Methods

### Study design and population.

Eligible patients had previously untreated, surgically resectable, pathologically confirmed T2-4b oral cavity or laryngeal squamous cell carcinoma, with or without nodal disease and no evidence of distant metastasis. All patients were evaluated by a head and neck oncologic surgeon at their home institution before referral to the NIH. Patients received 1 or 2 doses of bintrafusp alfa (1,200 mg) intravenously at weeks 1 and 3, followed by surgery. Surgical resection of the primary and nodal disease was performed based on initial clinical staging and was not altered on the basis of the immunotherapy response. The use of adjuvant therapy occurred off-study and was based on pathologic analysis of surgical specimens according to the standard of care as determined by the referring teams. Primary tumors were sampled for correlative analysis 1 day before initiation of bintrafusp alfa treatment and after completion of the treatment on protocol. LNs were not sampled.

### pTR.

Digital pathology software (QuPath) assessment of H&E slides from the surgical specimen was used to quantify the pTR in the primary tumor and LNs, defined as the percentage of viable tumor area within the tumor bed (surface area of residual viable tumor/surface area of total tumor bed × 100) (15, 16, 22). All H&E slides from each block of each surgical specimen were assessed, and a weighted (by surface area) mean was determined for each patient, rounded to the nearest 1 percent. Primary tumors and LNs were separately scored. Clinically or radiographically suspicious LNs were not biopsied before treatment. For each patient with LNs positive for carcinoma pathologically, all H&E slides from all positive LNs were used to determine the PR. A pCR was defined as no residual viable tumor; a major pathologic response was defined as 10% or less residual viable tumor (or a pTR ≥90%); a pPR was defined as 11%–50% residual viable tumor (or pTR 50%–89%); and no response was defined as greater than 50% residual viable tumor (or pTR <50%). For correlative studies, a PR was defined as a pTR of 50% or higher. Patients with suspicious LNs according to pretreatment imaging and no pathologic evidence of carcinoma within LNs were considered to have a possible LN CR. Two pathologists independently scored all slides for each patient’s surgical specimens.

### Radiographic response.

Primary and nodal disease was assessed radiographically by CT with contrast and, in some cases, by PET. Primary tumors were defined as lesions corresponding on examination to the site of the primary tumor that was visible on CT, and suspicious LNs were defined as those with at least 1 perpendicular dimension of 10 mm or greater or fluordeoxyglucose (FDG) avidity on PET. For patients with LNs positive for carcinoma pathologically, the volume of all positive LNs before and after treatment was considered. For patients that had suspicious LNs before treatment but had pathologically negative results, the volume of the clinically suspicious LN only was considered. Volume was defined as the product of the longest perpendicular bidirectional measurements for primary tumors and LNs (9).

### WES and RNA-Seq.

Adequate tumor fraction was verified in each pre- and post-treatment biopsy before nucleic acid extraction and sequencing. DNA-Seq libraries were captured to exome regions using xGen Exome Research Panel, version 1.0 (IDT), and libraries were prepared using the KAPA HyperPrep Kit (Kapa Biosystems). DNA libraries were sequenced to a target depth of ×200 for tumor samples and ×100 for normal samples on the Illumina HiSeq platform. RNA-Seq libraries were prepared using the KAPA Stranded RNA-Seq Kit with RiboErase (Kapa Biosystems) and sequenced to a target depth of 200-M reads on the Illumina HiSeq platform (Illumina). All genomic data are available through the Database of Genotype and Phenotypes (dbGaP) under accession number phs002849.v1.p1.

### Genomic and transcriptomic analysis.

Paired-end reads, obtained from whole-exome sequencing, were subjected to adapter trimming using TrimGalore (https://github.com/FelixKrueger/TrimGalore). The resulting reads were aligned to the hg38 reference genome using bwa-mem2 (https://doi.org/10.1109/IPDPS.2019.00041) with default parameters. Fixmate, sort, and markdup of the SAMtools toolkit were used to convert SAM files to the BAM format and include pairing information, sort by genomic coordinates, and mark duplicates, respectively, with default parameters. Base quality scores were recalibrated using GATK26 BaseRecalibrator and ApplyBQSR.

Somatic single nucleotide variants (SNVs) and small insertions and deletions (INDELs)were identified using an ensemble calling strategy (36). SNV calling was performed using the following 4 variant callers: LoFreq (version 2.1.5) (37), MuSE (version 1.0rc) (38), Mutect2 (https://doi.org/10.1101/861054; GATK version 4.1.9.0), and Strelka2 (version 2.9.10) (39) with default parameters unless stated otherwise. In each comparison, tumor or mucosa and the corresponding control (PBMC) BAM files were provided as input. Before running lofreq somatic using –call-indels, input BAM files were subjected to lofreq indelqual –dindel. Strelka2 was run using –indelCandidates obtained from Manta (version 1.6.0) (40). Furthermore, GATK Mutect2 calls were filtered using GATK FilterMutectCalls. Final SNV calls were made by integrating the results and retaining only SNVs that were detected by at least 3 of the 4 variant callers. Similarly, only INDELs that were detected by at least 2 of the 3 variant callers — LoFreq, Mutect2, and Strelka2 — were retained. Where needed, common SNPs in dbsnp_146.hg38.vcf.gz of the GATK resource bundle were used. Variant effect prediction was performed using VEP (version 101) (41). The resulting VCF files were converted to MAF using vcf2maf (https://doi.org/10.5281/zenodo.593251). Downstream data analysis and visualization were performed using R (https://www.R-project.org/) and the R packages maftools (42), tidyverse (https://doi.org/10.21105/joss.01686), and ComplexHeatmap. (43). Analysis of copy number alterations was performed using the R package SuperFreq (44) with default parameters.

Paired-end reads obtained from RNA-Seq were subjected to adapter trimming using TrimGalore (https://github.com/FelixKrueger/TrimGalore). To quantify gene expression, the resulting reads were provided to RSEM (45) and aligned to a custom reference containing human (GRCh38; GENCODE, version 32) transcripts. Transcripts per million (TPM) values were log_2_-transformed after adding 1.

### Multispectral immunofluorescence.

Formalin-fixed, paraffin-embedded tumors were sectioned at 5 microns, baked at 60°C for 30 minutes, soaked in Bond Dewax Solution (Leica), and rehydrated. Deparaffinization and staining of all slides was performed on the Leica BOND RX Autostainer (Leica). Before being used in combination, the specificity and optimal dilution of each antibody was individually determined with chromogenic immunohistochemistry (DAB) using slides from normal tonsil and head and neck carcinoma, consistent with best practice guidelines (46). Heat-induced epitope retrieval was performed by heating to 95°C in BOND epitope retrieval solutions ER1 or ER2 (Leica). Tyramide signal amplification (TSA) Opal technology was used for immunofluorescence staining. After individual primary antibody optimization, primary and secondary antibody and opal pairings were optimized for minimum background and desired signal amplification in monoplex immunofluorescence using head and neck carcinoma sections. Supplemental Table 3 lists the primary and secondary antibodies used and the Opal details. Slides were coverslipped using the Leica CV5030 automated coverslipper after staining. Whole-slide images were obtained at ×40 magnification using 7-color, whole-slide unmixing filters on a Vectra Polaris. All paired pre- and post-treatment tumors were stained and scanned concurrently.

### Immunofluorescence analysis.

Whole-slide analysis of each stained slide was performed with HALO Image Analysis software (version 3.3, Indica Labs). Tumor, stroma, or whole-slide annotations were performed using the Random Forest Tissue Classifier Algorithm. Standard nuclear segmentation was used for the TGF-β panel. The HALO AI Nuclear Segmentation Classifier was trained to identify nuclei of various sizes and used for the T cell and myeloid cell panels. The immune cell fraction within the stromal compartment was estimated by calculating the fraction of cytokeratin-negative cells with rounded morphology and a maximum diameter of 15 μm. Fluorescence intensities of each marker for each cell were determined using the HALO Highplex FL Analysis Algorithm. Separate Highplex FL Analysis Algorithms were used for tumor and immune cells, given the differences in nucleus size. Common fluorescence thresholds used to assign positivity or scaled intensity (1+, 2+, or 3+) for a given marker were used for each patient’s paired pre- and post-treatment tumor. Cell density was defined as the absolute number of lineage or activation marker–positive cells per unit area (mm^2^). The H-score was used to describe protein expression in cells and was defined as follows: (% 1+ cells × 1) + (% of 2+ cells × 2) + (% of 3+ cells × 3) for a range of 0–300. Spatial analysis between CD8^+^ or CD4^+^ T cells and Tregs was performed using the HALO Proximity Analysis Algorithm on spatial plots generated from T cell and Treg object cell *x* and *y* coordinates.

### TIL culture.

Tumor specimens were divided into 1 mm^3^ fragments and cultured in 2 mL media consisting of 50% AIM V media, 50% RPMI media, 25 mM HEPES buffer, 5% heat-inactivated human serum, 100 U/mL penicillin, 100 μg/mL streptomycin, 10 μg/mL gentamycin, 2 mM l-glutamine, 1.25 μg/mL amphotericin B, and 6,000 U/mL IL-2 at 37°C with 5% CO_2_. On days 5 and 8, one-half of the media were removed and replaced with fresh media and were split as needed on the basis of lymphocyte confluence. Day-14 TILs were harvested, enriched for CD8^+^ lymphocytes via negative magnetic selection using the EasySep Human T Cell Isolation Kit according to the manufacturer’s recommendations (STEMCELL Technologies), and cryopreserved in heat-inactivated FBS with 10% (v/v) DMSO. Successful TIL cultures were established using pre- and post-treatment tumor biopsies from 12 patients.

### Purification and expansion of autologous B cells.

B cells were isolated form PBMCs via positive magnetic selection using the EasySep Human CD19 Positive Selection Kit according to the manufacturer’s recommendations (STEMCELL Technologies) and cocultured with irradiated (6,000 rad) NIH3T3-CD40L feeder cells at a 1:1 ratio (5 × 10^6^ cells each) in 20 mL complete B cell media consisting of IMDM, 10% heat-inactivated human serum, 100 U/mL penicillin, 100 μg/mL streptomycin, 5 μg/mL gentamycin, 2 mM l-glutamine, and 200 U/mL IL-4 at 37°C with 5% CO_2_. Media were freshened on day 3 by adding an additional 10 mL media. Expanded B cells were harvested and counted on day 7 and cryopreserved in heat-inactivated FBS with 10% (v/v) DMSO.

### Neoepitope selection.

Neoepitopes were identified by constructing all possible permutations of 9-mer amino acid sequences derived from an identified nonsilent SNV or indel. Neoepitopes were ranked by RNA expression as well as allele frequency of the observed coding variant. NetMHC 3.4 was used to predict neoepitope binding to all patient-specific HLA class I alleles. Candidate neoepitopes were selected for synthesis (Peptide 2.0) according to the following criteria: (a) present in the pretreatment sample; (b) an IC_50_ of 200 nM or less; and (c) a TPM count of 4 or higher.

### Coculture assays.

Cryopreserved TILs and expanded B cells were thawed and rested overnight in T or B cell media, respectively, at 37°C with 5% CO_2_. B cells (4 × 10^5^ cells) were suspended in 1 mL complete B cell media and incubated with individual synthesized neoepitopes or pooled viral peptides (CEF Peptide Pool/HLA Class I Control; STEMCELL Technologies) at a final concentration of 0.1 μg/mL at 37°C with 5% CO_2_ for 1 hour. B cells were then irradiated (2,000 rad) and washed twice in complete B cell media. Peptide-pulsed B cells (4 × 10^4^) were cocultured with TIsL (2 × 10^4^, 2:1 effector/target [E/T] ratio) overnight at 37°C with 5% CO_2_. A human IFN-γ ELISpot (R&D Systems) assay was performed according to the manufacturer’s recommendations. Spot counts for the ELISpot assays were done on an Immunospot ELISpot plate reader (Cellular Technology). The ELISpot coculture assay was performed in a separate 96-well plate for each patient. Autologous expanded B cells pulsed with HPV 16 E7_11–19_ peptide and peripheral blood T cells engineered to express an HPV 16 E7-specific HLA-A*02–restricted T cell receptor (TCR) from a separate patient (47) were included as an additional positive control for each assay. Each condition was performed in technical duplicate, and mean spot counts are reported. Mean post counts of 5 or higher were considered positive.

### Statistics.

Correlations between paired sets of data were analyzed by linear regression and a goodness-of-fit test. Differences between nonparametric sets of data were analyzed with a 2-tailed Mann-Whitney *U* test. Differences between parametric sets of paired data were analyzed with the Wilcoxon signed-rank test. Comparisons of observed changes in immune cell infiltration with a hypothetical mean were analyzed with a 1-sample *t* test. A *P* value of less than 0.05 was considered significant. In all box-and-whisker plots, the horizontal line inside each box indicates the median, the top and bottom of the box indicate the IQR, and error bars indicate the fifth and 95th percentile values. Some correlations were compared with a linear regression goodness-of-fit test. Analyses were performed using GraphPad Prism, version 8.0.0 (GraphPad Software).

### Study approval.

This study (NCT04247282) was approved by the institutional review board of the NIH, and each patient provided written informed consent. The study protocol is available in the supplemental material.

## Author contributions

JMR, JF, YR, CS, WL, JS, JLG, and CTA conceived and designed the studies. JMR, JF, YR, CS, XY, WL, AS, APS, CCL, JLM, ET, WM, AJ, NRL, MP, RT, SH, AN, PSS, JS, JLG, and CTA generated data and provided key reagents and samples. JMR, JF, YR, CS, WL, AS, APS, CCL, JLM, ET, WM, AN, PSS, JS, JLG, and CTA analyzed and interpreted the data. JMR, JF, YR, CS, XY, WL, AS, APS, CCL, JLM, ET, WM, AJ, NRL, MP, RT, SH, AN, PSS, JS, JLG, and CTA analyzed, wrote, and revised the manuscript. JMR and JF are co–first authors. The order of their names was determined by the fact that JMR served as the principal investigator of this clinical trial, and JF performed much of the experimental work. All authors approved the final version of the manuscript.

## Supplementary Material

Supplemental data

Trial reporting checklists

ICMJE disclosure forms

## Figures and Tables

**Table 2:**
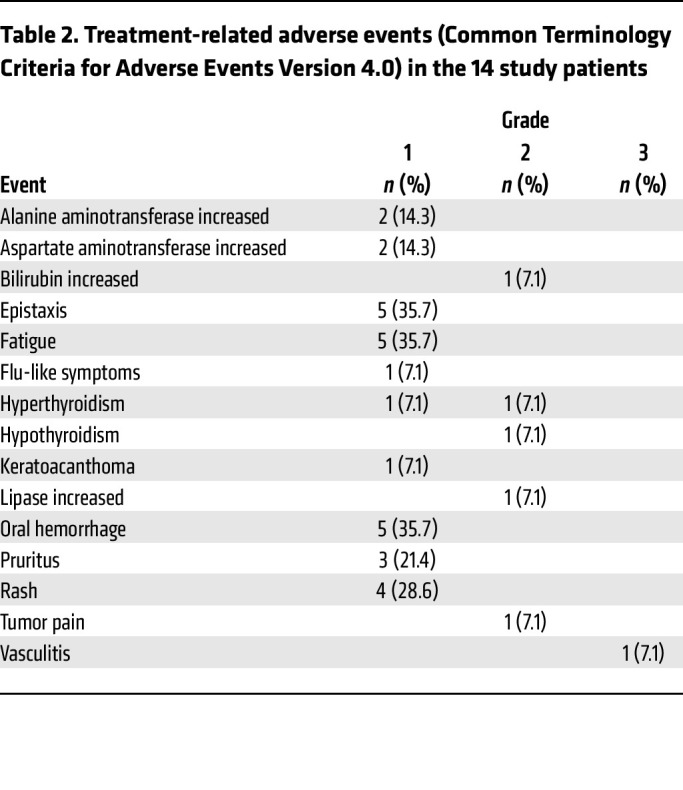
Treatment-related adverse events (Common Terminology Criteria for Adverse Events Version 4.0) in the 14 study patients

**Table 1:**
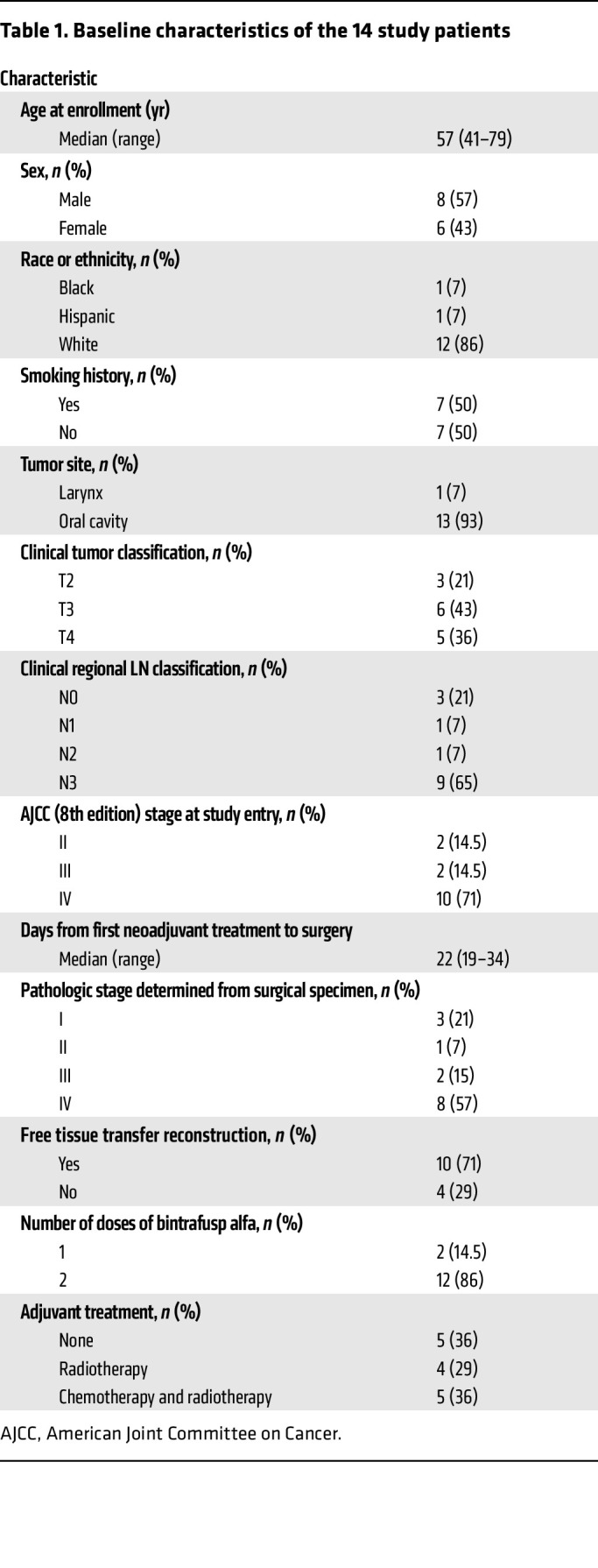
Baseline characteristics of the 14 study patients

## Version 1 06/21/2022

In-Press Preview

## Version 2 09/15/2022

Electronic publication
