# Enhanced neoepitope-specific immunity following neoadjuvant PD-L1 and TGF-β blockade in HPV-unrelated head and neck cancer

**DOI:** 10.1172/JCI172059

**Published:** 2023-06-01

**Authors:** Jason M. Redman, Jay Friedman, Yvette Robbins, Cem Sievers, Xinping Yang, Wiem Lassoued, Andrew Sinkoe, Antonios Papanicolau-Sengos, Chyi-Chia Lee, Jennifer L. Marte, Evrim Turkbey, Wojtek Mydlarz, Arjun Joshi, Nyall R. London, Matthew Pierce, Rodney Taylor, Steven Hong, Andy Nguyen, Patrick Soon-Shiong, Jeffrey Schlom, James L. Gulley, Clint T. Allen

Original citation: *J Clin Invest*. 2022;132(18):e161400. https://doi.org/10.1172/JCI161400

Citation for this corrigendum: *J Clin Invest*. 2023;133(11):e172059. https://doi.org/10.1172/JCI172059

After the publication of this paper, the authors became aware of errors in data analysis due to an issue with the barcoding of samples that caused some samples to be excluded. The authors have repeated all of the analysis and corrected Supplemental Figures 5, 6, and 9. The supplemental file has been updated online.

The authors regret the errors.

## Version 1 06/01/2023

Electronic publication

