# Supplementary material for: Enhanced neoepitope-specific immunity following neoadjuvant PD-L1 and TGF-β blockade in HPV-unrelated head and neck cancer
Source: J Clin Invest. 2022 Sep 15;132(18):e161400. doi: 10.1172/JCI161400 (PMC9479764; doi:10.1172/JCI161400)
Supplement: Supplemental data [file jci-132-161400-s125.pdf]

Supplemental Tables and Figures

Supplemental Table I. MIF immune cell phenotype markers

Supplemental Table II. Predicted neoepitopes selected for synthesis and experimental study

Supplemental Table III. MIF reagents

Supplemental Figure 1 – Examples of primary tumor histology and pTR calculations

Supplemental Figure 2 – Imaging and histology from patients that had clinically suspicious nodal disease but were pathologically N0.

Supplemental Figure 3 – Discordant pathologic responses

Supplemental Figure 4 – Workflow and examples of multispectral immunofluorescence

Supplemental Figure 5 – Oncoplot of frequently mutated genes

Supplemental Figure 6 – TGF-β pathway genomic and transcriptomic analysis

Supplemental Figure 7 – Tumor PD-L1 expression

Supplemental Figure 8 – Distribution of Ki67 negative T cells around Tregs

Supplemental Figure 9 – Transcriptomic immune profiling of tumors

Supplemental Figure 10 – Viral-specific TIL responses

Supplemental Table I. MIF immune cell phenotype markers

Phenotype	MIF markers
CD8 T cells	CK- CD8+ CD4- FoxP3- PD-L1+/- Ki67+/-
CD4 T cells	CK- CD8- CD4+ FoxP3- PD-L1+/- Ki67+/-
Tregs	CK- CD8- CD4+ FoxP3+ PD-L1+/- Ki67+/-
Total myeloid cells	CD11b+ or CD33+
Neutrophilic cells	CD11b+ or CD33+ CD15+ $CD68^{low}$ PD-L1+/-
Macrophages	CD11b+ or CD33+ CD15- $CD68^{high}$ PD-L1+/-

Supplemental Table II

Patient	Neoepitope number	hugo gene name	mutation_type	protein_change	base_change	wild type	neoepitope	allele_frequency	TPM	IC50	HLA_Allele	IFNg spot count pre/post
2	1	NOTCH1	Missense	p.C398Y	C->T	AICTCPSGY	AIYTCPSGY	0.05	30.13	39.00	HLA-A03:01	0/0
	2	PNPLA6	Missense	p.V590M	G->A	VAARMSPFV	VAARMSPFM	0.0846560846561	26.82	6	HLA-C03:03	0/0
	3	UBE4A	Missense	p.V346A	T->C	VVENHGYFL	VAENHGYFL	0.061170212766	22.88	20	HLA-C03:03	0/0
	4	FIG4	Missense	p.L401R	T->G	AAVTYLNQF	AAVTYRNQF	0.111842105263	6.01	170	HLA-C03:03	0/92
	5	OSBP2	Missense	p.S636L	C->T	YFSELALTL	YFLELALTL	0.0516795865633	4.11	86	HLA-C07:02	0/0
3	1	MAL2	FrameShiftDel	p.V23Afs*51	GT->G	PPRVTLPAG	PPRAPCPPA	0.0227272727273	76.24	132	HLA-B07:02	0/0
	2	PRDM1	Missense	p.P411R	C->G	SYNAHYPKF	SYNAHYRKF	0.0878243512974	38.86	39	HLA-C07:02	0/0
	3	PRDM1	Missense	p.P411R	C->G	PSYNAHYPK	PSYNAHYRK	0.0878243512974	38.86	95	HLA-A11:01	0/0
	4	PRDM1	Missense	p.P411R	C->G	YPKFLLPPY	YRKFLLPPY	0.0878243512974	38.86	302	HLA-C07:02	0/23
	5	EBP	Missense	p.L183V	C->G	LYFWFYFVF	VYFWFYFVF	0.332258064516	38.35	59	HLA-C07:02	0/0
	6	FAM20C	Missense	p.H308Y	C->T	FYFSDYERH	FYFSDYERY	0.144736842105	23.63	22	HLA-C07:02	0/0
	7	FAM20C	Missense	p.H308Y	C->T	RHNAEIAAF	RYNAEIAAF	0.144736842105	23.63	87	HLA-C07:02	0/0
	8	ZMIZ2	Missense	p.P207S	C->T	GPRGPSVPA	GPRGPSVSA	0.121718377088	14.47	17	HLA-B07:02	0/131
	9	EIF2AK3	Missense	p.T177K	G->T	ESMETVPFT	ESMETVPFK	0.0934182590234	7.23	29	HLA-A11:01	0/16
	10	IRX1	Missense	p.A301T	G->A	SPGAAAGGL	SPGTAAGGL	0.0953608247423	4.12	31	HLA-B07:02	0/0
	11	PID1	Missense	p.E73Q	C->G	FHEPEAIEL	FHEPEAIQL	0.100263852243	4.09	166	HLA-C07:02	0/0
4	1	COL1A2	Missense	p.D1115H	G->C	DFGYDGDFY	DFGYDGHFY	0.0918544194107	125.56	31	HLA-A29:02	0/0
	2	HMHA1	Missense	p.S80L	C->T	FPLSGAASW	FPLLGAASW	0.0551989730424	39.12	118	HLA-B07:02	0/0
	3	PTPN11	Missense	p.I514V	A->G	FIYMAVQHY	FVYMAVQHY	0.0932069510269	21.78	19	HLA-A29:02	0/0
	4	PTPN11	Missense	p.I514V	A->G	TEAQYRFIY	TEAQYRFVY	0.0932069510269	21.78	135	HLA-B44:03	0/0
	5	PTPN11	Missense	p.I514V	A->G	IYMAVQHYI	VYMAVQHYI	0.0932069510269	21.78	9	HLA-A24:02	0/0
	6	FNIP1	Missense	p.P516A	G->C	PVRLARTVV	AVRLARTVV	0.130353817505	18.55	24	HLA-B07:02	0/0
	7	NOP58	Missense	p.V246I	G->A	AEISMGTEV	AEISMGTEI	0.0911854103343	14.58	15	HLA-B44:03	0/0
	8	EMB	Missense	p.S324Y	G->T	VPRHRKNES	VPRHRKNEY	0.0844594594595	10.37	148	HLA-B07:02	0/0
	9	MED26	Missense	p.Q493H	C->G	SYLSRQSSL	SYLSRHSSL	0.060475161987	8.82	104	HLA-C07:02	0/0
	10	ZNF865	FrameShiftIns	p.A61Gfs*18	C->CG	LPCAPGPPP	LPCGPRPPA	0.032967032967	4.91	142	HLA-B07:02	0/0
	11	FAM13A	Missense	p.R738Q	C->T	RMRQRSNTL	RMRQQSNTL	0.0643776824034	4.14	40	HLA-B07:02	0/0
6	1	SF3B2	Missense	p.M296I	G->A	METDARSSL	IETDARSSL	0.145161290323	77.74	50	HLA-C03:03	0/0
	2	GAK	Missense	p.R478W	G->A	SECGWAARR	SECGWAARW	0.0634920634921	28.15	48	HLA-B44:02	0/11
	3	GAK	Missense	p.R478W	G->A	WAARRAPHL	WAARWAPHL	0.0634920634921	28.15	9	HLA-C03:03	21/15
	4	EXOSC6	Missense	p.Q15R	T->C	EESQPPQLY	EESRPPQLY	0.0447154471545	27.95	80	HLA-B44:02	0/10
	5	COL14A1	Missense	p.D1297H	G->C	FLFRILPDT	FLFRILPHT	0.0506329113924	23.96	11	HLA-A02:01	0/18
	6	SMARCA2	Missense	p.Q1118K	C->A	FNEPGSQYF	FNEPGSKYF	0.0790960451977	16.89	177	HLA-C05:01	0/0
	7	FAM8A1	Missense	p.V356M	G->A	VLVIPSSNV	VLMIPSSNV	0.0874316939891	7.0	41	HLA-A02:01	0/37
	8	FAM8A1	Missense	p.V356M	G->A	LIAPSRVLV	LIAPSRVLM	0.0874316939891	7.0	160	HLA-C03:03	0/8
	9	ATXN1	Missense	p.D341N	C->T	YGAPSSADL	YGAPSSANL	0.107671601615	6.55	5	HLA-C03:03	0/33
	10	ZSCAN18	Missense	p.T377N	G->T	LADPPSGTT	LADPPSGNT	0.0865921787709	4.51	78	HLA-C05:01	0/0
7	1	GRHL2	Missense	p.R136G	C->G	LENSKREQY	LENSKGEQY	0.0576131687243	14.95	184	HLA-B44:03	9/111
	2	PHF12	Missense	p.R895C	G->A	VIRRRRHQK	VIRCRRHQK	0.0373134328358	7.64	58	HLA-A31:01	0/0
	3	PHF12	Missense	p.R895C	G->A	KVQSVIRRR	KVQSVIRCR	0.0373134328358	7.64	33	HLA-A31:01	0/0
	4	PHF12	Missense	p.R895C	G->A	VQSVIRRRR	VQSVIRCRR	0.0373134328358	7.64	25	HLA-A31:01	0/0
	5	ATR	Missense	p.E2426D	T->G	VFREFLLPR	VFRDFLLPR	0.0700280112045	5.49	13	HLA-A31:01	0/92
8	1	CTNNA1	Missense	p.I792V	A->G	ALYCHQLNI	ALYCHQLNV	0.026470588	136.8	13	HLA-A02:01	0/0
	2	USP5	Missense	p.M670V	A->G	QLVEMGFPM	QLVEMGFPV	0.134328358	31.08	7	HLA-A02:01	0/0
	3	TM9SF3	Missense	p.M556V	T->C	YMAVFSTAL	YVAVFSTAL	0.116666667	29.9	90	HLA-A02:01	0/0
	4	TM9SF3	Missense	p.P408L	G->A	LPLNLVGTI	LLLNLVGTI	0.033670034	29.9	144	HLA-A02:01	0/0
	5	TM9SF3	Missense	p.P408L	G->A	FVILPLNLV	FVILLLNLV	0.033670034	29.9	25	HLA-A02:01	0/0
	6	NOTCH1	Missense	p.G443D	C->T	FECQCLQGY	FECQCLQDY	0.023419204	16.81	84	HLA-B44:03	0/0
	7	CAMTA1	Missense	p.R811G	C->G	SEDGARAPF	SEDGAGAPF	0.035046729	14.41	139	HLA-B44:03	0/0
	8	GNPAT	Missense	p.L476F	C->T	MLQHITLLM	MLQHITLFM	0.047120419	9.41	34	HLA-A02:01	0/0
	9	SLC8B1	Missense	p.L309F	G->A	VRALNPLDY	VRAFNPLDY	0.026706231	8.58	140	HLA-A80:01	0/0
	10	SLC8B1	Missense	p.L309F	G->A	ILVRALNPL	ILVRAFNPL	0.026706231	8.58	93	HLA-A02:01	0/0
9	1	KIF26B	Missense	p.P1228H	C->A	RPVSIISSI	RHVSIISSI	0.0285132382892	18.21	140	HLA-C07:01	0/0
	2	KIF26B	Missense	p.P1228H	C->A	ALASGSRPV	ALASGSRHV	0.0285132382892	18.21	110	HLA-A02:01	0/0
	3	SYN1	Missense	p.S81P	A->G	FFSSLSNAV	FFSSLPNAV	0.0265780730897	4.86	155	HLA-C12:03	0/0
	4	ZNF141	Missense	p.A207P	G->C	KAFKWSLIF	KPFKWSLIF	0.0179153094463	4.7	142	HLA-C12:03	0/0
10	1	DERL1	Missense	p.L47R	A->C	FLWPEAFLY	FLWPEAFRY	0.0216216216216	22.78	2	HLA-A29:02	0/0
	2	PRKD3	Missense	p.P98S	G->A	PECGFFGMY	SECGFFGMY	0.0365853658537	6.54	163	HLA-A29:02	0/0
	3	RPUSD2	Missense	p.S471L	C->T	FEYFSPMPA	FEYFLPMPA	0.108552631579	5.86	81	HLA-B45:01	0/0
	4	TUBGCP6	Missense	p.Y403H	A->G	AEYGTCYTR	AEHGTCYTR	0.0335570469799	4.47	18	HLA-B45:01	0/0
	5	CSGALNAC	Missense	p.Y68S	A->C	EEHYQTRAT	EEHSQTRAT	0.0203488372093	4.03	48	HLA-B45:01	0/0
	6	CSGALNAC	Missense	p.Y68S	A->C	QEEHYQTRA	QEEHSQTRA	0.0203488372093	4.03	102	HLA-B45:01	0/0
11	1	CSDE1	InFrameDel	p.E181del	ACTT->A	YERNGEVFY	YERNGVFYL	0.183333333333	126.23	9	HLA-B40:01	8/53
	2	DST	FrameShiftDel	p.E1512Kfs*34	CTAATTCT->C	LEKFDADYT	SLMLTIPSL	0.163742690058	75.16	110	HLA-B08:01	0/0
	3	DST	FrameShiftDel	p.E1512Kfs*34	CTAATTCT->C	THSLQEELE	THSLQEKSL	0.163742690058	75.16	155	HLA-C07:01	0/0
	4	DST	FrameShiftDel	p.E1512Kfs*34	CTAATTCT->C	SLQEELEKF	SLQEKSLML	0.163742690058	75.16	190	HLA-B08:01	0/0
	5	RHOB	Missense	p.A132G	C->G	LARMKQEPV	LGRMKQEPV	0.0216606498195	70.42	180	HLA-B08:01	15/74
	6	ERBB3	Missense	p.M677I	G->A	RRIQNKRAM	RRIQNKRAI	0.120521172638	31.16	57	HLA-C07:01	0/0
	7	OLFML2A	Missense	p.N456S	A->G	WSNMYKLPY	WSSMYKLPY	0.0236406619385	20.25	7	HLA-A01:01	0/0
	8	GGA1	Missense	p.L43F	G->C	LELLYSWTV	FELLYSWTV	0.047619047619	13.06	103	HLA-B40:01	9/7
	9	CBLB	Missense	p.N466K	G->C	LMMNRLANV	LMMNRLAKV	0.212290502793	9.38	20	HLA-B08:01	0/0
	10	DOPEY2	Missense	p.Q810H	G->C	LQNVAISTL	LHNVAISTL	0.14979757085	8.36	31	HLA-C07:01	6/41
	11	PLAGL2	Missense	p.L372V	G->C	AELPGSLSL	AELPGSLSV	0.141104294479	7.99	73	HLA-B40:01	0/0
	12	PLXNA3	Missense	p.G878A	G->C	REVGLRVAG	REVGLRVAA	0.274678111588	4.73	161	HLA-B40:01	8/27
12	1	ZNF516	Missense	p.A725G	G->C	ALAPDLMPL	GLAPDLMPL	0.34338358459	11.12	141	HLA-C03:03	0/0
	2	ZNF174	FrameShiftDel	p.L9Ffs*4	TTAAGC->T	MAAKMEITL	MAAKMEITF	0.3165374677	4.79	63	HLA-C03:03	0/0
	3	IRX4	Missense	p.A403T	C->T	AAAAAATSL	TAAAAATSL	0.00778605280975	75.17	2	HLA-C03:03	0/0
	4	INTS4	Missense	p.K698T	T->G	LASAAAKQI	LASAAATQI	0.0546558704453	10.08	11	HLA-C03:03	0/0
	5	INTS4	Missense	p.K698T	T->G	ASAAAKQIM	ASAAATQIM	0.0546558704453	10.08	61	HLA-C03:03	0/0
	6	PODXL	InFrameIns	p.S31_Q32insPP	G->GGGGGGC	SQNATQTTT	AESPHLLLL	0.0346232179226	8.6	133	HLA-B44:03	0/0
	7	ACPT	Missense	p.L20V	C->G	LLVLPPRAL	LVVLPPRAL	0.00960960960961	19.48	6	HLA-C03:03	37/97
13	1	PABPC1	Missense	p.P362L	G->A	PLYVALAQR	LLYVALAQR	0.047263681592	614.88	104	HLA-A68:01	0/0
	2	PABPC1	Missense	p.P362L	G->A	RIVATKPLY	RIVATKLLY	0.047263681592	614.88	11	HLA-A29:02	0/0
	3	TLN1	Missense	p.P450H	G->T	SVALPAIMR	SVALHAIMR	0.0389294403893	37.53	18	HLA-A68:01	5/63
	4	TRADD	Missense	p.D157N	C->T	LAELEDALR	LAELENALR	0.0329153605016	25.24	129	HLA-A68:01	5/41
14	1	LUM	Missense	p.F193L	G->C	YLDLSFNQI	YLDLSLNQI	0.18904109589	95.75	35	HLA-A02:01	0/0
	2	PTBP1	Missense	p.I213L	A->C	SKFGTVLKI	SKFGTVLKL	0.063829787234	109.65	87	HLA-B39:01	0/0
	3	UBAP2L	Missense	p.H999Y	C->T	QSFEKQGFH	QSFEKQGFY	0.18345323741	31.09	43	HLA-A30:02	6/10
	4	SERPINE1	Missense	p.L139V	C->G	FMPHFFRLF	FMPHFFRVF	0.261044176707	16.16	36	HLA-C12:03	0/0
	5	SERPINE1	Missense	p.L139V	C->G	FFRLFRSTV	FFRVFRSTV	0.261044176707	16.16	40	HLA-C12:03	0/0
	6	AMPD2	Missense	p.T188M	C->T	RAKQDFLKT	RAKQDFLKM	0.321705426357	12.76	185	HLA-C12:03	0/0
	7	MFSD13A	Missense	p.R98W	C->T	VVLARVQAL	VVLAWVQAL	0.267973856209	6.49	89	HLA-A02:01	0/0
	8	COL15A1	Missense	p.S20F	C->T	LLMLLSVST	LLMLLSVFT	0.0774818401937	18.01	140	HLA-A02:01	0/0
	9	COL15A1	Missense	p.S20F	C->T	MLLSVSTPL	MLLSVFTPL	0.0774818401937	18.01	4	HLA-A02:01	6/58
	10	COL15A1	Missense	p.S20F	C->T	SVSTPLPAV	SVFTPLPAV	0.0774818401937	18.01	26	HLA-A02:01	0/0
	11	ALDH7A1	Missense	p.L216V	G->C	AMICGNVCL	AMICGNVCV	0.114814814815	12.09	14	HLA-A02:01	0/0
	12	TPGS1	Missense	p.V176L	G->C	AVPLSVFRA	ALPLSVFRA	0.216279069767	6.03	191	HLA-A02:01	0/0
	13	TPGS1	Missense	p.V176L	G->C	CRDHEAVPL	CRDHEALPL	0.216279069767	6.03	32	HLA-B39:01	0/0
	14	BID	Missense	p.R168H	C->T	LLRDVFHTT	LLHDVFHTT	0.0463157894737	23.26	111	HLA-A02:01	0/0
	15	BID	Missense	p.R168H	C->T	SLLRDVFHT	SLLHDVFHT	0.0463157894737	23.26	36	HLA-A02:01	0/0
	16	LRRC15	Missense	p.N249K	G->C	GLFHNNHNL	GLFHKNHNL	0.107329842932	7.87	35	HLA-A02:01	0/18
	17	ZMIZ2	Missense	p.C516G	T->G	VCQPGRNTI	VGQPGRNTI	0.037422037422	15.71	110	HLA-C12:03	0/0
	18	MORC3	Missense	p.T750S	C->G	HQSTETDAV	HQSTESDAV	0.0345622119816	16.84	42	HLA-B39:01	0/0
	19	TMC4	Missense	p.L407V	G->C	FVLPPVFKL	FVLPPVFKV	0.0528169014085	9.52	8	HLA-A02:01	0/0
	20	TMC4	Missense	p.L407V	G->C	KLIAPLEGY	KVIAPLEGY	0.0528169014085	9.52	128	HLA-A30:02	0/0
	21	SERPINB8	Missense	p.V264L	G->C	KVQVFLPRL	KLQVFLPRL	0.078231292517	5.71	25	HLA-A02:01	0/0
	22	RLIM	Missense	p.T429I	G->A	YSDSDSEPT	YSDSDSEPI	0.0412087912088	8.52	27	HLA-C12:03	0/0
	23	FUK	Missense	p.R106T	G->C	FPFDDCGRA	FPFDDCGTA	0.0287769784173	5.91	31	HLA-C12:03	0/0

Supplemental Table III. MIF reagents.

TGFβ staining panel

**Antibody**	**Vendor /Clone**	**Catalog #**	**Dilution :**	**Epitope retrieval**	**Secondary**	**Opal**	**Opal dilution:**
CD15	BD Bioscience/ HI98	555400	1500	NA	Vector Labs-ImmpreSS mouse-HRP (MP-7452)	520	150
CD11b	Abcam/ EPR1344	ab133357	5000	ER1/20min	Akoya Opal Polymer (Akoya Biosciences cat no. ARH1001EA)	480	150
CD33	Abcam/ SP266	ab199432	200	ER2/20min	Akoya Opal Polymer (Akoya Biosciences cat no. ARH1001EA)	690	150
CD68	Invitrogen/ KP1	MA5-13324	1000	ER1/20min	Akoya Opal Polymer (Akoya Biosciences cat no. ARH1001EA)	620	150
PD-L1	CST/ EL13N	13684S	300	ER2/20min	MACH2 (Biocare medical, cat no:RHRP520)	570	150
Pan-Cytokeratin	Santa Cruz/ AE1/AE3	sc-81714	400	ER2/20min	Akoya Opal Polymer (Akoya Biosciences cat no. ARH1001EA)	TSA DIG-opal780	1:50 Opal 780/1:100 TSA-DIG

**Antibody**	**Vendor /Clone**	**Catalog #**	**Dilution :**	**Epitope retrieval**	**Secondary**	**Opal**	**Opal dilution:**
CD4	Abcam/ EPR6855	ab133616	1000	ER1/20 min	MACH2 (Biocare medical, cat no:RHRP520)	520	150
CD8	ABCAM/ EPR10640-2	ab215041	2000	ER2/20 min	Akoya Opal Polymer (Akoya Biosciences cat no. ARH1001EA)	570	150
FOXP3	Invitrogen/ SP97	MA5-16365	200	ER2/20 min	MACH2 (Biocare medical, cat no:RHRP520)	480	150
PDL1	Cell Signaling/ EL13N	13684S	300	ER2/20 min	MACH2 (Biocare medical, cat no:RHRP520)	620	150
Ki67	Ventana/ 790-4286	790-4286	1	ER1/20 min	MACH2 (Biocare medical, cat no:RHRP520)	690	150
Pan-cytokeratin	Santa Cruz/ AE1/AE3	sc-81714	400	ER2/20 min	Akoya Opal Polymer (Akoya Biosciences cat no. ARH1001EA)	TSA DIG-opal780	1:50 Opal 780/1:100 TSA-DIG

T cell staining panel

Antibody	Vendor /Clone	Catalog #	Dilution :	Epitope retrieval	Secondary	Opal	Opal dilution:
CD15	BD Bioscience/ HI98	555400	1500	NA	Vector Labs- ImmpreSS mouse-HRP (MP-7452)	520	150
CD11b	Abcam/ EPR1344	ab133357	5000	ER1/20min	Akoya Opal Polymer (Akoya Biosciences cat no. ARH1001EA)	480	150
CD33	Abcam/ SP266	ab199432	200	ER2/20min	Akoya Opal Polymer (Akoya Biosciences cat no. ARH1001EA)	690	150
CD68	Invitrogen/ KP1	MA5-13324	1000	ER1/20min	Akoya Opal Polymer (Akoya Biosciences cat no. ARH1001EA)	620	150
PD-L1	Cell Signaling/ EL13N	13684S	300	ER2/20min	MACH2 (biocare medical, cat no:RHRP520)	570	150
Pan-Cytokeratin	Santa Cruz/ AE1/AE3	sc-81714	400	ER2/20min	Akoya Opal Polymer (Akoya Biosciences cat no. ARH1001EA)	TSA DIG-opal780	1:50 Opal 780/1:100 TSA-DIG

Myeloid staining panel

Supplemental Figures and Legends

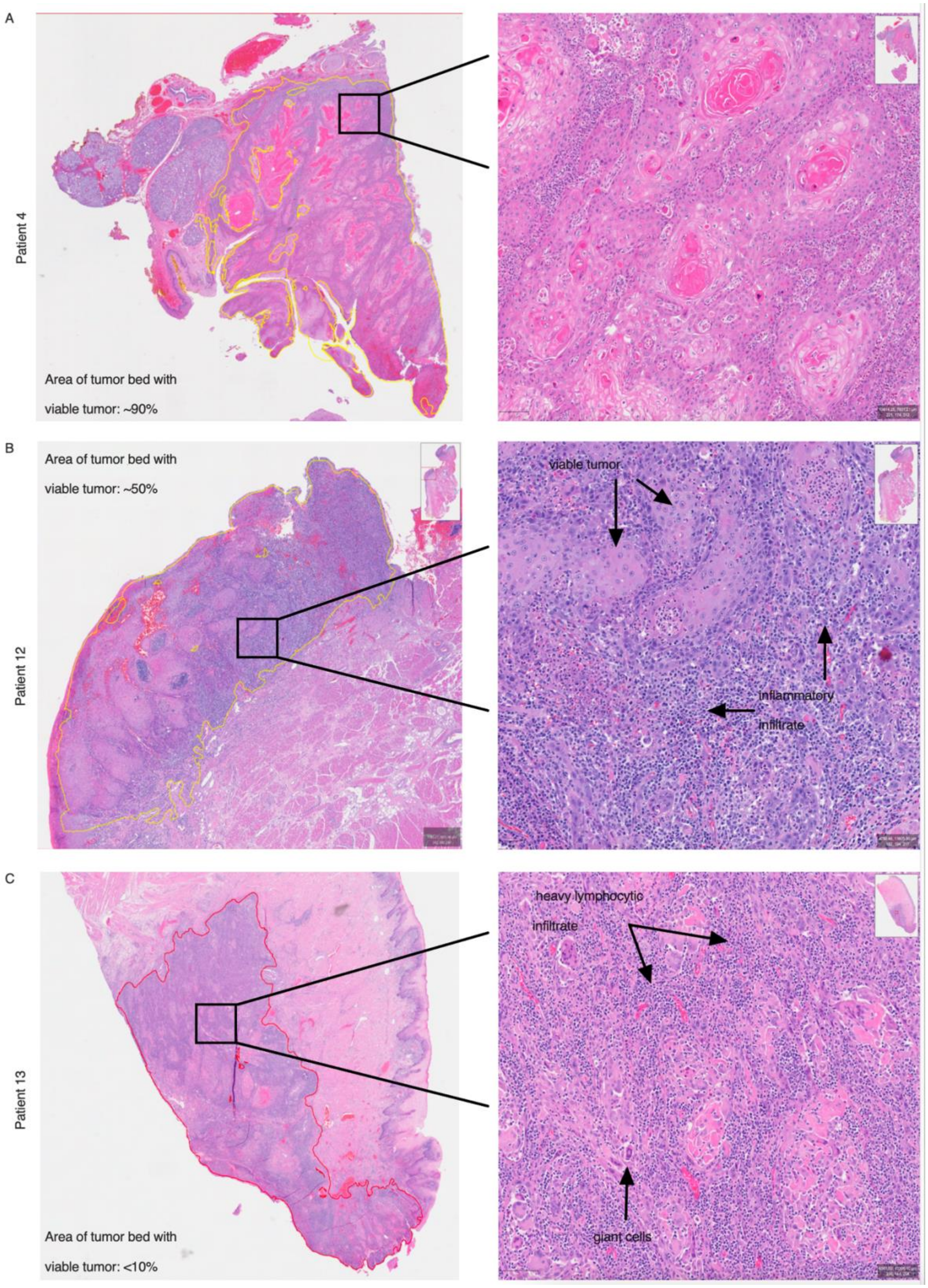

Supplemental Figure 1 – Examples of primary tumor histology and pTR calculations

A, representative low magnification (left) and high magnification (right) photomicrographs of hematoxylin and eosin (H&E)-stained tumor sections from patient 4 that demonstrated a pTR of 3. The yellow line indicates the tumor bed.

B, representative photomicrographs of H&E-stained tumor sections from patient 12 that demonstrated a pTR of 43. The yellow line indicates the tumor bed. Regions of viable or apoptotic tumor cells are indicated by the black arrows.

C, representative photomicrographs of H&E-stained tumor sections from patient 13 that demonstrated a pTR of 68. The red line indicates the tumor bed. Regions of giant cells and heavy lymphocytic infiltrate are indicated by the black arrows.

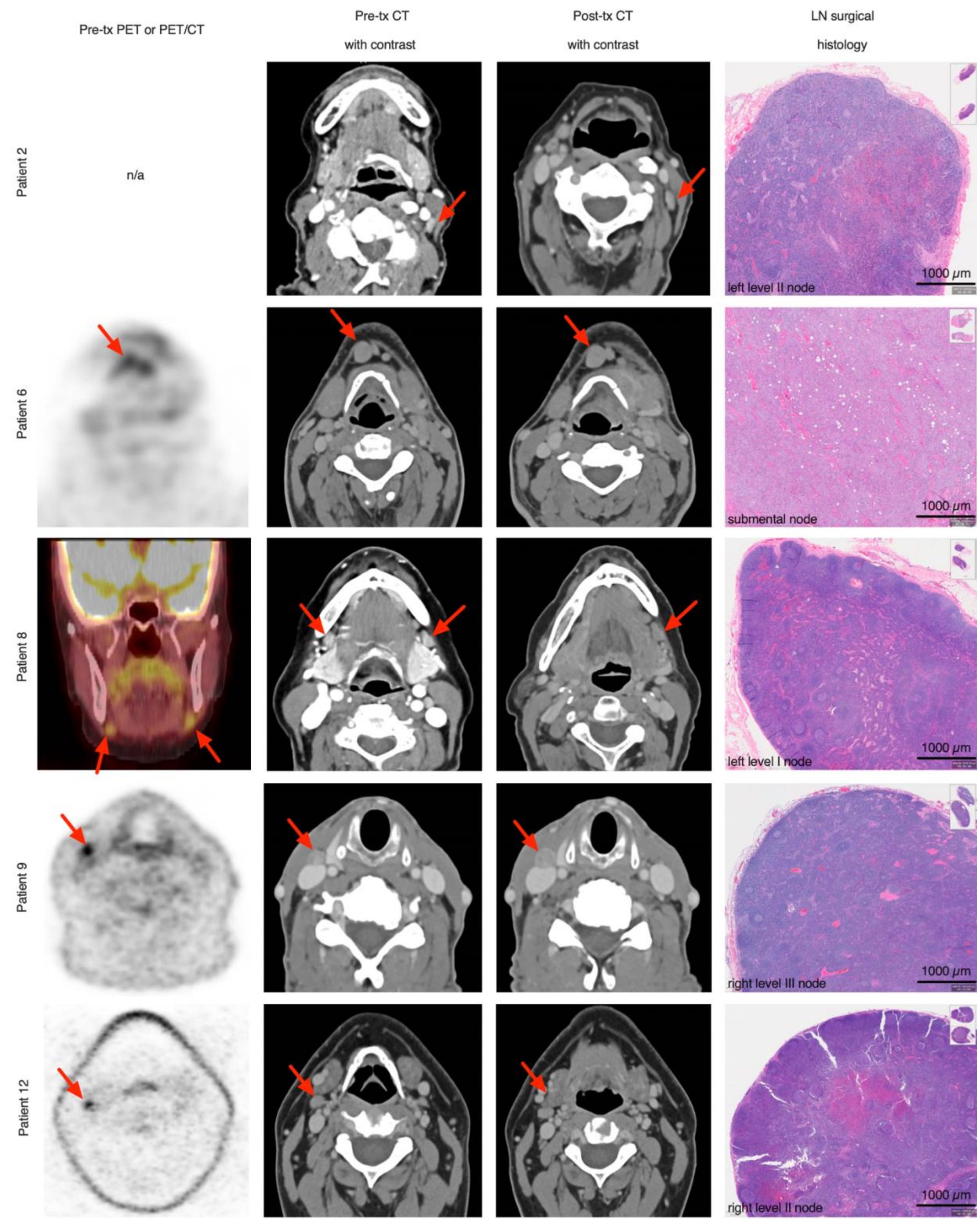

Supplemental Figure 2 – Imaging and histology from patients that had clinically suspicious nodal disease but were pathologically N0.

Representative pre-treatment PET (when available, left column) and CT imaging (left middle column), post-treatment CT imaging (right middle column) and photomicrographs of H&E-stained LN section from the corresponding LN (right column) for patients 2, 6, 8, 9 and 12. Red arrows indicate suspicious pre-treatment and post-treatment LNs. H&E LN photomicrographs are H&E stained sections from the same LNs indicated by the red arrows.

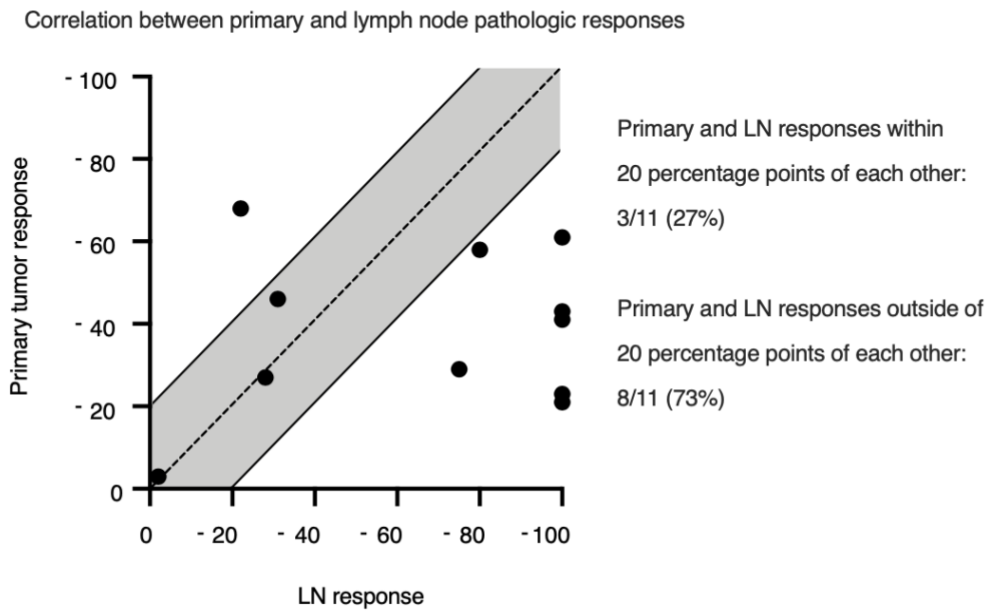

Supplemental Figure 3 – Discordant pathologic responses

Dot plot shows the correlation between primary tumor and LN pTR for 11 patients with suspicious LNs. The shaded grey area highlights samples that fall within 20 percentage points of the diagonal.

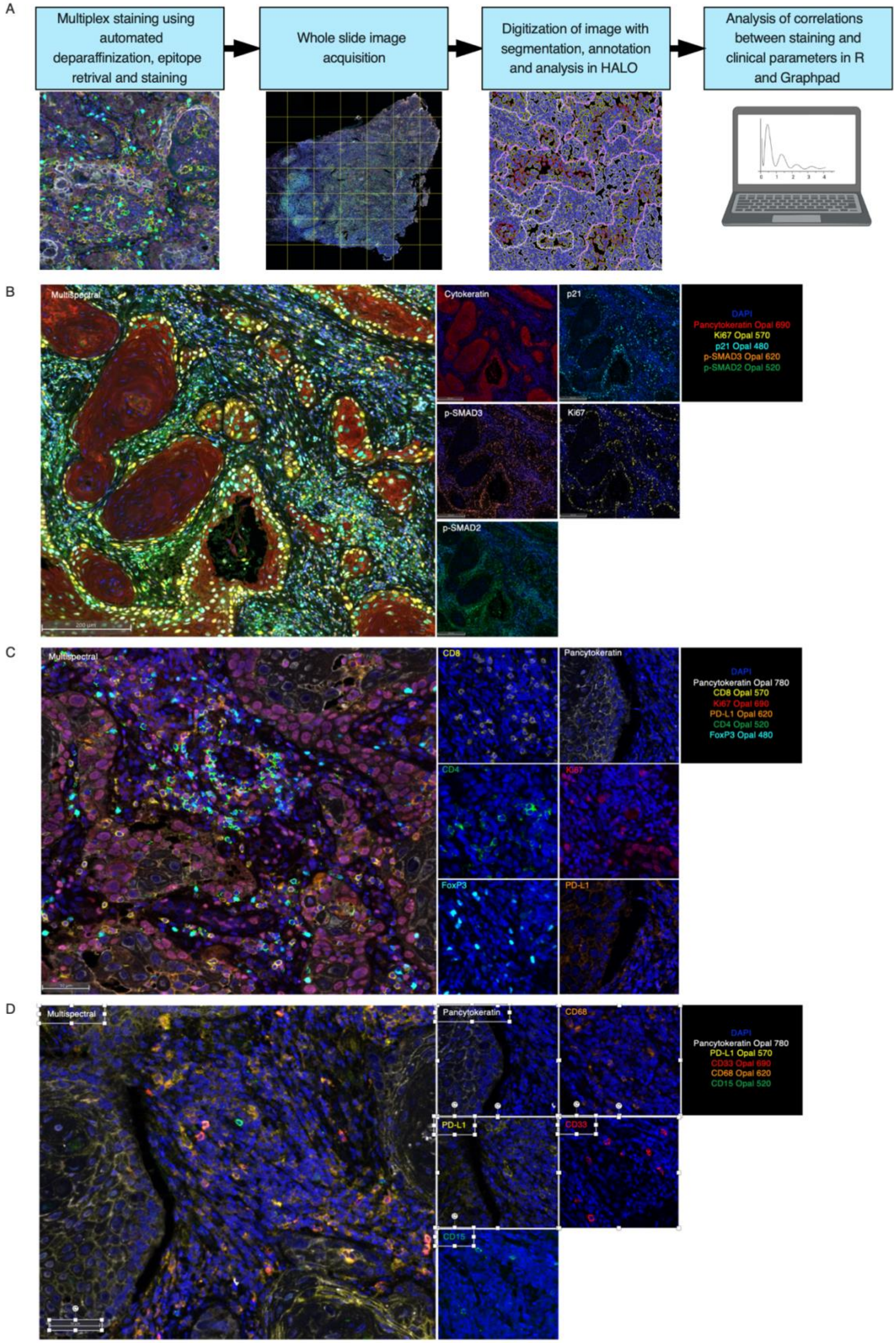
A
Multiplex staining using automated deparaffinization, epitope retrival and staining
Whole slide image acquisition
Digitization of image with segmentation, annotation and analysis in HALO
Analysis of correlations between staining and clinical parameters in R and Graphpad
B
Multispectral
Cytokeratin
p21
p-SMAD3
Ki67
p-SMAD2
DAPI
Pancytokeratin Opal 690
Ki67 Opal 570
p21 Opal 480
p-SMAD3 Opal 620
p-SMAD2 Opal 520
C
Multispectral
CD8
Pancytokeratin
CD4
Ki67
FoxP3
PD-L1
DAPI
Pancytokeratin Opal 780
CD8 Opal 570
Ki67 Opal 690
PD-L1 Opal 620
CD4 Opal 520
FoxP3 Opal 480
D
Multispectral
Pancytokeratin
CD68
PD-L1
CD33
CD15
DAPI
Pancytokeratin Opal 780
PD-L1 Opal 570
CD33 Opal 690
CD68 Opal 620
CD15 Opal 520

Supplemental Figure 4 – Workflow and examples of multispectral immunofluorescence

A, schema demonstrating the workflow of multispectral immunofluorescence staining and analysis.

B, representative multispectral and single-color photomicrographs of the TGF-β panel. Fluorescent colors corresponding to each parker are listed in the black box.

C, representative multispectral and single-color photomicrographs of the T cell panel. Fluorescent colors corresponding to each parker are listed in the black box.

D, representative multispectral and single-color photomicrographs of the myeloid cell panel. Fluorescent colors corresponding to each parker are listed in the black box.

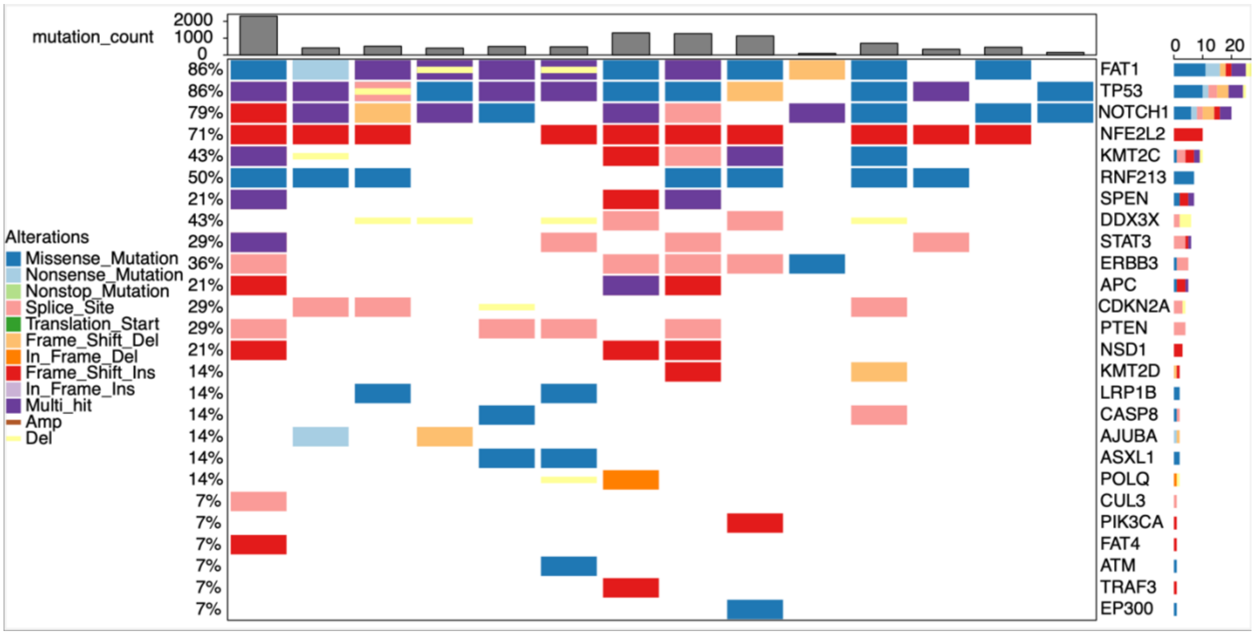

Supplemental Figure 5 – Oncoplot of frequently mutated genes

Heatmap (oncoplot) shows mutations within the 26 most frequently mutated genes in all 14 patients. The barplots above each heatmap show the total number of mutations within each sample.

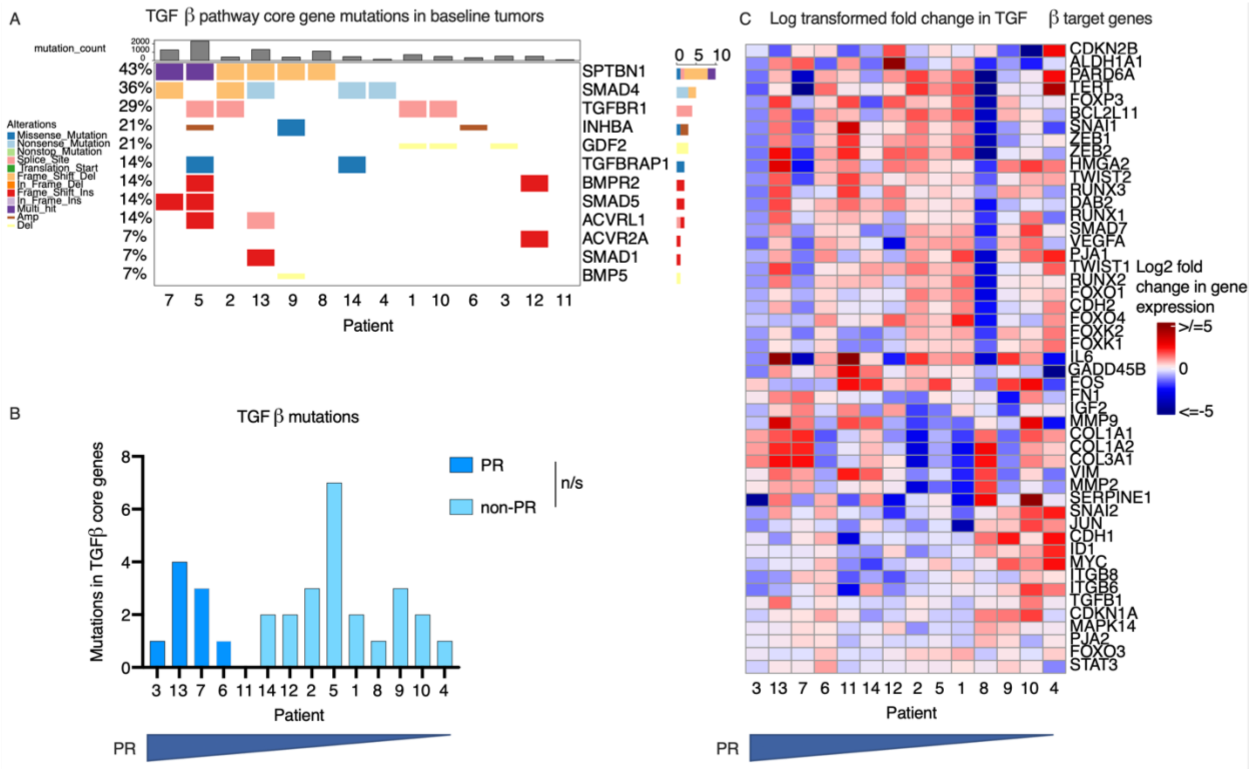

Supplemental Figure 6 – TGF-β pathway genomic and transcriptomic analysis

A, heatmap (oncoplot) of mutations identified within core TGF-β signaling genes in all 14 patients. The barplots above each heatmap show the total number of mutations within each sample.

B, bar plot shows the total number of mutated core TGF-β signaling genes for each patient, ranked from left to right in decreasing percentage tumor regression. Significance between patients that did or did not display a pathologic response was determined with a Mann-Whitney two-tailed test.

C, heatmap shows the Log2FC in expression of individual TGF-β target gene after treatment compared to before for each patient, ranked from left to right in decreasing percentage tumor regression.

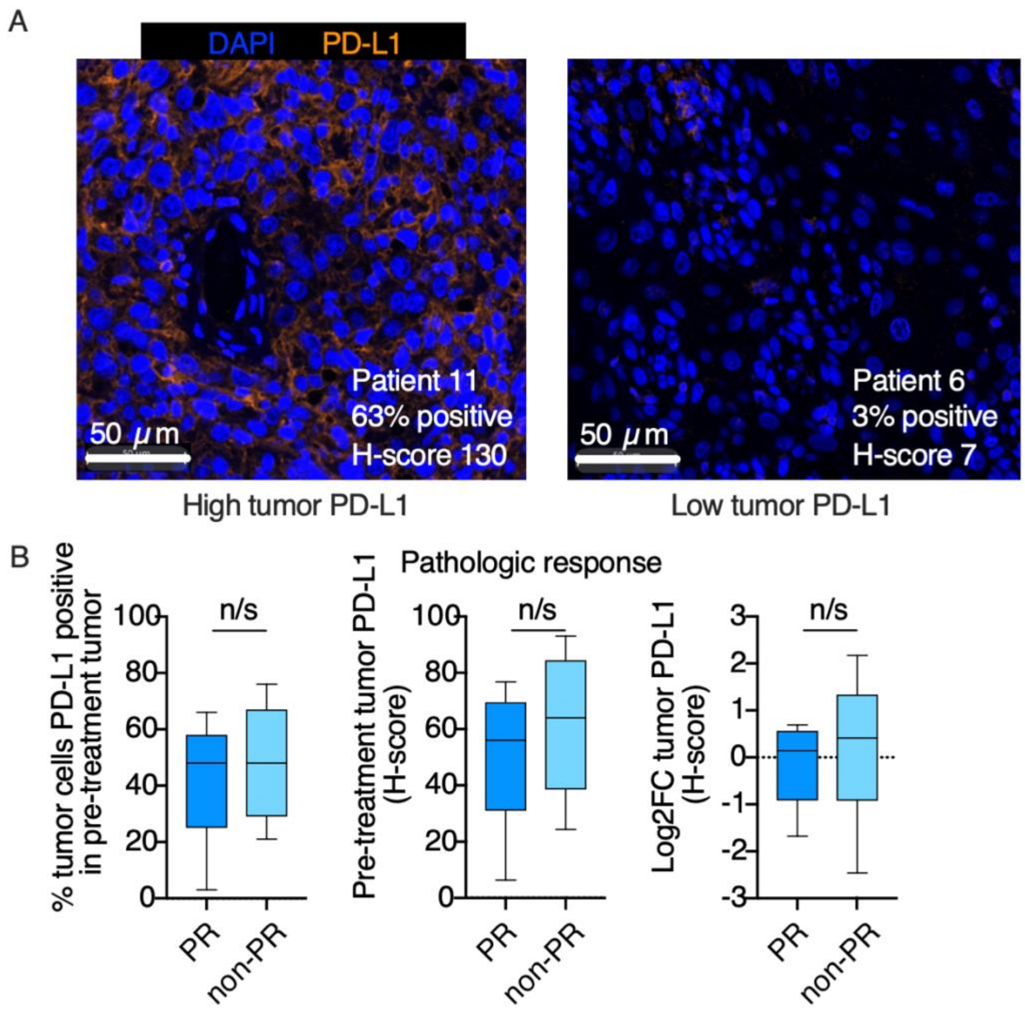

Supplemental Figure 7 – Tumor PD-L1 expression

A, representative photomicrographs of pre-treatment tumor PD-L1 expression measured by immunofluorescence. Patient 11 demonstrated high PD-L1 expression and patient 6 demonstrated low expression.

B, box and whisker plots show quantification of pre-treatment percentage positive PD-L1 tumor cells, pre-treatment tumor PD-L1 H-score, and Log2FC of tumor PD-L1 H-score after treatment compared to before for patients that did (n=5) or did not (n=9) demonstrate a pathologic response. Significance determined with a Mann-Whitney two-tailed test.

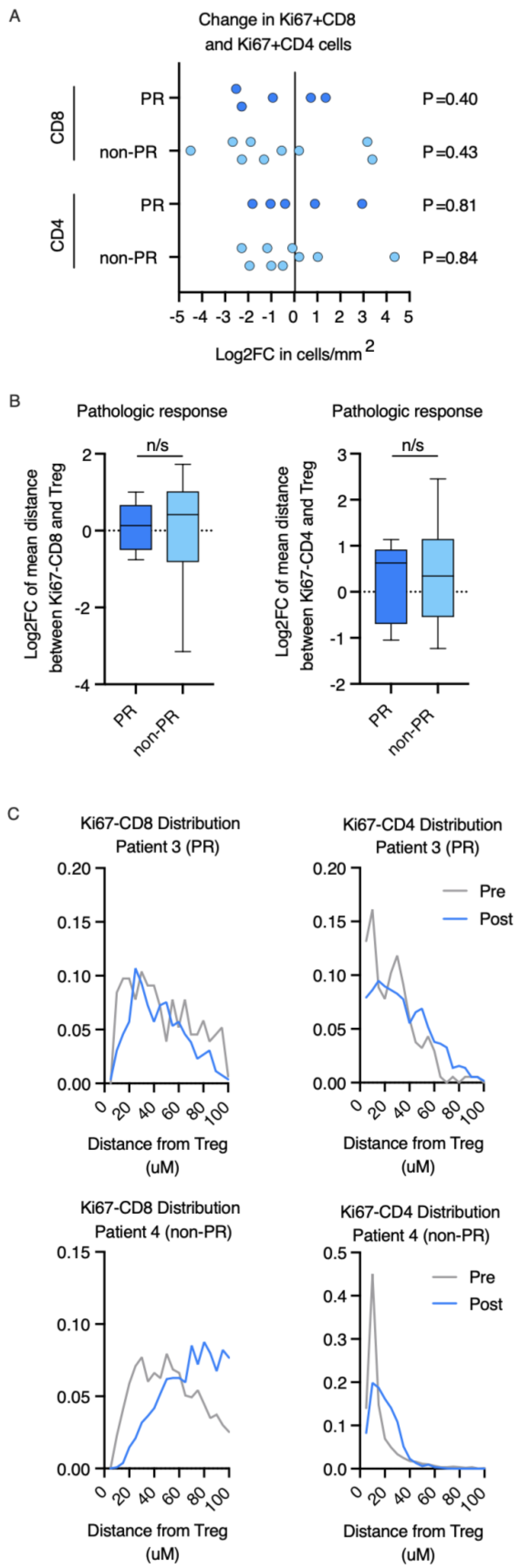
A
Change in Ki67+CD8
and Ki67+CD4 cells
CD8
CD4
PR
non-PR
P=0.40
P=0.43
P=0.81
P=0.84
-5 -4 -3 -2 -1 0 1 2 3 4 5
Log2FC in cells/mm 2
B
Pathologic response
n/s
Log2FC of mean distance
between Ki67-CD8 and Treg
Log2FC of mean distance
between Ki67-CD4 and Treg
PR
non-PR
C
Ki67-CD8 Distribution
Patient 3 (PR)
Ki67-CD4 Distribution
Patient 3 (PR)
Ki67-CD8 Distribution
Patient 4 (non-PR)
Ki67-CD4 Distribution
Patient 4 (non-PR)
Pre
Post
Distance from Treg
(uM)

Supplemental Figure 8 – Distribution of Ki67 negative T cells around Tregs

A, dot plot shows the log2 fold change (Log2FC) of whole slide Ki67+CD8 or Ki67+CD4 cell density (cells/mm^2) after treatment compared to before in patients that did (n=5) or did not (n=9) display a pathologic response. Significance determined with a one sample t test.

B, box and whiskers plots show the Log2FC of the mean distance between Ki67 negative CD8 or CD4 cells and Tregs after treatment compared to before in patients that did (n=5) or did not (n=9) display a pathologic response. Significance determined with a Mann-Whitney two-tailed test.

C, distribution plots show the probability that a Ki67 negative CD8 or CD4 cell will be a given distance from a Treg in pre-treatment (grey line) or post-treatment (blue line) tumors. Patient 3 is a representative example of a patient that developed a pathologic response; patient 4 did not develop a pathologic response.

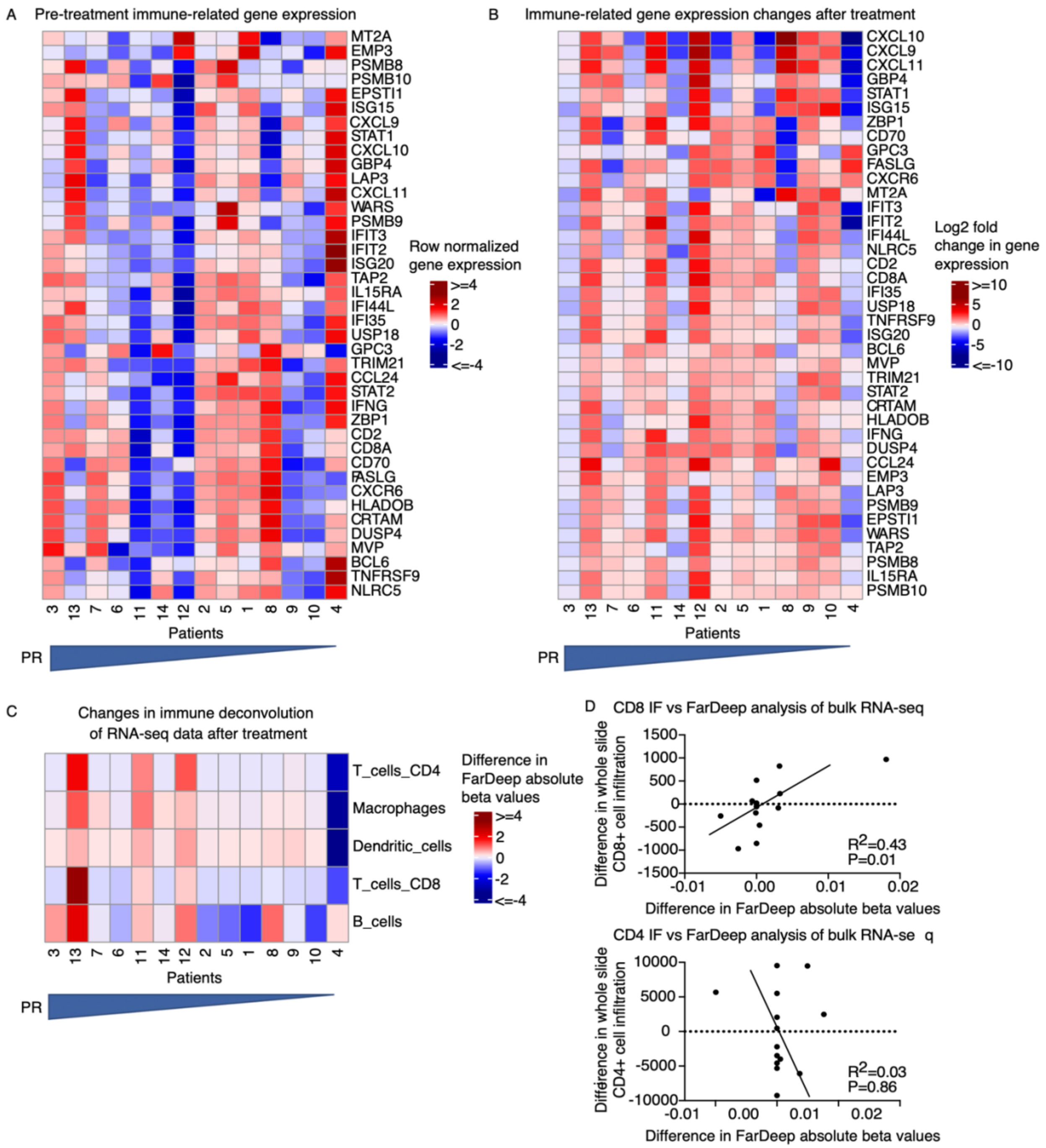

Supplemental Figure 9 – Transcriptomic immune profiling of tumors

A, heatmap of immune related gene expression in pre-treatment tumors from each patient, ranked from left to right in decreasing percentage tumor regression. Expression of each gene is row-normalized.

B, heatmap shows Log2FC in expression of immune related genes after treatment compared to before for each patient, ranked from left to right in decreasing percentage tumor regression.

C, heatmap shows Log2FC in absolute beta values determined by FarDeep analysis after treatment compared to before for each patient, ranked from left to right in decreasing percentage tumor regression.

D, dot plots show the correlation between the difference in whole slide CD8 (top) or CD4 (bottom) infiltration measured by MIF and difference in FarDeep estimation of CD8 or CD4 infiltration after treatment compared to before. Significance determined with a linear regression Goodness of fit test.

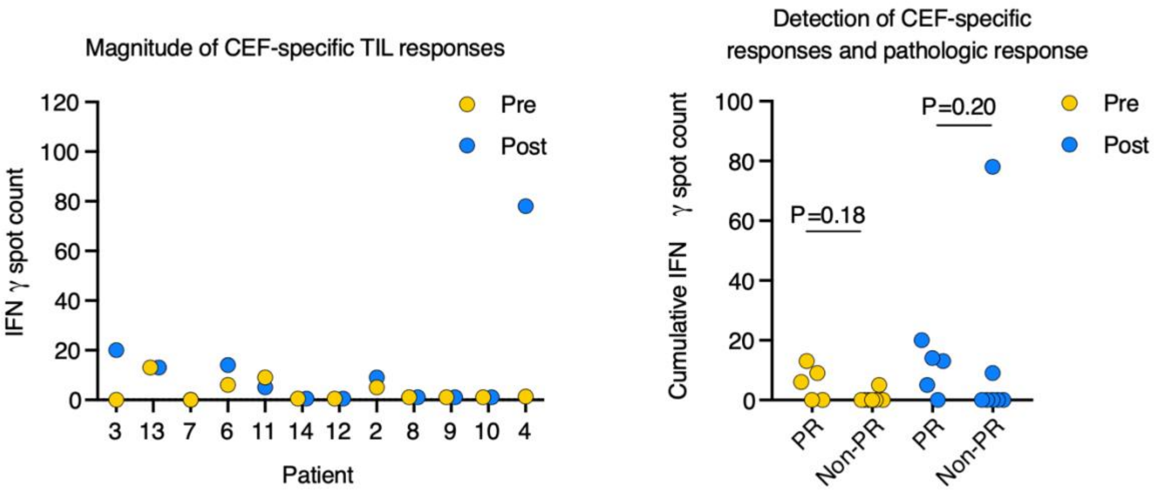

Supplemental Figure 10 – Viral-specific TIL responses

Dot plot on the left shows the number of viral (CEF)-specific cumulative IFNγ spots in ELISpot analysis of pre-treatment and post-treatment TIL for each patient, ranked from left to right in decreasing percentage tumor regression within the tumor bed. Dot plot on the right shows the same in patients that did (n=5) or did not (n=7) display a pathologic response. Significance determined with a Mann-Whitney two-tailed test.
