# Supplementary material for: Enhanced neoepitope-specific immunity following neoadjuvant PD-L1 and TGF-β blockade in HPV-unrelated head and neck cancer
Source: J Clin Invest. 2022 Sep 15;132(18):e161400. doi: 10.1172/JCI161400 (PMC9479764; doi:10.1172/JCI161400)
Supplement: Trial reporting checklists [file jci-132-161400-s126.pdf]

Abbreviated Title: Neoadjuvant IO for HNSCC

CC Protocol #: 20C0024 A

Version Date: May 7th, 2020

NCT Number: NCT04247282

Title: A Sequential Window of Opportunity Trial of Anti-PD-L1/TGF-β trap (M7824) Alone and in Combination with TriAd Vaccine, and N-803 for Resectable Head and Neck Squamous Cell Carcinoma not Associated with Human Papillomavirus Infection.

NCI Principal Investigator: Jason M. Redman, MD
Genitourinary Malignancies Branch (GMB)
Center for Cancer Research (CCR)
National Cancer Institute (NCI)
Building 10, Rm 13N240
10 Center Drive
Bethesda, MD 20892
Phone: 240-858-3305
E-mail: jason.redman@nih.gov

Investigational Agents:

Drug Name	M7824 (Bintrafusp alfa, MSB0011359C)	N-803 (ALT-803)	ETBX-011	ETBX-061	ETBX-051
			Collectively called TriAd vaccine		
Sponsor	CCR, NCI				
IND Number	019285				
Manufacturer	EMD Serono	NantCell	NantCell	NantCell	NantCell

Commercial Agents: None

PRÉCIS

Background:

- Approximately 50% of patients with advanced, non-HPV associated head and neck squamous cell carcinoma (HNSCC) will develop locoregional or distant relapse within two years of completing definitive standard-of-care treatment.
- Two ongoing clinical trials investigating neoadjuvant PD-1 blockade before surgical resection of HNSCC suggest that immunotherapy can both cytoreduce existing disease before surgery and reduce the risk of locoregional or distant disease relapse after surgery.
- Preliminary data from these studies suggest neoadjuvant treatments can be administered without delaying planned surgical intervention.
- Experiments conducted by the Laboratory of Tumor Immunology and Biology (LTIB) demonstrated synergistic activity with tumor-targeted adenoviral vaccine plus M7824 plus N-803 in humanized mice bearing human carcinomas and *in vitro* studies.
- M7824 is a bifunctional fusion protein consisting of an anti-programmed death ligand 1 (PD-L1) antibody and the extracellular domain of transforming growth factor beta (TGF-β) receptor type 2, a TGF-β trap.
- Adenoviral vaccines targeting known shared tumor antigens can generate antigen-specific T cells.
- N-803 is an IL-15/IL-15Rα super agonist complex that can enhance both NK cell and T cell anti-tumor activity via expansion and activation.
- Activity observed with neoadjuvant anti-PD-1 agents alone provides rationale for testing of M7824 alone and in combination with other immune-oncologic agents that have been shown to work in concert with M7824 in preclinical studies.
- Analysis of pre- and post-treatment tissues from HNSCC patients presents a unique opportunity to interrogate the effects the above treatment(s) on tumor.
- A dose escalation of N-803 in combination with a flat dose of M7824 was conducted at the National Cancer Institute. Thirteen patients have been treated with the combination. No DLTs were observed.

Objectives:

- Determine the rate of pathologic complete response (pCR) or clinical-to-pathological downstaging in patients with previously untreated intermediate/high risk, non-HPV associated , squamous cell carcinoma of the head and neck (T1-T4, N0-N3, M0 stage II, III or IV) who receive any of the three proposed treatments: M7824 alone, M7824 plus TriAd vaccine, or M7824 plus TriAd vaccine plus N803 prior to definite surgery.

Eligibility:

- Patients must have histologically or cytologically confirmed, previously untreated intermediate/high risk, p16-negative (if oropharyngeal), squamous cell carcinoma of the head and neck (T1-T4, N0-N3, M0 stage II, III or IV)
- Men or Women; Age ≥18 years
- ECOG performance status ≤ 1

Design:

- This protocol is a sequential window of opportunity trial of Anti-PD-L1/TGF-β trap (M7824) alone and in combination with TriAd Vaccine and N-803 for non-HPV associated resectable Head and Neck Squamous Cell Carcinoma (HNSCC).
- Patients will be referred to the NIH for this immunotherapy treatment from surrounding academic medical centers and private physicians.
- Upon referral to the NIH, patients will be rapidly screened, and enrolled on the protocol, if appropriate.
- This trial will enroll patients in three arms sequentially to permit safety evaluation before adding the next agent.
- In the first arm of 12 patients, M7824 (1200 mg) will be administered intravenously on day 1 and 15.
- If no safety concerns, accrual will proceed to the 2nd arm, and 12 patients will enroll with M7824 (1200mg; intravenous) and TriAd vaccine (5 x 10^{11} VP; subcutaneous) treatment on day 1 and 15.
- If no safety concerns, accrual will proceed to the 3rd arm, and 12 patients will enroll with M7824 ((1200mg; intravenous) plus TriAd vaccine (5 x 10^{11} VP; subcutaneous injection) on day 1 and 15 plus N-803 (15mcg/kg, subcutaneously) treatment on day 1.
- After obtaining pre-treatment biopsies, imaging and blood collection, patients will receive the neoadjuvant immunotherapy at the NIH Clinical Center.
- Patients will then be sent back to their referring providers for their definitive standard of care surgery and adjuvant therapy as indicated based upon pathologic analysis of the surgical specimen. NCI investigators will have no role in directing the ensuing standard of care surgeries performed at outside institutions.
- For consistency in pathologic analysis of resection specimens, tissue blocks and/or slides will be obtained from outside institutions and be reviewed by the NCI Laboratory of Pathology.
- It is expected that up to 20 patients may enroll in one year. Thus, with 3 arms of 12 patients apiece, up to 36 evaluable patients may enroll. Accrual is expected to be completed within 2 years.

TABLE OF CONTENTS

PRÉCIS 2
TABLE OF CONTENTS 4
STATEMENT OF COMPLIANCE 7
1. INTRODUCTION 8
1.1 Study Objectives 8
1.2 Background and Rationale 8
2 ELIGIBILITY ASSESSMENT AND ENROLLMENT 19
2.1 Eligibility Criteria 19
2.2 Screening Evaluation 21
2.3 Registration Procedures 23
2.4 Baseline Evaluation 23
3 STUDY IMPLEMENTATION 24
3.1 Study Design 24
3.2 Drug Administration 27
3.3 Dose Modifications 28
3.4 Study Calendar 30
3.5 Cost and Compensation 35
3.6 Criteria for Removal from Protocol Therapy and Off Study Criteria 35
4 CONCOMITANT MEDICATIONS/MEASURES 36
4.1 General guidance 36
4.2 N-803 36
5 BIOSPECIMEN COLLECTION 38
5.1 Correlative Studies for Research/Pharmacokinetic Studies 38
5.2 Sample Storage, Tracking and Disposition 42
5.3 Samples for Genetic/Genomic Analysis 45
6 DATA COLLECTION AND EVALUATION 46
6.1 Data Collection 46
6.2 Data Sharing Plans 47
6.3 Response Criteria 48
6.4 Toxicity Criteria 53
7 NIH REPORTING REQUIREMENTS/DATA SAFETY MONITORING PLAN 54

7.1 Definitions ... 54
7.2 OHSRP Office of Compliance and Training / IC Specific IRB Reporting ... 54
7.3 NCI Clinical Director Reporting ... 54
7.4 Institutional Biosafety Committee (IBC) Reporting Criteria ... 54
7.5 NIH Required Data and Safety Monitoring Plan ... 55
8 Sponsor Protocol/ Safety Reporting ... 56
8.1 Definitions ... 56
8.2 Assessment of Safety Events ... 57
8.3 Reporting of Serious Adverse Events ... 58
8.4 Safety Reporting Criteria to the Pharmaceutical Collaborators ... 58
8.5 Reporting Pregnancy ... 58
8.6 Regulatory Reporting for Studies Conducted Under CCR-Sponsored IND ... 59
9 CLINICAL MONITORING ... 59
10 STATISTICAL CONSIDERATIONS ... 60
10.1 Statistical Hypothesis ... 60
10.2 Sample Size Determination ... 60
10.3 Populations for analysis ... 61
10.4 Statistical analysis ... 61
11 COLLABORATIVE AGREEMENTS ... 63
11.1 Cooperative Research and Development Agreement (CRADA) ... 63
12 HUMAN SUBJECTS PROTECTIONS ... 63
12.1 Rationale For Subject Selection ... 63
12.2 Participation of Children ... 63
12.3 Participation of Subjects Unable to Give Consent ... 63
12.4 RISK/Benefits Assessment ... 64
12.5 Consent and Assent Process and Documentation ... 66
13 REGULATORY AND OPERATIONAL CONSIDERATIONS ... 66
13.1 Study Discontinuation and Closure ... 66
13.2 Quality Assurance and Quality Control ... 67
13.3 Conflict of Interest Policy ... 67
14 PHARMACEUTICAL INFORMATION ... 68
14.1 ETBX 011, 051, 061 ... 68

14.2 M7824 71
14.3 N-803 (ALT-803)........ 74
15 REFERENCES 76
16 Appendix A: -Performance Status Criteria........ 79

STATEMENT OF COMPLIANCE

The trial will be carried out in accordance with International Conference on Harmonisation Good Clinical Practice (ICH GCP) and the following:

- United States (US) Code of Federal Regulations (CFR) applicable to clinical studies (45 CFR Part 46, 21 CFR Part 50, 21 CFR Part 56, 21 CFR Part 312, and/or 21 CFR Part 812)

National Institutes of Health (NIH)-funded investigators and clinical trial site staff who are responsible for the conduct, management, or oversight of NIH-funded clinical trials have completed Human Subjects Protection and ICH GCP Training.

The protocol, informed consent form(s), recruitment materials, and all participant materials will be submitted to the Institutional Review Board (IRB) for review and approval. Approval of both the protocol and the consent form must be obtained before any participant is enrolled. Any amendment to the protocol will require review and approval by the IRB before the changes are implemented to the study. In addition, all changes to the consent form will be IRB-approved; a determination will be made regarding whether a new consent needs to be obtained from participants who provided consent, using a previously approved consent form.

1. INTRODUCTION

1.1 STUDY OBJECTIVES

1.1.1 Primary Objective

Determine the rate of pathologic complete response (pCR) or clinical-to-pathological downstaging in patients with previously untreated intermediate/high risk, non HPV-associated squamous cell carcinoma of the head and neck (T1-T4, N0-N3, M0 stage II, III or IV) who receive any of the three proposed treatments: M7824 alone, M7824 plus TriAd vaccine, or M7824 plus TriAd vaccine plus N803.

1.1.2 Secondary Objectives

- Estimate the rate of grade 3 or 4 immune related adverse events (irAEs)
- Determine the rate of treatment-related adverse events (AE) causing a delay of 4 weeks or more beyond planned surgery
- Estimate the response rate of the primary disease by CT imaging (RECIST)
- Estimate the one- and two-year recurrence free survival
- One- and two-year overall survival

1.1.3 Exploratory Objectives

- Define immune infiltrate and localization using multiplex immunofluorescence
- Define T cell clonality using Deep TCR-seq
- Determine tumor infiltrating lymphocyte (TIL) responses to shared antigens and antigens predicted from exome and RNA sequencing
- Assess antigen specific responses using peripheral T cell isolation
- Multiparameter characterization of PBMC by flow cytometry
- Assessment of changes in serum cytokines
- Assessment of changes in TGFβ signaling in tumor, normal mucosa and skin

1.2 BACKGROUND AND RATIONALE

1.2.1 Poor outcomes in patients with advanced stage, head and neck cancer not associated with HPV infection

Definitive standard-of-care treatment for patients with locally-advanced or locoregionally advanced (stage II, III or IV) non-HPV associated head and neck squamous cell carcinoma (HNSCC) of the oral cavity, oropharynx, hypopharynx and larynx includes multi-modality therapy consisting of concurrent chemoradiation (CRT) or surgery followed by adjuvant radiotherapy with or without chemotherapy. In contrast to patients with human papillomavirus (HPV)-positive oropharyngeal cancer, patients with newly diagnosed advanced stage HPV-negative HNSCC have poor outcomes after definitive standard-of-care treatment [1]. Negative p16 status is a surrogate marker for an HPV-negative tumor (p16-positive tumors are HPV-positive) in tumors originating in the oropharynx. Involvement of HPV and therefore p16 status, have not been demonstrated to be biologically relevant in head and neck cancers originating in locations other than the oropharynx (e.g. oral cavity, larynx, hypopharynx or nasopharynx) [2]. Therefore, these tumors are considered

to be 'non-HPV associated.' Despite maximum treatment, locoregional recurrence (LR) or distant metastasis (DM) will develop in 35% of patients with non-HPV-associated HNSCC in the first one year (Figure 1). Five-year overall survival (OS) is less than 50% [3]. Additionally, standard-of-care treatments for advanced stage HNSCC leave cured patients with devastating functional impairments [4]. More efficacious and less toxic treatments are needed for advanced stage, non-HPV associated, HNSCC.

Figure 1: Disease-free survival in patients with advanced p16-negative HNSCC treated with radiotherapy (RT) or RT plus chemotherapy.

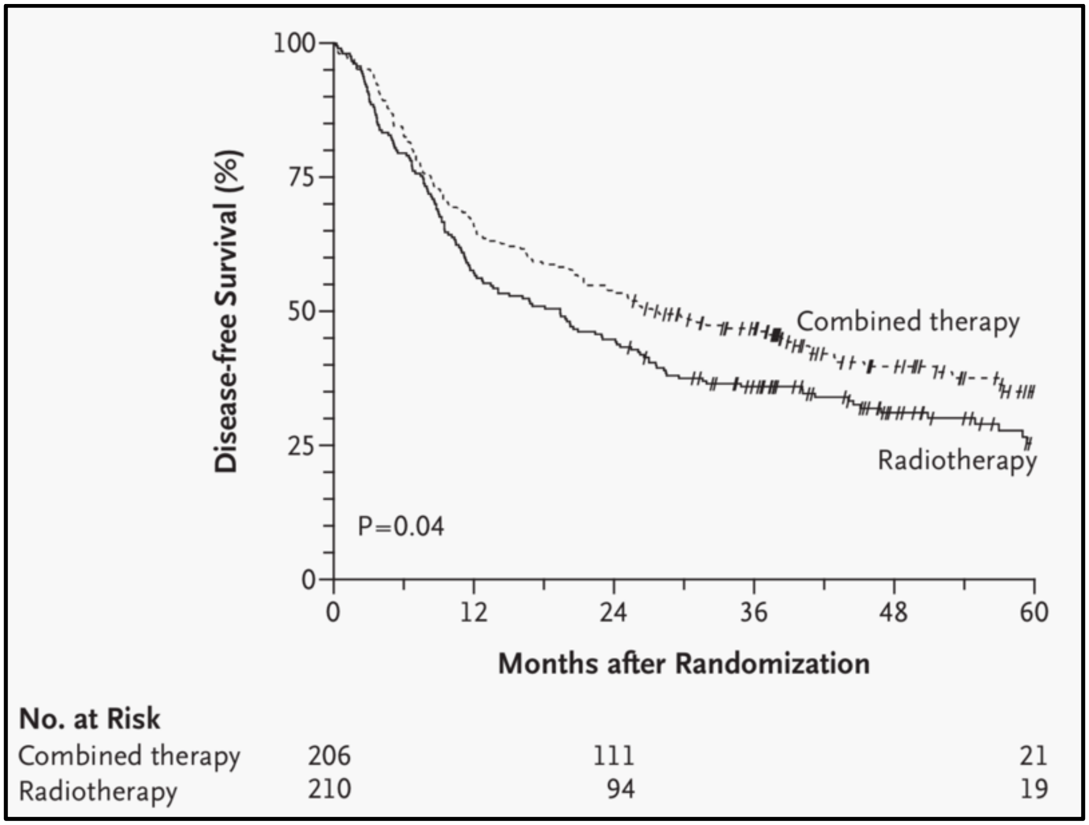

1.2.2 Immunotherapy has clinical activity in patients with HNSCC

HNSCC has features that make it ideal for immunotherapy. The majority of HNSCC specimens demonstrate evidence of T cell infiltration and PD-L1 expression indicative of an underlying immune response [5]. Additionally, HNSCCs harbor many genomic alterations that may give rise to an array of T cell neoantigens [6, 7]. Based on safety and improved overall survival reported in two clinical trials, both pembrolizumab and nivolumab, two programmed death receptor-1 (PD-1) monoclonal antibodies (mAbs), are FDA-approved for patients with recurrent/metastatic HNSCC that have failed systemic platinum-based chemotherapy [5, 8]. These studies demonstrate a manageable safety profile but objective responses in only 15-20% of patients following PD-1 blockade. Optimal timing of immune checkpoint blockade, or combination immunotherapy targeting resistance mechanisms, may enhance response rates and lead to improved patient survival.

1.2.3 Neoadjuvant immunotherapy may have increased clinical activity in HNSCC

Treatment with immunotherapy before definitive extirpative surgery (neoadjuvant treatment) may optimally activate anti-tumor immunity. The use of up-front neoadjuvant or induction immunotherapy before standard-of-care treatment for HNSCC is being actively studied in at least nine clinical trials worldwide. Pre-clinical work from our laboratory demonstrates that PD-1 mAb treatment before surgical removal of tumor in mice leads to greater induction of neoantigen-specific memory T cells and anti-tumor immunity compared to PD-1 mAb treatment after surgical removal of tumor in multiple models of head and neck cancer (Figure 2). This data suggests that immunotherapy administered when tumor antigen is present followed by removal of tumor may lead to enhanced immune responses. Immunotherapy in the up-front treatment setting before surgical resection may increase the magnitude of response or increase the percentage of patients that respond. Additionally, surgical specimens offer an opportunity to assess for alterations in clinical-to-pathologic staging and evidence of major pathologic response as trial outcome measures [9].

Ongoing clinical trials at the Dana Farber Cancer Institute and University of Pittsburgh are exploring neoadjuvant pembrolizumab or nivolumab before surgery for advanced oral cavity cancer have set the precedent for this treatment schema. Preliminary results from these studies have demonstrated 1) no grade 3/4 adverse events, 2) no delays in pre-determined time to surgery with surgical treatment occurring two to three weeks following the final dose of immunotherapy, 3) improved disease-specific survival at the one-year mark, and 4) evidence of clinical activity with major pathologic responses in surgery specimens and clinical-to-pathologic downstaging [10] (NCT02296684), [11] (NCT02488759). Thus, neoadjuvant single agent immune checkpoint inhibitor immunotherapy before definitive surgical resection in patients with advanced HNSCC appears be safe and have clinical activity.

Figure 2: Neoantigen-induced IFN production by tumor draining lymph nodes harvested 40 days after resection of oral carcinomas (left) in a syngeneic model of carcinogen-induced oral cavity squamous cell carcinoma called MOC1 (mouse oral cancer 1). Peripheral T cells were assessed for neoantigen: MHC class I tetramer positivity 40 days after resection of oral carcinomas (right).

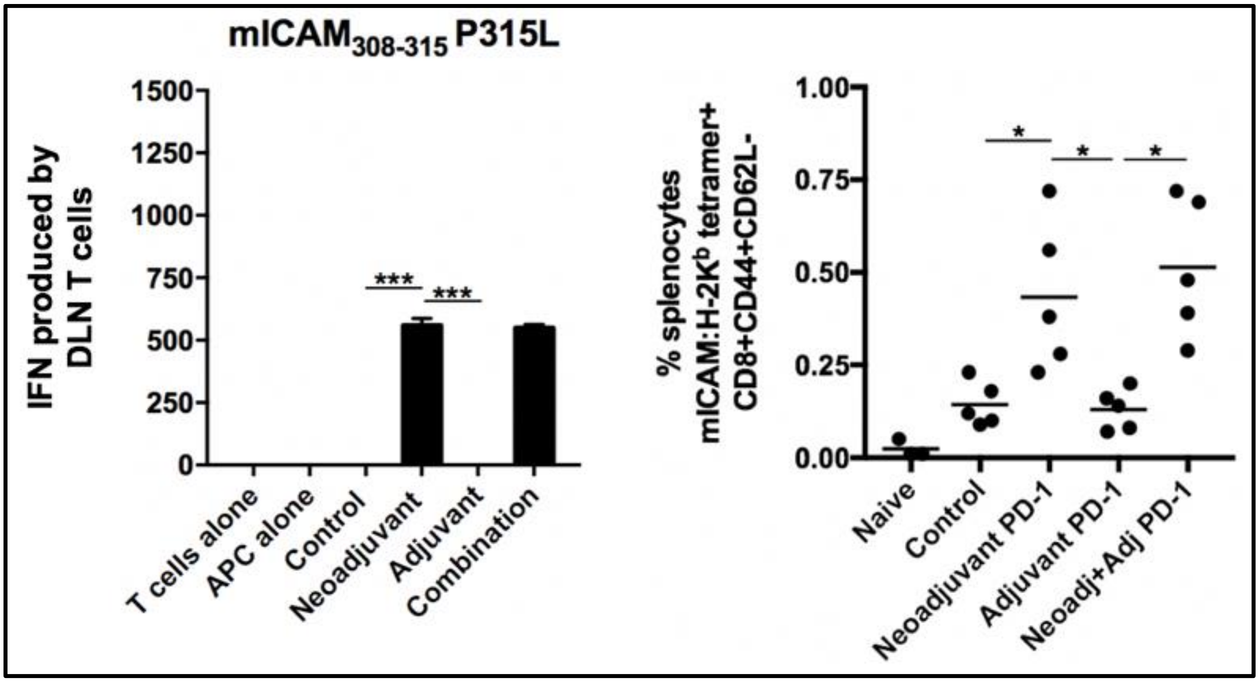

1.2.4 Rationale for combination of I/O agents

Preclinical studies are now revealing that an effective immuno-oncology strategy is the use of multiple immune-mediating agents, each targeting different components of the immune system. These include: (a) induction of an immune response via vaccination directed against tumor-associated antigens, (b) potentiation of the systemic immune response by the use of immunocytokines (such as the N-803 [also known as ALT-803], an IL-15 super agonist fusion protein) to enhance both NK and $CD8^+$ T-cell responses, and (c) reduction of immunosuppressive entities in the tumor microenvironment (TME) by the use of anti-PD1/PDL1 mAb checkpoint inhibitors, and/or reduction of immunosuppressive cytokines such as TGF-β with the use of a bifunctional anti-PDL1/TGF-β2 "Trap" designated M7824. The LTIB, in collaboration with the Genitourinary Malignancies Branch (GMB), has developed and/or co-developed these agents via a series of cooperative research and development agreements (CRADAs). Each has been interrogated in a series of preclinical studies and in Phase I/II clinical studies in the CCR as well as by other investigators. The preclinical studies and early clinical studies detailed below are demonstrating the relative lack of additional toxicities with enhanced clinical benefit employing combinations of these agents. Furthermore, preclinical studies are now demonstrating that the combined use of agents from each of the immune-mediating categories described above elicits the most immune-mediated anti-tumor activities.

Figure 3: Combined effect of Ad-Twist vaccine and M7824 in EMT6 carcinoma model

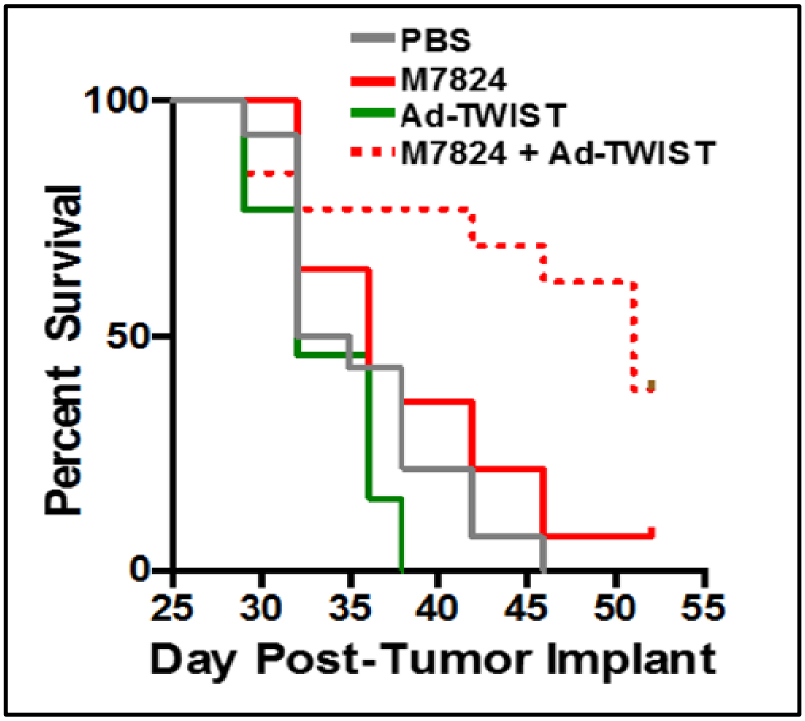

As previous shown in studies published by investigators within the LTIB [12], M7824 combined with tumor-targeted vaccine enhances antitumor activity in metastatic murine cancer models (Figure 3). Additionally, M7824 can mediate ADCC and the addition of N-803 to M7824 further enhances this ADCC activity. [13] (Figure 4).

Figure 4: ALT-803 enhances the NK-mediated lysis of avelumab and M7824 model

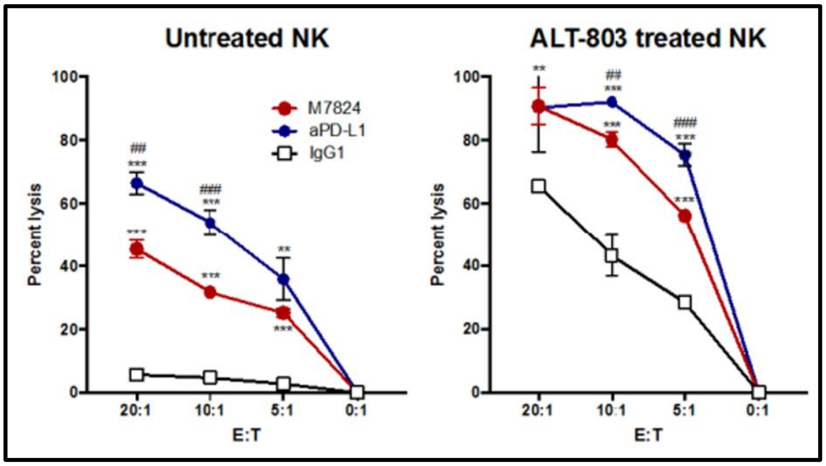

Employing NSG-β2m−/− mice bearing human breast MDA-MB-231 tumors and reconstituted with human PBMC, the combination of vaccine, N-803, and M7824 resulted in the greatest level of anti-tumor activity compared to the use of individual agents (Figure 5).

Figure 5: Multimodal therapy in mice reconstituted with PBMCs from patients

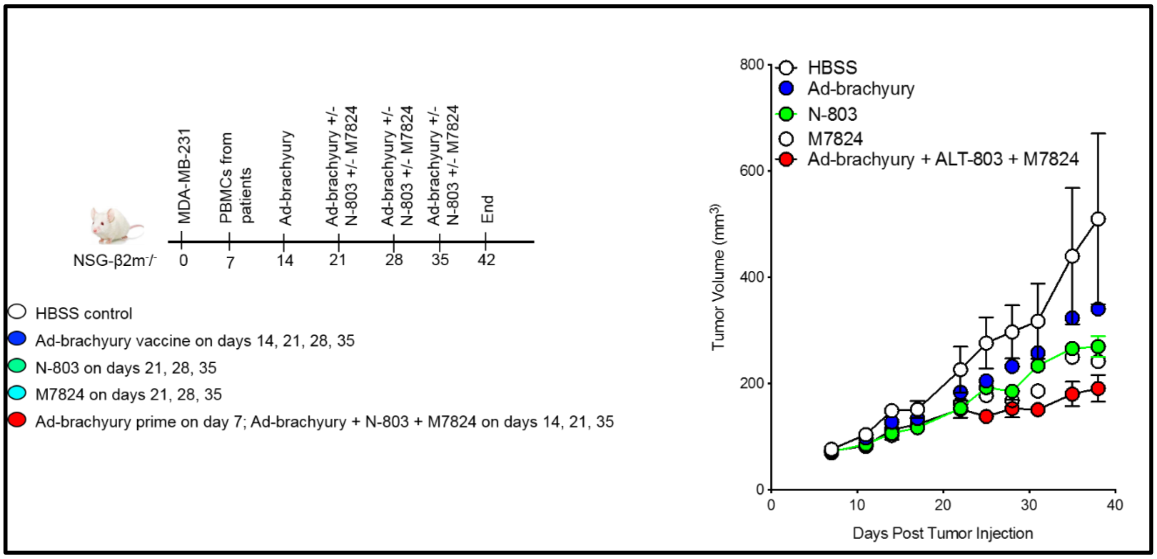

1.2.5 M7824

TGFβ is an immunosuppressive cytokine highly expressed in HNSCC by tumor, stromal and infiltrating immune cells and is a mechanism of resistance to immune checkpoint inhibitor immunotherapy[14, 15]. M7824 is a bifunctional fusion protein combining anti-PD-L1 mAb and TGFβ receptor type 2, serving as a TGFβ trap (Figure 6). By dually blocking PD-L1 and neutralizing TGFβ, M7824 treatment demonstrates robust anti-tumor immune activation in multiple pre-clinical models of cancer [16]. In a dose-escalation phase 1 study performed at the NIH Clinical Center, M7824 demonstrated a manageable safety profile and induced durable anti-tumor responses, as well as reduction in circulating TGFβ (Figure 7) [17]. Four of nineteen patients (21%) experienced a grade 3 or 4 adverse event; these included one case of grade 2 bullous pemphigoid, one case of grade 3 asymptomatic lipase increase, one case of grade 2 colitis, and once case of grade 3 hypokalemia. Only grade 2 colitis occurred within 30 days of initiating M7824 treatment.

Figure 6: M7824.

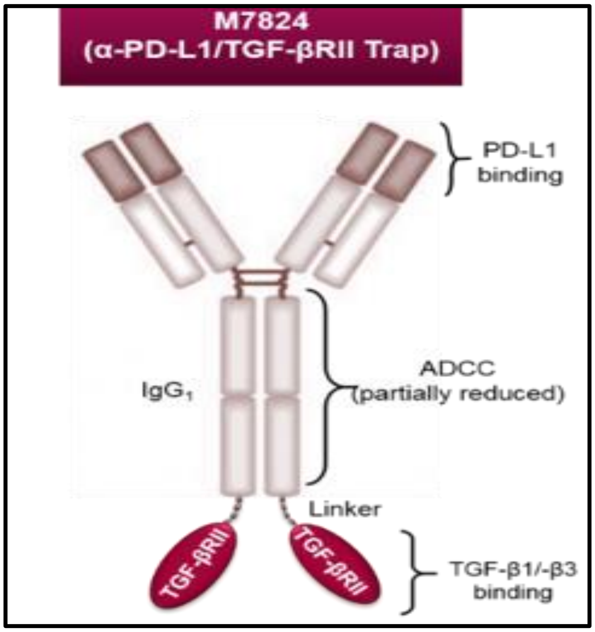

Approximately one-third of patients with advanced non-HPV associated HNSCC harbor tumors without evidence of T cell infiltration [5], suggesting these tumors may lack T cell antigens or have antigen processing or presentation defects. NK cells recognize tumors cells through antigen-independent mechanisms and may play a role in anti-tumor immunity in these cancers. In addition to activating T cells, M7824 can induce NK cell anti-tumor activity through antibody-dependent cell-mediated cytotoxicity (ADCC) and the in vivo anti-tumor activity of M7824 is dependent upon both T cells and NK cells [12, 16]. Thus, M7824 dually activates T and NK cell activity along with local neutralization of immunosuppressive TGFβ to maximally activate anti-tumor immunity.

Figure 7: Spider plot of clinical responses and mean serum TGFβ concentration in patients with recurrent/metastatic cancer receiving M7824.

1.2.6 N-803

N-803 is an IL-15 superagonist complex that expands the numbers and enhances the activity of effector T and NK cells, leading to significant in vivo anti-tumor activity when combined with immune checkpoint blockade in multiple pre-clinical solid tumor models [18]. As discussed above, M7824 enhances ADCC-mediated NK cell cytotoxicity [12]. Specifically, experiments conducted in the LTIB show that N-803 and M7824 work synergistically to enhance NK cell-mediated ADCC (Figure 8A) [19]. The combination of N-803 and immune checkpoint blockade (nivolumab) has been used clinically with a manageable safety profile and evidence of clinical activity [20]. No patient receiving this combination treatment experienced a grade 4 adverse event. Grade 3 adverse

events occurred in ≤10% of patients and included fever, fatigue, dizziness and alterations in clinical laboratory assessments .

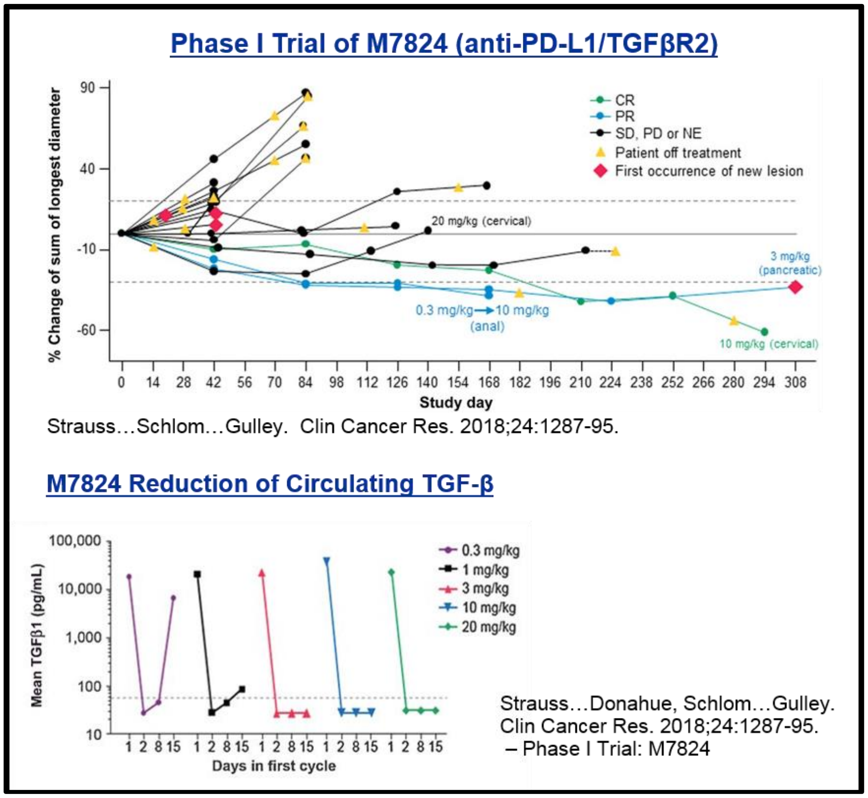

1.2.7 Clinical Safety and Experience with N-803 and anti-PD-1/L1 blockade at the NCI

The combination of N-803 and immune checkpoint blockade (nivolumab) has been used clinically with a manageable safety profile and evidence of clinical activity [20]. No patient receiving this combination treatment experienced a grade 4 adverse event. Grade 3 adverse events occurred in ≤10% of patients and included fever, fatigue, dizziness and alterations in clinical laboratory assessments. Furthermore, investigators at the NIH Clinical center have experience with administration of combination M7824 and N-803. As of January 2019, 13 solid tumor cancer patients have been treated with the M7824 1,200 mg IV every 2 weeks + N-803 (10-15 mcg/kg) SC every 2 weeks combination at the NCI. Four patients received a N-803 dosing of 10 mcg/kg every 2 weeks and 9 patients received N-803 dosing of 15 m cg/kg. No dose limiting, or unexpected toxicities attributed to the combination were observed. One patient with microsatellite stable, metastatic rectal cancer achieved a dramatic drop in CEA and improvement of cough attributed to lung metastasis after 3 treatments of M7824 and N-803. Imaging showed a partial response by RECIST version 1.1 criteria. (Figure 8B). Of note, this patient had received CEA, MUC1, and brachyury targeted adenoviral-based vaccine before enrolling on the M7824 + N-803 study.

Figure 8: A) Carcinoembryonic antigen decrease in a microsatellite stable colorectal cancer patient, who had previously received TriAd vaccine, while on M7824 and N-803.B) Corresponding decrease in lung metastasis in the same patient.

A

B

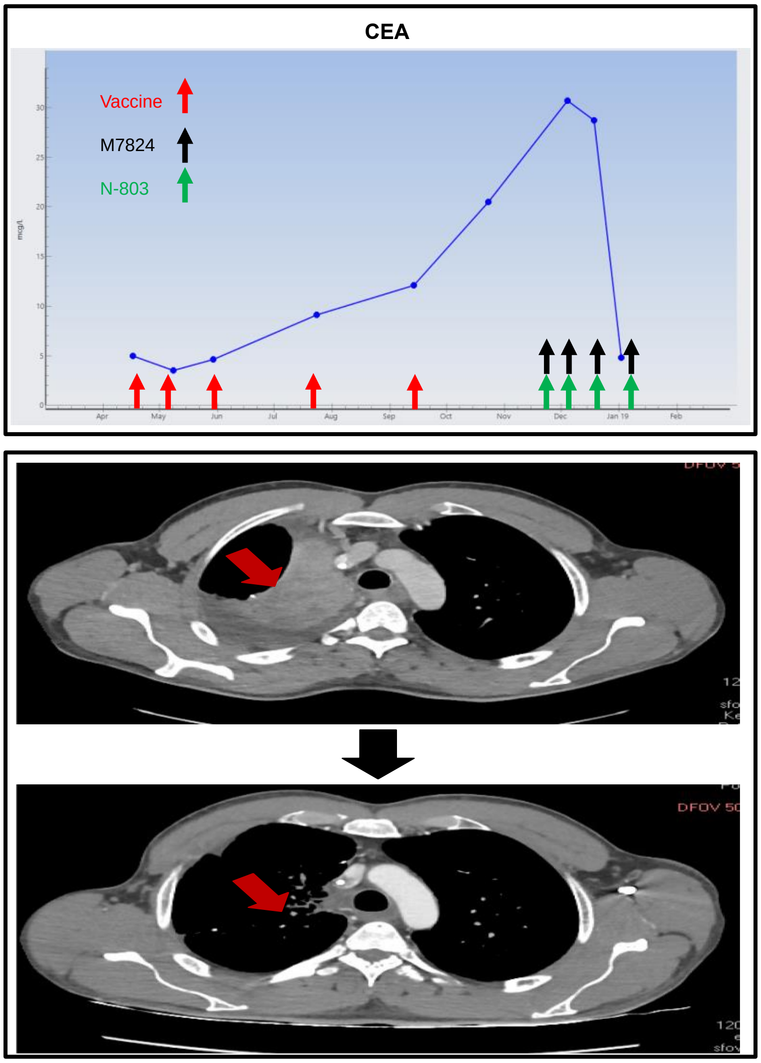

Pharmacodynamic data from Nant suggest optimal T cell and NK cell is achieved with 15 mcg/kg dosing every 3 weeks. Therefore, a one-time dose of 15 mcg/kg N-803 on day 1 of treatment will be used in this study.

1.2.8 Therapeutic brachyury, MUC1 and CEA adenovirus vaccines (TriAd vaccine)

The ability of adenoviral vaccination platforms to induce antigen-specific immune responses in human cancers is well studied. While vaccination of individual HNSCC cancer patients against predicted neoantigens has the potential to lead to tumor-specific immunity, the processes involved in developing personalized vaccines is cumbersome and costly.

Vaccination against shared antigens commonly expressed in HNSCCs allows for the use of a standardized vaccine platform, and pre-clinical data suggests that "spreading" of T cell responses against neoantigens can be induced following vaccination against tumor-associated antigens (unpublished data, Hamilton laboratory). Query of expression data from the HNSCC cohort of The Cancer Genome Atlas demonstrates that brachyury, MUC1 and CEA are commonly expressed in HNSCCs and represent rational shared antigen targets. (Figure 9).

Figure 9: Gene expression of brachyury, MUC1 and CEA from the HNSCC cohort of TCGA.

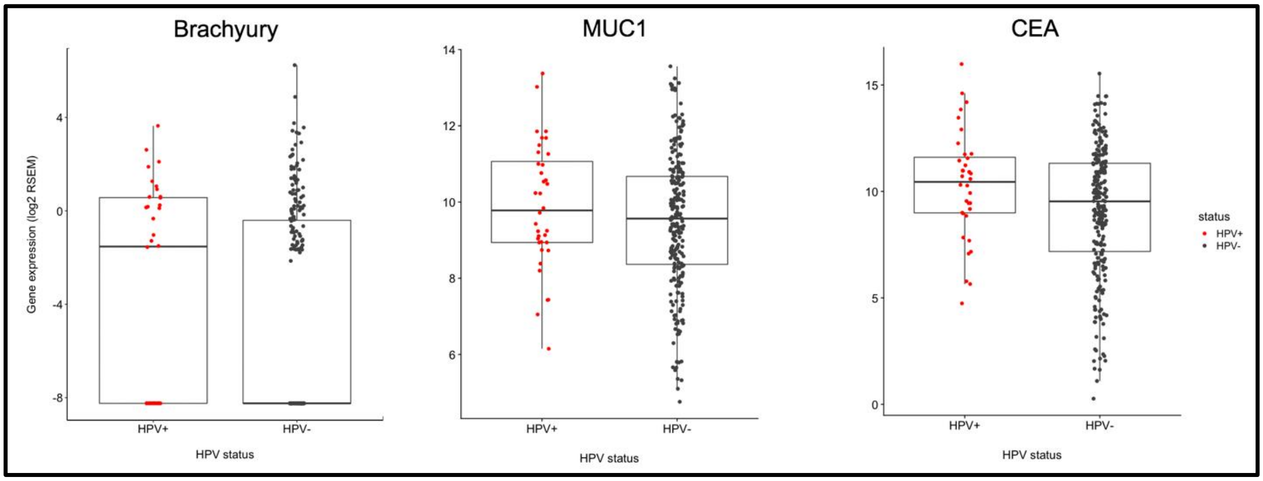

1.2.9 Clinical Experience with brachyury, MUC1 and CEA adenovirus vaccines at the NCI

Administration of adenoviral vaccines encoding for these tumor-associated antigens has shown a manageable toxicity profile and ability to induce antigen specific T cell response in phase I trial conducted by the GMB/LTIB at the NCI. The combination of ETBX-011 (CEA-targeted), ETBX -051 (brachyury-targeted) and ETBX-061 (MUC1-targeted) has been tested in ten subjects. No DLTs were observed. Common TRAEs were fever and injection site reactions, all grade 1 or 2. Preliminary immune response data from this trial is shown in Table 1.

Table 1: Immunogenicity of TriAd vaccine (ETBX-011 and ETBX-061): Development of MUC-1, CEA and Brachyury specific T cell responses by intracellular cytokine staining.

			MUC-1								CEA								Brachyury							
Patient #	Cancer Type	Post (vs Pre) # of vaccines	CD4 CD107a	CD4 IFNg	CD4 IL-2	CD4 TNF	CD8 CD107a	CD8 IFNg	CD8 IL-2	CD8 TNF	CD4 CD107a	CD4 IFNg	CD4 IL-2	CD4 TNF	CD8 CD107a	CD8 IFNg	CD8 IL-2	CD8 TNF	CD4 CD107a	CD4 IFNg	CD4 IL-2	CD4 TNF	CD8 CD107a	CD8 IFNg	CD8 IL-2	CD8 TNF
3	Liver Ca	1	0	185	0	0	0	543	3	0	0	0	0	0	0	0	43	0	0	0	0	44	0	362	0	0
		2	0	0	0	83	0	0	0	0	0	0	93	0	0	872	0	0	0	0	0	43	0	0	0	0
		3	97	7331	3866	12531	133	425	49	2609	0	0	0	0	156	36	0	0	1915	526	0	167	4043	749	0	3524
4	Met Adenocarcinoma	2	4953	71357	15069	97145	44851	19578	148	39117	18	81	35	172	0	0	0	0	99	103	0	0	0	0	0	0
		3	9439	178943	22691	223919	22480	10343	0	16598	0	0	0	0	0	0	0	0	192	25	146	0	0	0	0	0
8	Rectal Ca	2	0	0	0	0	620	0	0	390	0	2	0	0	166	0	0	86	0	112	0	0	281	0	0	216
		3	50	0	0	0	0	0	0	0	170	0	0	0	0	0	0	0	72	0	0	0	0	0	0	0
10	CRC	2	81	0	0	0	0	703	8	0	0	438	0	0	69	656	132	2563	0	0	0	0	1446	1075	0	13882
11	CRC	2	0	0	0	0	0	0	14	0	0	484	44	241	343	0	42	0	0	0	0	0	0	0	0	0

Absolute number of $CD4^+$ or $CD8^+$ T cells producing cytokine or positive for CD107a per 1×10^6 cells plated at start of in vitro stimulation. Numbers in gray are those positive after versus before vaccination. PMBCs were assayed pre and post 1, 2, and 3 cycles of vaccination, where available.

The immunogenicity of the TriAd vaccine (ETBX-011, ETBX-051 and ETBX-061) has been assessed in five patients enrolled in the Phase I study following 1 (n=1), 2 (n=5), and 3 (n=3) cycles of vaccination. Antigen-specific immune responses were assessed with a cytometry-based assay. T cells expressing the type I cytokines IFN-γ, IL-2, TNF-α, and/or the degranulation marker CD107a following stimulation with peptide pools encoding CEA, MUC-1, and Brachyury were

measured before and after vaccination, as previously described [21, 22]. Including all time points examined, unpublished studies by the LTIB have found that 5/5 patients (100%) developed T cell responses to at least one of the antigens (MUC-1, CEA, Brachyury) encoded by the vaccine, with both $CD4^{+}$ and $CD8^{+}$ T cell responses noted. The demonstrated immunogenicity of the TriAd vaccine in advanced and diverse cancer patients enrolled in the Phase I study supports the use of this vaccine in combination immunotherapy studies in more homogeneous populations.

1.2.10 Sequential arm administration of neoadjuvant M7824, N-803 and TriAd vaccine before surgery for advanced-stage, non-HPV associated HNSCC

Cumulatively, these data provide the clinical and scientific rationale for the combination of M7824, N-803 and vaccine treatment in the window of opportunity setting before definitive surgical resection in patients with advanced non-HPV associated HNSCC. Studying immunotherapy in newly diagnosed, treatment naïve patients in the neoadjuvant setting allows pathologic, clinical and immune correlative hypotheses to be explored without reducing chance of cure with definitive standard-of-care treatment. Patients with newly diagnosed, advanced HNSCC generally require four to five weeks of treatment planning before definitive treatment begins, providing a treatment window associated with standard of care [23]. Patients will have the opportunity to benefit from neoadjuvant immunotherapy as they await surgical treatment. Since treatment will occur over 2 weeks during this 4-5week window, the treatment schedule will not delay the planned date of definitive surgery. In the window setting, clinical and scientific hypotheses can also be studied quickly given the short duration of neoadjuvant treatment. The multi-arm design of this study with sequential addition of immune-oncology agents allows for assessment of safety and clinical activity in each arm before enrolling subsequent arms.

Our primary outcome measures are both based upon pathologic analysis of surgical resected tissues. The significance of a pathologic CR or clinical-to-pathologic downstaging in HNSCC is unknown. This may be determined from neoadjuvant immunotherapy clinical trials, such as the trial proposed here, that can correlate pCR and clinical-to-pathologic downstaging with 1- and 2-year recurrence free survival. Since all forms of adjuvant treatment for HNSCC cause post-treatment morbidity, identifying a neoadjuvant treatment that may eliminate the need for adjuvant chemotherapy or radiotherapy, in some patients, could limit post-treatment morbidity in patients that have surgical resection. Adjuvant therapy is based upon result from the surgical pathology report, with clear criteria that dictate whether adjuvant chemotherapy or radiotherapy are indicated. Clinical-to pathologic downstaging may eliminate the need for one or more adjuvant treatments.

Pharmacodynamic data from Nant suggest optimal T cell and NK cell is achieved with 15 mcg/kg dosing every 3 weeks. Therefore, a one-time dose of 15 mcg/kg N-803 on day 1 of treatment will be used in this study. M7824 is given at a flat dose of 1,200 mg intravenously. This is the standard phase II dosing of this agent. TriAd vaccine will be given based on dosing from the phase I study of this vaccine platform.

1.2.11 Rationale Summary

- Clinical outcomes for non-HPV-associated HNSCC are poor.
- Generating tumor associated antigen-specific T cells (e.g. CEA, MUC1, and brachyury) and then enabling their activity with combination immuno-oncologic (I/O) agents (e.g.

M7824 and N-803) has been validated preclinically as a strategy for increasing immune-mediated anti-tumor activity

- M7824 and N-803 also work in concert to enhance anti-tumor activity in a non-antigen specific fashion (i.e. enhanced NK cell-mediated antibody dependent cellular cytotoxicity).
- The window between diagnosis and resection of non-HPV associated HNSCC tumors presents a "window" opportunity to test minimally toxic combination immuno-oncologic agents' ability to improve clinical outcomes.
- Patients have the opportunity to benefit from immunotherapy during this period of time when they are normally scheduling and awaiting definitive surgery treatment for their HNSCC.
- Pre-treatment biopsies and post-treatment biopsies or resected tumor samples can provide ample tissue for studies aimed at interrogating the effects of combination immuno-oncologic treatments on tumor microenvironment and correlating those effects with clinical activity.

2 ELIGIBILITY ASSESSMENT AND ENROLLMENT

2.1 ELIGIBILITY CRITERIA

2.1.1 Inclusion Criteria

2.1.1.1 Patients must have histologically or cytologically confirmed, previously untreated intermediate/high risk, p16-negative (if oropharyngeal primary tumor), squamous cell carcinoma of the head and neck (T1-T4, N0-N3, M0 stage II, III or IV).

2.1.1.2 Male or female; Age ≥18 years.

2.1.1.3 ECOG performance status ≤ 1 (see Appendix A).

2.1.1.4 Prothombin time (PT) and partial thromboplastin time (PTT) within normal institutional limits. Patients with prolonged PTT determined to be due to lupus anticoagulant will not be excluded.

2.1.1.5 Patients must have adequate organ and marrow function as defined below:

- Absolute neutrophil count ≥ 1000/mcL
- Platelets ≥ 100,000/mcL
- Hemoglobin ≥ 10.0 g/dL
- Total bilirubin within normal institutional limits; in patients with Gilbert's, ≤ 3.0 mg/dL
- AST (AGOT)/ALT (AGPT) ≤ 3X upper limit of normal.
- Creatinine within 1.5X upper limit of normal institutional limits

2.1.1.6 The effects of M7824, TriAd vaccines, and N-803 on the developing human fetus are unknown. For this reason, men and women of childbearing capacity must agree to use highly effective contraception (hormonal or barrier method of birth control; abstinence) prior to study entry, during the study and maintain such contraception until 2 months following the last dose of any study agent. Should a woman become pregnant or suspect

she is pregnant while she or her partner is participating in this study, she should inform her treating physician immediately.

2.1.1.7 Ability of subject to understand and the willingness to sign a written informed consent document

2.1.1.8 Patients with successfully treated hepatitis C virus (HCV) are eligible if HCV viral load is undetectable.

2.1.2 Exclusion Criteria

2.1.2.1 Patients who are immunocompromised as follows:

- Human immunodeficiency virus (HIV) positive patients not on or not compliant with appropriate anti-retroviral therapy, patients with newly diagnosed (i.e. < 6 months) HIV, patients with an HIV viral load exceeding 400 copies/mL, HIV+ patients with a CD4 count < 150 cells/µL, and HIV+ patients on antiretroviral therapy < 1 month are excluded. HIV-positive patients will also be excluded if the PI determines that there is a clinically significant drug-drug interaction.
- Chronic administration (defined as daily or every other day for continued use >14 days) of systemic corticosteroids or other immune suppressive drugs, within 14 days before treatment on study. Physiologic daily dosing of steroids is allowed. Nasal, or inhaled steroid, topical steroid creams and eye drops for small body areas are allowed.
- Patients who have undergone allogeneic peripheral stem cell transplantation, or solid organ transplantation requiring immunosuppression

2.1.2.2 Pregnant women are excluded from this study because M7824 is an agent with the potential for teratogenic or abortifacient effects. Because there is an unknown but potential risk for adverse events in nursing infants secondary to treatment of the mother with M7824 breastfeeding should be discontinued if the mother is treated with M7824. These potential risks may also apply to other agents used in this study.

2.1.2.3 Patients with active systemic autoimmune disease, except patients with type 1 diabetes mellitus, vitiligo, psoriasis, hypo- or hyperthyroid disease not requiring current

immunosuppression, or with other endocrine disorders on replacement hormones, are not excluded if the condition is well controlled.

2.1.2.4 History of allergic reactions attributed to compounds of similar chemical or biologic composition to study agents to be used in the cohort the subject will be enrolled into.

2.1.2.5 Known allergy to eggs, egg products, aminoglycoside antibiotics (for example, gentamicin or tobramycin). Patients enrolling on the M7824 only arm will be exempt from this exclusion.

2.1.2.6 Patients with a history of bleeding diathesis or recent clinically significant bleeding events considered by the Investigator as high risk for investigational drug treatment are excluded.

2.1.2.7 Any condition which, in the opinion of the investigator, would prevent full participation in this trial (including the long-term follow-up), or would interfere with the evaluation of the trial endpoints.

2.1.2.8 Patients with prior live vaccine, investigational drug, chemotherapy, immunotherapy or any prior radiotherapy (except for palliative bone directed therapy) within the past 28 days prior to enrollment.

2.1.2.9 Uncontrolled intercurrent acute or chronic illness including, but not limited to, ongoing or active infection, symptomatic congestive heart failure (>New York Heart Association Class I), hepatic disease, unstable angina pectoris, serious cardiac arrhythmia, requiring medication, uncontrolled hypertension (SBP>170/ DBP>105) or psychiatric illness/social situations within 12 months that would limit compliance with study requirements.

2.1.2.10 Patients who have undergone major surgery within 4 weeks prior to enrollment. A biopsy will not preclude a patient from starting study.

2.1.2.11 Patients with a history of hepatitis B (HBV) are excluded due to potential risk for viral reactivation and resulting liver injury in persons with latent HBV.

2.1.2.12 Patients with treated or active brain metastases are not eligible because we are enrolling non-metastatic head and neck cancer patients in this trial. Standard of care treatment is different for head and neck cancer patients with and without metastatic disease.

2.1.3 Recruitment Strategies

Both men and women and members of all races and ethnic groups are eligible for this trial. This study will be listed on available websites (www.clinicaltrials.gov, https://ccr.cancer.gov/clinical-trials) and participants will be recruited from the current patient population at NIH and referring academic medical centers and private physicians. This study will be posted on NIH websites and on NIH social media forums. There is no plan to advertise this study at this time.

2.2 SCREENING EVALUATION

2.2.1 Screening activities performed prior to obtaining informed consent

Prior to the subject signing the research consent, subjects may be contacted by several means (e.g., email, mail, in person or telephone) for consideration as a potential protocol candidate, as follows:

- Review of existing medical records, including medical history, physical evaluations, laboratory studies, etc.
- Review of existing MRI, x-ray, or CT images
- Review of existing photographs or videos
- Review of existing pathology specimens/reports from a specimen obtained for diagnostic purposes.

2.2.2 Screening activities performed after a consent for screening has been signed

The following activities will be performed only after the subject has signed the consent for study 01-C-0129 on which screening activities will be performed.

Note: Screening evaluation testing/procedures are conducted under the separate screening protocol, 01-C-0129 (Eligibility Screening and Tissue Procurement for the NIH Intramural Research Program Clinical Protocols).

Screening tests and procedures must be performed within 28 days prior to initiation of study therapy, unless otherwise specified:

- Complete medical history and physical examination (including height, weight, vital signs, and ECOG performance status).
- Clinic-based flexible nasopharyngo-laryngoscopy and/or tracheoscopy to document disease to the extent that it can be evaluated without sedation or general anesthesia. This will include standard bright-light endoscopy and may include video-stroboscopy.
- CT of the neck and chest.
- Clinical laboratory tests*
    - Serum Chemistries:
    - o Acute care panel (Na+, K+, Cl-, total CO2, creatinine, glucose, blood urea nitrogen)
    - o Hepatic panel (AST/GOT, ALT/GPT, total bilirubin, direct bilirubin
    - o Mineral panel (albumin, calcium total, magnesium total, phosphorus)
    - o Hematology: complete blood count (CBC) with differential and platelets
    - o Urinalysis
    - o Serum pregnancy test (β-HCG) for females of childbearing-potential and women < 12 months since the onset of menopause (within 3 days prior to enrollment).
    - o HBV, HCV, HIV testing including viral load for HCV and HIV (within 3 months prior to enrollment). CD4 testing may also be required for HIV+ patients
- Histologic confirmation for p16 negativity* if the primary tumor is oropharyngeal (at any time point prior to enrollment). If there is no available tumor sample or pathology report, a biopsy will be performed to confirm the diagnosis using immunohistochemistry for p16. The E6H4 anti-human P16 antibody clone will be used to stain FFPE tissues using standard deparaffinization, staining and mounting techniques.

* Outside test results are acceptable if performed in designated time frame(s) listed above.

2.3 PARTICIPANT REGISTRATION AND STATUS UPDATE PROCEDURES

Registration and status updates (e.g. when a participant is taken off protocol therapy and when a participant is taken off-study) will take place per CCR SOP ADCR-2, CCR Participant Registration & Status Updates found here.

2.3.1 Treatment Assignment and Randomization/Stratification Procedures

2.3.1.1 Cohorts

Number	Description
1	Previously untreated intermediate/high risk squamous cell carcinoma of the head and neck; T1-T4, N0-N3, M0 stage II, III or IV non-HPV associated squamous cell cancer that is surgically resectable

2.3.1.2 Arms

Letter	Description
A	M7824 (Days 1, 15)
B	M7824 + TriAd vaccine (ETBX-011, ETBX-051 and ETBX-061) (Days 1, 15)
C	M7824 + TriAd vaccine (Days 1, 15) + N-803 (Day 1)

2.3.1.3 Arm Assignment

Cohort 1 will be sequentially assigned to arm A, B or C. Enrollment will begin with arm A. After 12 evaluable patients have accrued to arm A, enrollment to arm B will begin. After 12 evaluable patients have accrued to arm B, enrollment to arm C will begin.

2.4 BASELINE EVALUATION

All subjects are required to complete baseline evaluations within 14 days prior to the first planned dosing of the study drug (any screening evaluation done within this 14-day time period can also serve for the baseline evaluation unless otherwise indicated):

- Medical history and physical exam including weight, ECOG performance status and vital signs
- CT scan of the neck and chest (within 28 days prior to first dosing of study drug).
- Concomitant medications and baseline signs and symptoms evaluation
- EKG
- Serum pregnancy test (β-HCG) for females of childbearing-potential and women < 12 months since the onset of menopause (within 3 days prior to study therapy)
- Serum Chemistries:
  - Acute care panel (Na+, K+, Cl-, total CO2, creatinine, glucose, blood urea nitrogen)
  - Hepatic panel (AST/GOT, ALT/GPT, total bilirubin, direct bilirubin

  - Mineral panel (albumin, calcium total, magnesium total, phosphorus)
- Thyroid tests: TSH, free T3 and T4,
- Lipase, amylase, LDH, GGT
- Hematology: CBC with differential and platelets, PT, PTT
- TBNK phenotyping
- Urinalysis
- CK, Uric Acid, Total Protein, CRP
- HLA class I profile (any time prior to study treatment initiation) and may be deferred if it has previously been performed at the NIH.
- For correlative studies: Blood and tissue sample will be collected. Please refer to section 5.1 for details.
- Biopsies of tumor, normal mucosa and skin: This pre-treatment biopsy is mandatory. Whenever possible, this will be done with local anesthesia in the clinic. When tumor is not safely accessible in the clinic, procedure will be done under general anesthesia in the operating room. If general anesthesia is required to biopsy the tumor, the normal mucosa and skin biopsies will be done under general anesthesia as well. Regional metastasis in the cervical lymph nodes may be sampled via core biopsies guided by bimanual palpation or ultrasound.
- Clinic-based flexible nasopharyngo-laryngoscopy and/or tracheoscopy

3 STUDY IMPLEMENTATION

3.1 STUDY DESIGN

This protocol is a window of opportunity neoadjuvant immunotherapy study. After obtaining pre-treatment biopsies, imaging and blood collection, patients will receive the neoadjuvant immunotherapy at the NIH Clinical Center. Patients will receive investigational immunotherapy on days 1 and 15, during the four to five-week period of time when patients have scheduled and are awaiting definitive surgery. Patients will then be taken off treatment on day 21-28 and post-treatment imaging and blood collection will be performed at the NIH Clinical Center. Patients will then be sent back to their referring providers for their definitive standard of care surgery and adjuvant therapy as indicated based upon pathologic analysis of the surgical specimen. At outside institutions, surgery and adjuvant treatment will be performed and chosen solely by outside providers. NCI investigators will have no role in surgery or adjuvant treatments received at outside institutions. In some cases, standard of care surgery may be offered at the NIH Clinical Center under a separate NIDCD protocol (01-DC-0099). This protocol treats patients with neoadjuvant immunotherapy only; standard of care surgery and any indicated adjuvant therapy is not part of this protocol. Tissue blocks or slides from surgically resected tissues will be obtained from these outside institutions and reviewed by the Laboratory of Pathology to determine primary outcome measures. Secondary clinical outcome measures will be determined by CT imaging and by following patients that are off treatment but receiving standard of care surgery and adjuvant therapy.

Immune correlative analyses may be performed on pre- and post-treatment tissues (tumor, normal mucosa and skin) and bloods. Pre-treatment biopsies will be obtained in the clinic setting (OP5 Otolaryngology clinic) under local anesthesia only whenever possible. When not possible or safe to obtain biopsies in the clinic, biopsies will be obtained under general anesthesia in the operating room at the NIH Clinical Center. Post-treatment biopsies will be obtained at the time of standard

of care surgery if the surgery is being performed at the NIH Clinical Center or at Suburban Hospital. If the surgery will be performed at an outside academic institution other than Suburban Hospital, then biopsies will be performed in the clinic setting under local anesthesia. When not possible or safe to obtain biopsies in the clinic, post-treatment biopsies will not be obtained.

If a patient's condition precludes safe performance of any protocol-indicated procedure, the procedure may be canceled at the discretion of the investigator.

3.1.1 Design Schema

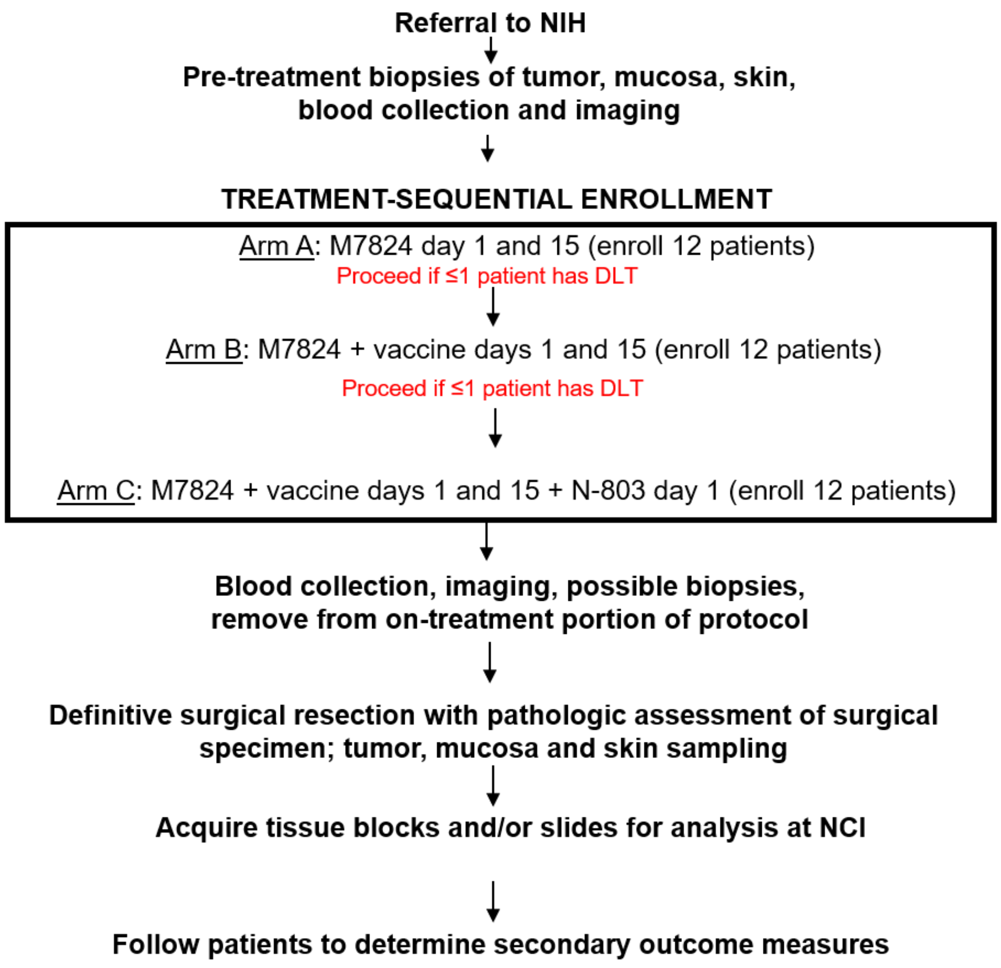

3.1.1.1 Staggered enrollment for Arm B and Arm C

In order to minimize exposure to unanticipated adverse events for the first in human combinations administered to patients in Arm B and Arm C, staggered enrollment will be utilized for the first 3 patients enrolled to Arm B and Arm C.

For Arms B and C:

- An interval of 2 weeks without a DLT must pass between treatment of patient #1 and patient #2.
- An interval of 2 weeks without a DLT must pass between treatment of patient #2 and patient #3.
- An interval of 2 weeks without a DLT must pass between treatment of patient #3 and patient #4.

3.1.2 Dose Limiting Toxicity

Dose-limiting toxicity (DLT) will be defined as any event at least possibly attributable to investigational agents that delays planned tumor resection surgery by >4 weeks or any one of the following adverse events, possibly attributable to study agents (M7824 and/or N-803 and/or TriAd vaccine), that occurs within 28 days after study agent administration.

Any grade ≥ 4 hematologic toxicity or grade ≥ 3 anemia or grade 3 thrombocytopenia (platelets 25-50,000) with associated bleeding, except CD4 lymphocyte count or other T lymphocyte subset count.

- Any grade ≥ 3 non-hematologic toxicity, **except for any of the following**:
    - transient (≤ 48 hour) grade 3 fatigue, local reactions, flu like symptoms, fever, headache, or nausea, emesis, and diarrhea not controlled with adequate medical management, or
    - Any CTCAE Grade 3 skin toxicity lasting less than five days.
    - Electrolyte abnormalities that can be easily managed with replacement therapy. Grade 3 hyperkalemia/hypokalemia that does not resolve to ≤ Grade 2 within 48 hours-72 hours with medical management will be considered a DLT.
    - Asymptomatic Grade 3 lipase or amylase elevation.
    - Grade 3 immune-related colitis or pneumonitis that resolves to grade 1 within 48 hours of initiating appropriate medical treatment
    - Any endocrinopathy that can be medically managed with hormone replacement
- Since patients in this population undergo potentially curative resection, a treatment-related delay in planned surgery >4 weeks past the scheduled date will be considered a DLT. This is consistent with other neoadjuvant studies of checkpoint inhibitors in HNSCC patients.

3.1.3 Study Stopping Rule

Accrual will be halted if there is an occurrence of one grade 5 toxicity or two grade 4 toxicities in a given arm that are at least possibly attributable to the treatment regimen. This does not apply to asymptomatic grade 4 lab abnormalities (e.g. lipase elevation). Prior to resumption of the study, an expedited safety report will be sent to and reviewed by FDA. Serious adverse events (SAEs) also must be evaluated by the clinical investigators.

After completed enrollment of arm A, enrollment to arm B will proceed if ≤1 patient in arm A experience a DLT.

After completed enrollment of arm B, enrollment to arm C will proceed if ≤1 patient in arm B experience a DLT.

If 2 patients in a given study arm experience a DLT, accrual to that arm will be halted.

3.2 DRUG ADMINISTRATION

3.2.1 General rule

A window of +/-3 days for every two weeks study drug(s) dosing is allowed in the event of scheduling issues (i.e. holiday, bad weather or other scheduling issues). A minimum of 10 days must pass between the first M7824/vaccine administration and the second. Any dose that cannot be accommodated within this window will be skipped and the dose not made up.

3.2.2 M7824

Subjects in Arms A, B and C will be scheduled to receive M7824 at a flat dose of 1,200 mg IV on days 1 and 15.

Subjects will receive M7824 via IV infusion over 1 hour (-10 minutes / +20 minutes, that is, over 50 to 80 minutes) once every 2 weeks. M7824 will be administered as a "flat" dose of 1,200 mg independent of body weight. M7824 is administered as an intravenous infusion with a mandatory 0.2 micron in-line filter.

In order to mitigate potential infusion-related reactions, premedication with an antihistamine and with acetaminophen (for example, 25-50 mg diphenhydramine and 500-650 mg acetaminophen) may be given within approximately 30 to 60 minutes prior to dosing of M7824. Steroids as premedication are not permitted. If hypersensitivity reaction occurs, the subject should be treated according to the best available medical practice.

3.2.2.1 Risk for cutaneous keratocanthoma/squamous cell carcinoma with administration of M7824

All cases of skin lesions suspicious for the above will be preferably referred to Dermatology at the NIH. In cases where patients are unable to follow up with Dermatology at the NIH, keratoacanthomas may be managed by an outside dermatologist. Patients will then undergo routine follow-up skin exams and treatment as deemed appropriate by the Dermatologist.

3.2.3 Adenoviral vaccines

ETBX 011, 051 and 061 will be given as subcutaneous injections, on days 1 and 15 of treatment subjects in Arms B and C.

ETBX-011, ETBX-051 and ETBX-061 will be administered by subcutaneous injection in the upper arm or anterolateral upper thigh after preparation of the site with alcohol. A 1 to 1⁄2 inch, 20- to 25- gauge needle should be used for each individual administration. It is preferred to administer ETBX-011, ETBX-051 and ETBX-061 in separate limbs. The limb of administration should be documented in the medical record. When administered in the same thigh, injection sites should be separated by at least 5 cm.

3.2.4 N-803

N-803 will be given via subcutaneous injection to subjects in Arm C at a dose of 15 mcg/kg on day 1.N-803 dosing will be calculated using a weight obtained within 5 days prior to the first dose.

Injection should occur in the abdomen. Depending on the injection volume required, the dose of N-803 may be administered as a single injection or multiple injections at the discretion of the investigator.

Doses of N-803 can be administered on an outpatient basis. Patients should be monitored for 30 minutes post dose. N-803 will not be self-administered.

3.2.4.1 Skin Rash in Association with Subcutaneous Administration of N-803

Based on current experience, localized skin rashes are common with subcutaneous administration. If a rash occurs and the rash area surrounding the N-803 injection site is > 6 cm and symptomatic (painful and/or itchy), it may be treated (at the discretion of the treating physician) with topical 0.05% clobetasol propionate (i.e. 0.05% Cormax) or 0.1% triamcinolone (i.e., Kenalog) cream. Diphenhydramine may be administered pre- (25-50 mg TID orally) and post-dosing (25-50 mg TID orally x 2 days) of N-803 at the discretion of the treating physician. Diphenhydramine should be eliminated if not tolerated. Other agents may be administered at the discretion of the treating physician.

3.2.5 Sequence and Monitoring of Dose Administration

When administering concurrent investigational agents on the same day, the following sequence of administration is preferred. Acetaminophen 650 mg and Benadryl 50 mg by mouth (or another equivalent antihistamine) may be given within 30 to 60 minutes prior to M7824 infusion. Patients will be monitored for symptoms of delayed infusion reaction for 10 minutes. N-803 and vaccine administration is preferred to occur after an interval of at least 10 minutes, but no longer than 5 hours, following M7824 infusion completion.

3.3 DOSE MODIFICATIONS

No dose modifications are allowed.

3.3.1 Immune-related adverse events

Management of irAEs attributed should include consideration of published guidelines [24] and the investigator's discretion.

3.3.2 Discontinuation

Treatment will be discontinued in case of following events if occur within the DLT evaluation period:

- Any Grade 4 adverse drug reactions (ADRs), as defined by CTCAE v5 and assessed as related to any study agent by the Investigator, except for laboratory values that are determined to not be clinically significant or single laboratory valued that resolve to Grade ≤ 1 or baseline grade within 7 days with adequate medical management.
- Any Grade 3 ADRs except for any of the following:
    - Transient flu-like symptoms or fever, which is controlled with medical management.
    - Transient fatigue, local reactions, headache, nausea, emesis which is controlled with medical management.
    - Tumor flare phenomenon defined as local pain, irritation, or rash localized at sites of known or suspected tumor.

- Any drug-related amylase or lipase abnormality that is not associated with symptoms or clinical manifestations of pancreatitis.
- Hgb decrease (< 8.0 g/dL) that is clinically manageable with blood transfusions or erythroid growth factor use does not require treatment discontinuation.
- Keratoacanthoma and squamous cell carcinoma of the skin.
- Any endocrinopathy that can be medically managed with hormone replacement
- Those which in the opinion of the investigator is not clinically relevant or can be medically managed with minimal risk to the patient (e.g. placement of a pleurx catheter for recurrent inflammatory pleural effusions)

3.3.3 Dose hold

In the event that a dose hold of either agent is determined to be necessary by the investigator, the patient will be replaced on study, with the exception of cases of conditions determined to be transient by the principal investigator. In such cases, treatment may be delayed by up to 3 days.

N-803 dosing should be held for hypotension (defined as systolic blood pressure less than 90 mm Hg) if in the presence of any clinically significant symptoms (in the opinion of the treating physician), until the systolic blood pressure reading is stable. If mild dehydration is suspected, an IV fluid bolus may be used per standard of care.

3.4 Study Calendar

Procedure	Screening	Baseline[1]	D1	D8	D15	D21-28	D49	D29-104[14]	D105[15]	Long Term FU[16]
NIH Advance Directive Form[2]		X								
**Evaluations**										
Physical exam, weight and ECOG	X	X			X[18]		X[17]		X	
Radiologic Evaluation[3]	X	X				X				
Histologic confirmation of dx	X									
Medical history	X	X			X[18]		X[17]		X	
Height	X									
EKG		X								
Vital Signs	X	X	X		X[18]		X[17]		X	
**Biopsies**										
Pre-treatment biopsy (mandatory)[5]		X								
Post-treatment biopsy (optional)[6]						X				

Procedure	Screening	Baseline[1]	D1	D8	D15	D21-28	D49	D29-104[14]	D105[15]	Long Term FU[16]
Clinic-based flexible nasopharyngo-laryngoscopy and/or tracheoscopy[19]	X	X			X	X			X	
**Study Agents**										
M7824[7]			X		X					
ETBX 011, 051, and 061[8]			X		X					
N-803[9]			X							
Taken off treatment for definitive surgical resection*						X				
**Laboratory tests**										
CBC w/differential, PT, PTT	X	X			X[18]		X[17]		X	
Chemistry[10]	X	X			X[18]		X[17]		X	
Amylase, lipase, LDH, GGT,		X			X[18]		X[17]		X	
CK, Uric Acid, Total Protein, CRP		X			X[18]		X[17]		X	
Thyroid tests-TSH, free T3 & T4		X			X[18]		X[17]		X	
TBNK phenotyping		X								

**Procedure**	**Screening**	**Baseline**[1]	**D1**	**D8**	**D15**	**D21-28**	**D49**	**D29-104**[14]	**D105**[15]	**Long Term FU**[16]
HIV, Hepatitis B and C serology and/or viral load	X									
Serum pregnancy test[11]	X	X								
HLA Class I [12]		X								
Urinalysis	X	X			X[18]				X	
**Correlative Studies (Please refer to section 5.1 for details)**										
Research blood [13]			X	X	X	X			X	
**Follow-up and others**										
Adverse event evaluation		X			X				X	X
Concomitant meds		X			X				X	X
Phone call or e-mail every two weeks								X		
Phone call or e-mail every 3 months[17]										X

***Resection will be planned and performed under the care of surgeons as per standard of care once patients are taken off of study treatment. The records from outside providers administering standard of care surgery and anti-cancer therapies will be requested in reviewed**. For patients that have surgery outside of the NIH Clinical Center, tissue blocks

and/or slides will be obtained from the institution where the patient underwent surgery after the patient signs that institution's release document.

1. Baseline evaluations do not need to be repeated if performed within given time frame.
2. As indicated in section 12.3, all subjects will be offered the opportunity to complete an NIH Advance Directive form. This should be done preferably at baseline but can be done at any time during the study as long as the capacity to do so is retained. The completion of the form is strongly recommended but is not required.
3. CT of neck and chest. In cases where a CT is not clinically appropriate, other imaging modalities such as MRI may be used as clinically appropriate.
4. Vital signs will be obtained routinely as part of 'physical' exam. Additionally, on D1 and D15 (treatment days) vital signs will be obtained at the beginning of and at the end of M7824 infusion.
5. Mandatory biopsy performed within 5 days prior to day 1 of treatment
6. Optional research biopsy performed if patient not having surgery at NIH Clinical Center or Suburban Hospital. If patient having surgery at NIH or Suburban hospital, tissue samples will be obtained from the OR.
7. 1,200 mg of M7824 via IV infusion on day 1 and day 15. For the prevention of possible allergic reactions, patients may receive an antihistamine such as Benadryl and acetaminophen (Tylenol) before the M7824 infusion. See section
8. Dosing and administration as described in section 3.2.3.
9. N-803 (15mcg/kg) subcutaneous injection on day 1
10. Chemistry: Serum, Acute care panel, Hepatic panel and Mineral panel. Please refer section 2.2 and 2.4 for details.
11. Applies to females of childbearing potential only (within 3 days prior to enrollment)
12. Patients who have had HLA Class I typing previously performed at NIH do not require retesting.
13. For correlative studies: Blood sample will be collected on days 1 and 15 prior giving treatment, Additionally, When no treatment is being given, blood will be collected around day 8 (may occur anytime from days 6-10), within day 21-28, and around day 105. This could occur +/- 3 days for any time point. Please refer to section 5.1 for details. Per PI discretion additional samples might be collected at the NIH Clinical Center every 12 weeks while on study and at the time of taking a patient off treatment.
14. Will occur +/- 3 days for every two-week mark up to day 105.
15. Will occur +/- 2 weeks of day 105.
16. Starting 3 months after the day 105 visit, every 3 months (+/- 1 week), patients will be contacted by phone call or e-mail for questioning regarding disease progression, adverse events, and survival until the patient is 2 years out from surgery. Only AEs considered related to study treatment will be collected as explained in detail under Section 6.1.
17. Visit and labs are to be performed on day 49 (+/- 7 days).
18. Medical history, physical exam and clinical labs may be performed/drawn within 4 days prior to the day 15 treatment

19. . Visualization of tumor with Clinic-based flexible nasopharyngo-laryngoscopy may be deferred in patients whose primary tumors are accessible without these procedures. Nasopharyngo-laryngoscopy done for research purposes may also be deferred in cases where institutional facility procedures are prohibitive (e.g. the appropriate clinical setting is not available due pandemic-related restrictions).

3.5 COST AND COMPENSATION

3.5.1 Costs

NIH does not bill health insurance companies or participants for any research or related clinical care that participants receive at the NIH Clinical Center. If some tests and procedures performed outside the NIH Clinical Center, participants may have to pay for these costs if they are not covered by insurance company. Medicines that are not part of the study treatment will not be provided or paid for by the NIH Clinical Center.

3.5.2 Compensation

There will be no compensation provided in this study.

3.5.3 Reimbursement

On this study, the NCI will cover the cost for some of the expenses. Some of the costs may be paid directly by the NIH and some may be reimbursed to the subject. Someone will work with subjects to provide more information.

3.6 CRITERIA FOR REMOVAL FROM PROTOCOL THERAPY AND OFF STUDY CRITERIA

After completing the study treatment, effort must be made to have all subjects complete the scheduled follow up on day 49.

3.6.1 Criteria for removal from protocol therapy

- Completion of protocol therapy
- Participant requests to be withdrawn from active therapy
- Unacceptable toxicity (see section3.1.2)
- Positive pregnancy test or intent to become pregnant
- Investigator discretion
- Initiation of alternative anticancer therapy including another investigational agent.
- Intercurrent illness that prevents further administration of treatment, in the judgement of the investigator

3.6.2 Off-Study Criteria

- Death
- Completion of 2-year study follow up period
- Subject is lost to follow up*
- Subject requests to be removed from the study
- Investigator discretion
- PI decision to close the study

3.6.3 Lost to Follow-up*

A participant will be considered lost to follow-up if he or she is unable to be reached after reasonable effort is made by the study site staff (for example, 3 telephone calls and, if necessary,

a certified letter to the participant's last known mailing address or local equivalent methods). These contact attempts should be documented in the participant's medical record or study file.

Should the participant continue to be unreachable, he or she will be considered to have withdrawn from the study with a primary reason of lost to follow-up.

4 CONCOMITANT MEDICATIONS/MEASURES

4.1 GENERAL GUIDANCE

Supportive care with blood components, antibiotics, analgesics, general medical therapy, etc., will be delivered as required.

Immunotherapy including interferon, immunosuppressive drugs (for example, chemotherapy or systemic steroids except for short term treatment of allergic reactions, endocrine replacement therapy or low dose prednisone [≤10mg daily] or equivalent, or for the treatment of irAEs or other appropriate short-term steroid use), or other experimental pharmaceutical products are prohibited. Short term administration of systemic steroid or other immunosuppressant such as infliximab or mycophenolate (that is, for allergic reactions or management of irAEs) is allowed. Steroids with no or minimal systemic effect (topical, inhalation), are allowed.

Any live vaccine therapies for the prevention of infectious disease are prohibited. Administration of inactivated vaccines is allowed (for example, inactivated influenza vaccine).

Antiemetics may be used for the treatment of nausea and vomiting but should not be used prophylactically at first dosing. Steroid-based anti-emetics are not allowed.

4.2 N-803

Central nervous system function may be affected with N-803. Caution should be used with psychotropic medications.

Nephrotoxic, myelotoxic, cardiotoxic, or hepatotoxic medications should be avoided if possible as they may further increase toxicities that have been associated with N-803.

Interferon-alfa is prohibited while on study.

Beta-blockers and other antihypertensives may potentiate the hypotension seen with N-803. Therefore, administration of these agents should be avoided during N-803 treatment period, unless clinically indicated. If a patient who is to receive N-803 is on a beta-blocker or other antihypertensive, that agent may be discontinued starting on the day of the first treatment with N-803, if the investigator determines that stopping the agent is safe. The beta blocker may be resumed 2 weeks following the dose of N-803. Anti-hypertensive management decisions will be made on a case by case basis. For example, an anti-hypertensive may be resumed or added at any point if the investigator believes it to be clinically indicated e.g. if a patient experiences uncontrolled hypertension while receiving N-803 treatment on study. For patients on antihypertensives at baseline, the agent will be resumed if clinically indicated by blood pressure readings in the days following N-803 treatment cessation.

If a localized skin rash at the injection site occurs that is >6 cm and symptomatic, it may be treated with 0.05% clobetasol propionate or 0.1% triamcinolone cream or similar product at the discretion of the treating physician.

Administration of additional glucocorticoids is discouraged during the N-803 treatment period as the use of systemic steroid medications may result in loss of therapeutic effects of the study drug. However systemic steroids may be used to treat an irAE.

5 BIOSPECIMEN COLLECTION

5.1 CORRELATIVE STUDIES FOR RESEARCH/PHARMACOKINETIC STUDIES

**Study**	**Test Name**	**Lab Name**	**Sample Time Points**	**Volume/type**	**Tube**
Visualization of immune cells and analysis of immune cell localization 5.1.1	Multiplex immunofluorescence	TIML, GMB, Dr. Houssein Sater	D1 and D21-28 or at surgery	Fixed tumor tissue	N/A
T cell receptor (TCR) sequencing 5.1.2	Deep-TCR sequencing	Frederick Genomic Core Facility, Dr. Xiaolin Wu	D1 and D21-28 or at surgery	Fixed tumor tissue	N/A
Assessment of antigen-specific responses in TIL 5.1.3	RNA-Seq	Sample Preparation in Allen Lab and sequencing at Nant	D1 and D21-28 or at surgery	Fresh tumor tissue	N/A
Exome sequencing, RNA sequencing and neoantigen prediction 5.1.3	DNA and RNA sequencing	Nant	D1 and D21-28 or at surgery	Fixed tumor tissue	N/A

Biomarker analysis related to wound healing 5.1.4	Expression analysis	Allen Lab	D1 and D21-28 or at surgery	Snap frozen tumor, normal mucosa and skin tissue	N/A
Sorting and expansion of B cells to be used as antigen presenting cells for assays 5.1.3	FACS sorting	Allen Lab	D1, 8, 15, 21-28, D105	Whole blood for PBMC	8 (10 mL) sodium heparin green top tubes
Assessment of antigen-specific responses in peripheral T cells. 5.1.5	Intracellular cytokine staining assay	Dr. Duane Hamilton, LTIB, NCI	D1, 8, 15, 21-28, D105	Whole blood for PBMC	
Study of peripheral immune cell accumulation and function by flow cytometry 5.1.6	Flow cytometry	Dr. Renee Donahue, LTIB, NCI	D1, 8, 15, 21-28, D105	Whole blood for PBMC	
Analysis of cytokine concentrations 5.1.7	ELISA or multiplexed assays (e.g. Mesoscale, Luminex, cytokine bead array).	Dr. James Hodge, LTIB, NCI	D1, 8, 15, 21-28, D105	Serum	2 (8 mL) SST tubes

The amount of blood that may be drawn from adult patients for research purposes shall not exceed 10.5 mL/kg or 550 mL, whichever is smaller, over any eight-week period.

Blood samples will be processed and stored through the Clinical Services Program with Leidos Biomedical research, Inc. (CSP) and will be shipped to different labs for research analysis as required. Blood will be collected on days 1 and 15 prior giving treatment, Additionally, when no treatment is being given, blood will be collected on day 8 and within day 21-28, and day 105. This could occur +/- 3 days for any time point.

Tumor, normal mucosa, and skin tissue biopsies will be collected on Day 1 and within Day 21-28 or at surgery. Day 1 tissues will be collected via biopsy in the clinic under local anesthesia for accessible tumors. This pre-treatment biopsy is mandatory for all patients. If tumors are not accessible in the clinic or a biopsy in the clinic is deemed unsafe by the investigator, Day 1 biopsies may be performed in the operating room under general anesthesia. For post-treatment biopsy (on day 21-28 biopsies), if the surgery is performed at the NIH Clinical Center or at Suburban Hospital, the tissue will be acquired from the operating room while the patient is under general anesthesia. If the surgery will be performed at an outside academic institution other than Suburban Hospital, then biopsies will be performed in the clinic setting under local anesthesia at the NIH Clinical Center. This could occur +/- 3 days for any time point and is optional. When not possible or safe to obtain biopsies in the clinic, post-treatment biopsies will not be obtained.

Primary tumor biopsies are limited to 10 per patient per time point; normal mucosa biopsies are limited to 2 per patient per time point; skin biopsies are limited to 1 per patient per time point. All tissue samples required for the Hamilton, Donahue and Hodge labs will be stored in the Allen lab as described later in section 5.2.2 and distributed when they are ready to perform assays.

Tissue and blood samples will be assessed for correlative changes related to development of antigen-specific anti-tumor immunity, general immune activation, and potential alterations in wound healing.

5.1.1 To define immune infiltrate and localization using Multiplex immunofluorescence

Multi-parameter (multi-plex) immunofluorescence using the Perkin-Elmer Opal System will be used to assess tumor samples for infiltration and location of selected immune cell subsets, including effector T cells, regulatory T cells and myeloid cells. This will be done in collaboration with the Tumor Immune Microenvironment Laboratory (TIML) in the Genitourinary Malignancies Branch, NCI managed by Dr. Houssein Sater. Dr. Sater's laboratory is located directly across the hallway from Dr. Allen's laboratory and formalin-fixed parrafin-embedded (FFPE) tissue blocks or cut slides will be delivered by hand from Dr. Allen to Dr. Sater

5.1.2 To determine T cell receptor (TCR) clonality

DeepTCR sequencing will be performed via the Adaptive ImmunoSeq Platform by the Frederick Genomic Core Facility managed by Dr. Xiaolin Wu. This analysis will be performed on FFPE tissue blocks or cut slides. This is a service provided by an NCI Core Facility. Requests for this service will be made online through the NCI at Frederick Access System, which will then provide instructions on shipping and receiving.

5.1.3 Assessment of antigen-specific responses in TIL and antigens predicted from exome and RNA sequencing

Standardized protocols developed in the NCI TIL Laboratory will be used to culture tumor infiltrating lymphocytes (TIL) form tumor fragments. Briefly, tumor will be minced into 1-2 mm fragments and plated in T cell supportive mediate supplemented with human IL-2. Following 3 weeks of culture, T cells will be sorted via negative magnetic selection and assessed for antigen specific responses by ELISpot. Samples stimulated with Ionomycin, phorbol-myristate-acetate (PMA/Iono) will be used as a positive control. Autologous B cells expanded from PBMC will be used as antigen-presenting cells. Messenger RNA (mRNA) encoding genes of interest will be *in vitro* transcribed (IVT) from expression vectors engineered to express genes harboring potential antigens. mRNA will be electroporated into B cells. TIL will be screened for responses against naturally processed and presented MHC class I restricted epitopes from MUC-1, CEA, or brachyury, or predicted neoepitopes. This work will be performed in the Allen Lab, NIDCD. Prediction of neoepitopes requires exome sequencing, RNA sequencing and prediction pipeline bioinformatics. This work will be perfomed by Nant. Nant will use Illumina's HiSeq and NovaSeq platforms for sequencing. All samples will be run according to clinical sequencing quality standards, as regulated by CLIA/CAP, by clinical laboratory scientists with specific certifications as Clinical Genetic Molecular Biologist Scientists Bioinformatic analysis will be performed by both Nant and NCI investigators.

5.1.4 Biomarker analysis related to wound healing.

Expression analysis of genes related to wound dealing and TGFβ signaling may we assessed. Hypotheses related to the role of immunotherapy-induced inflammation, TGFβ signaling and wound healing will be studied by immunohistochemistry, immunofluorescence, and focused PCR arrays on FFPE or frozen tissues. This work will be performed in the Allen Lab, NIDCD.

5.1.5 Assessment of antigen-specific responses in peripheral T cells.

PBMCs may be analyzed for tumor antigen-specific immune responses to MUC-1, CEA, and Brachyury using an intracellular cytokine staining assay. PBMCs will be stimulated *in vitro* with overlapping 15-mer peptide pools encoding the tumor-associated antigens listed above; control peptide pools will involve the use of human leukocyte antigen peptide as a negative control and CEFT peptide mix as a positive control. CEFT is a mixture of peptides of CMV, Epstein-Barr virus, influenza, and tetanus toxin. Post-stimulation analyses of CD4 and CD8 T cells will involve the production of IFN-γ, IL-2, TNF, and the degranulation marker CD107a. If sufficient PBMCs are available, assays may also be performed for the development of T cells to other tumor-associated antigens. A detailed description of this assay has been previously reported [21]. This work will be performed in collaboration with the Laboratory of Dr. Duane Hamilton, LTIB, NCI. When ready to analyze, samples will be requested from Theresa Burks, CSP – Leidos, who will arrange for same day courier delivery of samples on dry ice. These will be received directly br Dr. Duane Hamilton.

5.1.6 Study of peripheral immune cell accumulation and function by flow cytometry

Suspensions of PBMC will be subjected to standard flow cytometry techniques to assess for the accumulation and activation of select immune sell subsets. Antibody panels will test specific

hypotheses related to T cell immune checkpoint expression, as well as Treg and MDSC function and mechanisms of trafficking into tumor. This work will be performed in collaboration with the Laboratory of Dr. Renne Donahue, LTIB, NCI. When ready to analyze, samples will be requested from Theresa Burks, CSP – Leidos, who will arrange for same day courier delivery of samples on dry ice. These will be received directly br Dr. Renae Donahue.

5.1.7 Analysis of cytokine concentrations.

Sera may be analyzed for changes in cytokines (IFN-γ, IL-10, IL-12, IL-2, IL-4, TGFβ, etc.), chemokines, antibodies, tumor-associated antigens, and/or other markers using ELISA or multiplexed assays (e.g. Mesoscale, Luminex, cytokine bead array). This work will be performed in the Laboratory of James Hodge, LTIB, NCI. When ready to analyze, samples will be requested from Theresa Burks, CSP – Leidos, who will arrange for same day courier delivery of samples on dry ice. These will be received directly br Dr. James Hodge

5.2 SAMPLE STORAGE, TRACKING AND DISPOSITION

5.2.1 Frederick Genomic Core Facility, Leidos

Blood samples will be processed and stored through the Clinical Services Program with Leidos Biomedical research, Inc. (CSP).

Clinical Services Program - Leidos Biomedical Research, Inc.

Attn: Theresa Burks

1050 Boyles Street

Bldg. 469/Room 121

Frederick, MD 21702

On days samples are drawn, Jen Bangh at CSP (part of NCI Frederick Central Repositories) should be notified (phone: [301] 846-5893; fax [301] 846-6222). She will arrange same-day courier delivery of the specimens.

- The Clinical Support Laboratory, Leidos Biomedical Research, Inc.-Frederick, processes and cryopreserves samples in support of IRB-approved, NCI clinical trials. All laboratory personnel with access to patient information annually complete the NIH online course in Protection of Human Subjects. The laboratory is CLIA certified for anti-IL15 and certain cytokine measurements, and all laboratory areas operate under a Quality Assurance Plan with documented Standard Operating Procedures that are reviewed annually. Laboratory personnel are assessed for competency prior to being permitted to work with patient samples. Efforts to ensure protection of patient information include:
- The laboratory is located in a controlled access building and laboratory doors are kept locked at all times. Visitors to the laboratory are required to be accompanied by laboratory staff at all times.
- Hard copy records or electronic copies of documents containing patient information are kept in the locked laboratory or other controlled access locations.
- An electronic database is used to store information related to patient samples processed by the laboratory.

- The database resides on a dedicated program server that is kept in a central, locked computer facility.
- The facility is supported by two IT specialists who maintain up to date security features including virus and firewall protection.
- Program access is limited to specified computers as designated by the laboratory director. Each of these computers has a password restricted login screen.
- The database sample entry program itself is accessed through a password protected entry screen.
- The database program has different levels of access approval to limit unauthorized changes to specimen records and the program maintains a sample history.
- Upon specimen receipt each sample is assigned a unique, sequential laboratory accession ID number. All products generated by the laboratory that will be stored either in the laboratory freezers or at a central repository facility are identified by this accession ID.
- Inventory information will be stored at the vial level and each vial will be labeled with both a sample ID and a vial sequence number.
- Vial labels do not contain any personal identifier information.
- Samples are stored inventoried in locked laboratory freezers and are routinely transferred to the NCI-Frederick repository facilities for long term storage.
- Access to stored clinical samples is restricted. Investigators establish sample collections under "Source Codes" and the investigator responsible for the collections, the protocol Principal Investigator, specifies who has access to the collection. Specific permissions will be required to view, input or withdraw samples from a collection.
- Sample withdrawal requests submitted to approved laboratory staff by anyone other than the repository source code owner is submitted to the source code owner for approval. The repository facility will also notify the Source Code holder of any submitted requests for sample withdrawal.
- It is the responsibility of the Source Code holder (the NCI Principal Investigator) to ensure that samples requested and approved for withdrawal are being used in a manner consistent with IRB approval.
- The Clinical Support Laboratory does perform testing services that may be requested by clinical investigators including, but not limited to, immunophenotyping by flow cytometry and cytokine testing using ELISA or multiplex platforms.
- When requests are submitted by the NCI investigator for shipment of samples outside of the NIH it is the policy of the laboratory to request documentation that a Material Transfer Agreement is in place that covers the specimen transfer. At a minimum, the lab needs confirmation that one has been executed or an exception was granted from an office authorized to make such exceptions, e.g. NCI Technical Transfer Center.
- The laboratory does not provide patient identifier information as part of the transfer process but may, at the discretion of the NCI investigator, group samples from individual patients when that is critical to the testing process.
- The NCI investigator responsible for the sample collection is responsible for ensuring appropriate IRB approvals are in place and that a Material Transfer Agreement has been executed prior to requesting the laboratory to ship samples outside of the NIH.

5.2.2 Translational Tumor Immunology Program Laboratory (TTIPL), NIDCD-Allen Lab

All samples are physically stored in Dr. Allen's laboratory in secure, limited-access facilities with sufficient security, backup, and emergency support capability and monitoring to ensure long-term integrity of the specimens for research. Samples are logged, barcoded and tracked using Freezerworks Summit. This data storage software allows secure tracking of specimens that are completely de-identified, coded linked and tracked with barcodes only. Freezerworks has the capacity to fully audit all individual specimen processes such as location, aliquoting, thawing and shipping. Acquired samples are immediately transported to the NIDCD tissue core where they are processed and barcoded. The patient sticker that was used for transport is immediately destroyed. Unique barcodes generated for each individual sample are linked to patient identification numbers given to laboratory staff by NIDCD research nurses. These unique patient numbers correspond to patient information that is stored in CTDM. Protected patient information in CTDM will be recorded by NIDCD Research Nurses. Aside from the patient label that is immediately destroyed after specimen transport, laboratory staff will not have access to protected patient information.

Investigators are granted view, input, and withdrawal authority only for their specimens. They may not view specimen data or access specimens for which they have not been authorized. Access to specimen storage is confined to repository staff. Visitors to the repositories are escorted by repository staff at all times

Tissue biopsies will be ordered in CRIS and any linked protected patient data will be stored in the Clinical Trial Data Management (CTDM) system. Should a CRIS screen not be available, the CRIS downtime procedures will be followed. All tissue biopsies collected will be coded and stored in the NIDCD Biospecimen Tissue Bank.

Contact information:

Biospecimen Tissue Bank Manager:

Paul Clavijo, PhD

Building 10, Room 7S244

Phone: 301-594-6091

Cell: 240-449-6724

Dr. Clavijo will be notified the week before each sample acquisition at our weekly tissue procurement meeting (Thursdays at 2pm in the NIDCD Conference Room, Building 10, Room 7S233). All pre-treatment biopsies will be performed at the NIH Clinical Center. Post-treatment biopsies will be performed at either the NIH Clinical Center or Johns Hopkins Suburban Hospital. Dr. Friedman will be responsible for obtaining the samples from the OP5 clinic or operating room at the NIH and from Suburban Hospital.

5.2.2.1 Storage, Logging, Access to and Sample Destruction upon Completion

Waste tissues will be stored in the NIDCD tissue bank at the NIH Clinical Center. In addition, limited samples may be collected under this protocol as described above. Following enrollment, subjects will be registered and assigned a code in CTDB under which the limited demographic and clinical information will be stored. Only the code will be used on the specimens to electronically log the specimens using a task-specific bar-code technology software which has full security and audit functions consistent with NIH requirements. Samples and the limited demographic and

clinical information will not contain PII. Data should be entered within 2 weeks of patient enrollment onto the protocol. The access to the code keys will be limited to those designated by the Primary Investigator and will be kept in a secured, locked location.

Samples will be stored in freezers, liquid nitrogen containers or tissue cartridge shelves in the NIDCD Core laboratory, located in building 10 on the NIH campus, secured by locked doors.

Access to samples from a protocol for research purposes will be by permission of the Principal Investigator of that protocol in order to be used (1) for research purposes associated with protocol objectives for which the samples were collected, or (2) for a new research activity following submission and IRB approval of a new protocol and consent, or (3) for use only as unlinked or linked samples under the Office of Human Subjects Research Protections (OHSRP) Exemption Form guidelines stipulating that the activity is exempt from IRB review. The use of all samples will be fully tracked and audit lists of when each sample was used and who used it can be generated upon need or request.

Samples, and associated data, will be stored permanently unless the patient withdraws consent. If researchers have samples remaining once they have completed all studies associated with the protocol and patient consent has not been withdrawn, samples may be kept permanently for possible use in future IRB approved investigations.

Once research objectives for the protocol are achieved, researchers can request access to remaining samples, providing they have both approval of the Principal Investigator of the original protocol under which the samples or data were collected and either an IRB approved protocol and patient consent or the OHSRP Exemption Form stipulating that the activity is exempt from IRB review.

5.2.3 Protocol Completion/Sample Destruction

All specimens obtained in the protocol are used as defined in the protocol. Any specimens that are remaining at the completion of the protocol will be stored in the conditions described above. Samples from consenting subjects will be stored until they are no longer of scientific value or if a subject withdraws consent for their continued use, at which time they will be destroyed. If the patient withdraws consent, the participant’s data and specimens will be excluded from future distributions, but data and specimens that have already been distributed for approved research use will not be able to be retrieved.

The PI will record any loss or unanticipated destruction of samples as a deviation. Reports will be made per the requirements of section 7.2.

5.3 SAMPLES FOR GENETIC/GENOMIC ANALYSIS

5.3.1 Description of the scope of genetic/genomic analysis

Using blood and tissue samples, we will perform next generation sequencing (NGS) of DNA and RNA. This will be performed by Nant. Nant will use Illumina's HiSeq and NovaSeq platforms for sequencing.

The primary purpose of this sequencing is to perform bioinformatic prediction of neoepitopes. However, clinically actionable gene variants may be discovered as a result of exome sequencing. Refer to section 5.1.3 .

5.3.2 Description of how privacy and confidentiality of medical information/biological specimens will be maximized

Initially the samples of each patient will be barcoded. At no time will patient's names be used on the blood and tissue samples. Sometimes, because a group collaboration or journal policy requires it, a subject's genetic data will be deposited in a database such as dbGaP. Although there is controlled access to such a database, such a submission carries theoretical risks of revealing the identity of the subject. This is discussed in the consent.

5.3.3 Management of Results

Subjects will be contacted if a clinically actionable gene variant is discovered. Clinically actionable findings for the purpose of this study are defined as disorders appearing in the American College of Medical Genetics and Genomics recommendations for the return of incidental findings that is current at the time of primary analysis. (A list of current guidelines is maintained on the CCR intranet: https://ccrod.cancer.gov/confluence/display/CCRCRO/Incidental+Findings+Lists). Subjects will be contacted at this time with a request to provide a blood sample to be sent to a CLIA certified laboratory. If the research findings are verified in the CLIA certified lab, the subject will be offered the opportunity to come to NIH (at our expense) to have genetic education and counseling to explain this result. If the subject does not want to come to NIH, a referral to a local genetic healthcare provider will be provided (at their expense).

This is the only time during the course of the study that incidental findings will be returned. No interrogations regarding clinically actionable findings will be made after the primary analysis.

5.3.4 Genetic counseling

The costs of CLIA testing will be paid for by the Principal Investigator. If the health history, family history, or tumor diagnosis suggests that the participant might benefit from genetic testing, we will discuss this with him/her.

6 DATA COLLECTION AND EVALUATION

6.1 DATA COLLECTION

The PI will be responsible for overseeing entry of data into an in-house password protected electronic system (C3D) and ensuring data accuracy, consistency and timeliness. The principal investigator, associate investigators/research nurses and/or a contracted data manager will assist with the data management efforts. All data obtained during the conduct of the protocol will be kept in secure network drives or in approved alternative sites that comply with NIH security standards. Primary and final analyzed data will have identifiers so that research data can be attributed to an individual human subject participant.

All adverse events, including clinically significant abnormal findings on laboratory evaluations, regardless of severity, will be followed until return to baseline or stabilization of event. Document AEs from the first study intervention, study day 1, through 30 days after the subject received the last product administration. After 30 days, only adverse events which are serious and related to the study investigational agent need to be recorded. An abnormal laboratory value will be recorded in the database as an AE **only** if the laboratory abnormality is characterized by any of the following:

- Results in discontinuation from the study
- Is associated with clinical signs or symptoms
- Requires treatment or any other therapeutic intervention
- Is associated with death or another serious adverse event, including hospitalization.
- Is judged by the Investigator to be of significant clinical impact
- If any abnormal laboratory result is considered clinically significant, the investigator will provide details about the action taken with respect to the test drug and about the patient's outcome.

End of study procedures: Data will be stored according to HHS, FDA regulations, and NIH Intramural Records Retention Schedule as applicable.

Loss or destruction of data: Should we become aware that a major breach in our plan to protect subject confidentiality and trial data has occurred, this will be reported expeditiously per requirements in section 7.2.1.

6.2 DATA SHARING PLANS

6.2.1 Human Data Sharing Plan

What data will be shared?

I will share human data generated in this research for future research as follows

- Coded, linked data in an NIH-funded or approved public repository.
- Coded, linked data in BTRIS (automatic for activities in the Clinical Center)
- Identified or coded, linked data with approved outside collaborators under appropriate agreements.

How and where will the data be shared?

- An NIH-funded or approved public repository: clinicaltrials.gov, dbGaP
- BTRIS (automatic for activities in the Clinical Center)
- Approved outside collaborators under appropriate individual agreements.
- Publication and/or public presentations.

When will the data be shared?

- Before publication.
- At the time of publication or shortly thereafter.

6.2.2 Genomic Data Sharing Plan

Unlinked genomic data will be deposited in public genomic databases such as dbGaP in compliance with the NIH Genomic Data Sharing Policy.

6.3 RESPONSE CRITERIA

For the purposes of this study, patients will be followed-up every 3 months +/- 1 week. Patients will be contacted by phone call or e-mail for questioning regarding disease progression, adverse events, and survival every 3 months until the patient is 2 years out from surgery.

Primary outcome response measures will be obtained by comparing pre-treatment clinical staging determined by imaging according to Response Evaluation Criteria in Solid Tumors RECIST) guideline (version 1.1) guidelines to the post-treatment pathologic staging obtained from pathologic analysis of the surgically resected specimen. The screening/baseline imaging will be used to determine the pre-treatment TNM classification and stage. Both primary endpoint criteria are based upon results from pathologic analysis of surgically resected tissues. For patients that have surgery outside of the NIH Clinical Center, tissue blocks and/or slides will be obtained from the institution where the patient underwent surgery after the patient signs that institution's release document. These tissue blocks and/or slides will be reviewed by the NCI Laboratory of Pathology. Outside pathology reports may also be obtained from the corresponding institution after the patient sign's that institution's medical release document. In cases where there is discordance between the pathologic staging as determined by an outside institution and the NCI Laboratory of Pathology, we will use the pathologic staging as determined by the NCI Laboratory of Pathology to determine the primary outcome measures.

Secondary outcome response measures will be obtained by comparing CT or MRI of chest and neck obtained at screening/baseline and D21-28. Response or progression when comparing pre- and post-treatment imaging will be evaluated in this study using the new international criteria proposed by RECISTv1.1 guidelines[25]. Changes in the largest diameter (unidimensional measurement) of the tumor lesions and the shortest diameter in the case of malignant lymph nodes are used in the RECIST criteria.

6.3.1 Disease Parameters

Measurable disease: Measurable lesions are defined as those that can be accurately measured in at least one dimension (longest diameter to be recorded) as:

- By chest x-ray: ≥20 mm;
- By CT scan:
    - Scan slice thickness 5 mm or under: as ≥10 mm
    - Scan slice thickness >5 mm: double the slice thickness
- With calipers on clinical exam: ≥10 mm.

All tumor measurements must be recorded in millimeters (or decimal fractions of centimeters).

Malignant lymph nodes. To be considered pathologically enlarged and measurable, a lymph node must be ≥15 mm in short axis when assessed by CT scan (CT scan slice thickness recommended to be no greater than 5 mm). At baseline and in follow-up, only the short axis will be measured and followed.

Non-measurable disease. All other lesions (or sites of disease), including small lesions (longest diameter <10 mm or pathological lymph nodes with ≥10 to <15 mm short axis), are considered

non-measurable disease. Bone lesions, leptomeningeal disease, ascites, pleural/pericardial effusions, lymphangitis cutis/pulmonitis, inflammatory breast disease, and abdominal masses (not followed by CT or MRI), are considered as non-measurable.

Note: Cystic lesions that meet the criteria for radiographically defined simple cysts should not be considered as malignant lesions (neither measurable nor non-measurable) since they are, by definition, simple cysts.

'Cystic lesions' thought to represent cystic metastases can be considered as measurable lesions, if they meet the definition of measurability described above. However, if non-cystic lesions are present in the same patient, these are preferred for selection as target lesions.

<u>Target lesions.</u> All measurable lesions up to a maximum of 2 lesions per organ and 5 lesions in total, representative of all involved organs, should be identified as **target lesions** and recorded and measured at baseline. Target lesions should be selected on the basis of their size (lesions with the longest diameter), be representative of all involved organs, but in addition should be those that lend themselves to reproducible repeated measurements. It may be the case that, on occasion, the largest lesion does not lend itself to reproducible measurement in which circumstance the next largest lesion which can be measured reproducibly should be selected. A sum of the diameters (longest for non-nodal lesions, short axis for nodal lesions) for all target lesions will be calculated and reported as the baseline sum diameters. If lymph nodes are to be included in the sum, then only the short axis is added into the sum. The baseline sum diameters will be used as reference to further characterize any objective tumor regression in the measurable dimension of the disease.

<u>Non-target lesions</u>. All other lesions (or sites of disease) including any measurable lesions over and above the 5 target lesions should be identified as **non-target lesions** and should also be recorded at baseline. Measurements of these lesions are not required, but the presence, absence, or in rare cases unequivocal progression of each should be noted throughout follow-up.

6.3.2 Methods for Evaluation of Measurable Disease

All measurements should be taken and recorded in metric notation using a ruler or calipers. All baseline evaluations should be performed as closely as possible to the beginning of treatment and never more than 4 weeks before the beginning of the treatment.

The same method of assessment and the same technique should be used to characterize each identified and reported lesion at baseline and during follow-up. Imaging-based evaluation is preferred to evaluation by clinical examination unless the lesion(s) being followed cannot be imaged but are assessable by clinical exam.

<u>Clinical lesions:</u> Clinical lesions will only be considered measurable when they are superficial (e.g., skin nodules and palpable lymph nodes) and ≥10 mm diameter as assessed using calipers (e.g., skin nodules). In the case of skin lesions, documentation by color photography, including a ruler to estimate the size of the lesion, is recommended.

<u>Chest x-ray:</u> Lesions on chest x-ray are acceptable as measurable lesions when they are clearly defined and surrounded by aerated lung. However, CT is preferable.

<u>Conventional CT and MRI:</u> This guideline has defined measurability of lesions on CT scan based on the assumption that CT slice thickness is 5 mm or less. If CT scans have slice thickness greater

than 5 mm, the minimum size for a measurable lesion should be twice the slice thickness. MRI is also acceptable in certain situations (e.g. for body scans).

Use of MRI remains a complex issue. MRI has excellent contrast, spatial, and temporal resolution; however, there are many image acquisition variables involved in MRI, which greatly impact image quality, lesion conspicuity, and measurement. Furthermore, the availability of MRI is variable globally. As with CT, if an MRI is performed, the technical specifications of the scanning sequences used should be optimized for the evaluation of the type and site of disease. Furthermore, as with CT, the modality used at follow-up should be the same as was used at baseline and the lesions should be measured/assessed on the same pulse sequence. It is beyond the scope of the RECIST guidelines to prescribe specific MRI pulse sequence parameters for all scanners, body parts, and diseases. Ideally, the same type of scanner should be used and the image acquisition protocol should be followed as closely as possible to prior scans. Body scans should be performed with breath-hold scanning techniques, if possible.

<u>PET-CT:</u> At present, the low dose or attenuation correction CT portion of a combined PET-CT is not always of optimal diagnostic CT quality for use with RECIST measurements. However, if the site can document that the CT performed as part of a PET-CT is of identical diagnostic quality to a diagnostic CT (with IV and oral contrast), then the CT portion of the PET-CT can be used for RECIST measurements and can be used interchangeably with conventional CT in accurately measuring cancer lesions over time. Note, however, that the PET portion of the CT introduces additional data which may bias an investigator if it is not routinely or serially performed.

<u>Ultrasound:</u> Ultrasound is not useful in assessment of lesion size and should not be used as a method of measurement. Ultrasound examinations cannot be reproduced in their entirety for independent review at a later date and, because they are operator dependent, it cannot be guaranteed that the same technique and measurements will be taken from one assessment to the next. If new lesions are identified by ultrasound in the course of the study, confirmation by CT or MRI is advised. If there is concern about radiation exposure at CT, MRI may be used instead of CT in selected instances.

<u>Endoscopy, Laparoscopy:</u> The utilization of these techniques for objective tumor evaluation is not advised. However, such techniques may be useful to confirm complete pathological response when biopsies are obtained or to determine relapse in trials where recurrence following complete response (CR) or surgical resection is an endpoint.

<u>Tumor markers:</u> Tumor markers alone cannot be used to assess response. If markers are initially above the upper normal limit, they must normalize for a patient to be considered in complete clinical response.

<u>Cytology, Histology:</u> These techniques can be used to differentiate between partial responses (PR) and complete responses (CR) in rare cases (e.g., residual lesions in tumor types, such as germ cell tumors, where known residual benign tumors can remain).

The cytological confirmation of the neoplastic origin of any effusion that appears or worsens during treatment when the measurable tumor has met criteria for response or stable disease is mandatory to differentiate between response or stable disease (an effusion may be a side effect of the treatment) and progressive disease.

FDG-PET: While FDG-PET response assessments need additional study, it is sometimes reasonable to incorporate the use of FDG-PET scanning to complement CT scanning in assessment of progression (particularly possible 'new' disease). New lesions on the basis of FDG-PET imaging can be identified according to the following algorithm:

a. Negative FDG-PET at baseline, with a positive FDG-PET at follow-up is a sign of PD based on a new lesion.
b. No FDG-PET at baseline and a positive FDG-PET at follow-up: If the positive FDG-PET at follow-up corresponds to a new site of disease confirmed by CT, this is PD. If the positive FDG-PET at follow-up is not confirmed as a new site of disease on CT, additional follow-up CT scans are needed to determine if there is truly progression occurring at that site (if so, the date of PD will be the date of the initial abnormal FDG-PET scan). If the positive FDG-PET at follow-up corresponds to a pre-existing site of disease on CT that is not progressing on the basis of the anatomic images, this is not PD.
c. FDG-PET may be used to upgrade a response to a CR in a manner similar to a biopsy in cases where a residual radiographic abnormality is thought to represent fibrosis or scarring. The use of FDG-PET in this circumstance should be prospectively described in the protocol and supported by disease-specific medical literature for the indication. However, it must be acknowledged that both approaches may lead to false positive CR due to limitations of FDG-PET and biopsy resolution/sensitivity.

Note: A ‘positive’ FDG-PET scan lesion means one which is FDG avid with an uptake greater than twice that of the surrounding tissue on the attenuation corrected image.

6.3.3 Response Criteria

6.3.4 Evaluation of Target Lesions

Complete Response (CR): Disappearance of all target lesions. Any pathological lymph nodes (whether target or non-target) must have reduction in short axis to <10 mm.

Partial Response (PR): At least a 30% decrease in the sum of the diameters of target lesions, taking as reference the baseline sum of diameters.

Progressive Disease (PD): At least a 20% increase in the sum of the diameters of target lesions, taking as reference the smallest sum on study (this includes the baseline sum if that is the smallest on study). In addition to the relative increase of 20%, the sum must also demonstrate an absolute increase of at least 5 mm. (Note: the appearance of one or more new lesions is also considered progressions).

Stable Disease (SD): Neither sufficient shrinkage to qualify for PR nor sufficient increase to qualify for PD, taking as reference the smallest sum of diameters while on study.

6.3.5 Evaluation of Non-Target Lesions

Complete Response (CR): Disappearance of all non-target lesions and normalization of tumor marker level. All lymph nodes must be non-pathological in size (<10 mm short axis).

Note: If tumor markers are initially above the upper normal limit, they must normalize for a patient to be considered in complete clinical response.

Non-CR/Non-PD: Persistence of one or more non-target lesion(s) and/or maintenance of tumor marker level above the normal limits.

Progressive Disease (PD): Appearance of one or more new lesions and/or *unequivocal progression* of existing non-target lesions. *Unequivocal progression* should not normally trump target lesion status. It must be representative of overall disease status change, not a single lesion increase.

Although a clear progression of "non-target" lesions only is exceptional, the opinion of the treating physician should prevail in such circumstances, and the progression status should be confirmed at a later time by the review panel (or Principal Investigator).

6.3.6 Evaluation of Best Overall Response

The best overall response is the best response recorded from the start of the treatment until disease progression/recurrence (taking as reference for progressive disease the smallest measurements recorded since the treatment started). The patient's best response assignment will depend on the achievement of both measurement and confirmation criteria.

For Patients with Measurable Disease (i.e., Target Disease)

<table>
<tr><th>Target Lesions</th><th>Non-Target Lesions</th><th>New Lesions</th><th>Overall Response</th><th>Best Overall Response when Confirmation is Required*</th></tr>
<tr><td>CR</td><td>CR</td><td>No</td><td>CR</td><td>≥4 wks. Confirmation**</td></tr>
<tr><td>CR</td><td>Non-CR/Non-PD</td><td>No</td><td>PR</td><td rowspan="3">≥4 wks. Confirmation**</td></tr>
<tr><td>CR</td><td>Not evaluated</td><td>No</td><td>PR</td></tr>
<tr><td>PR</td><td>Non-CR/Non-PD/not evaluated</td><td>No</td><td>PR</td></tr>
<tr><td>SD</td><td>Non-CR/Non-PD/not evaluated</td><td>No</td><td>SD</td><td>Documented at least once ≥4 wks. from baseline**</td></tr>
<tr><td>PD</td><td>Any</td><td>Yes or No</td><td>PD</td><td rowspan="2">no prior SD, PR or CR</td></tr>
<tr><td>Any</td><td>PD***</td><td>Yes or No</td><td>PD</td></tr>
</table>

**Target Lesions**	**Non-Target Lesions**	**New Lesions**	**Overall Response**	**Best Overall Response when Confirmation is Required***
Any	Any	Yes	PD	

*	See RECIST 1.1 manuscript for further details on what is evidence of a new lesion.
**	Only for non-randomized trials with response as primary endpoint.
***	In exceptional circumstances, unequivocal progression in non-target lesions may be accepted as disease progression.
Note:	Patients with a global deterioration of health status requiring discontinuation of treatment without objective evidence of disease progression at that time should be reported as "*symptomatic deterioration.*" Every effort should be made to document the objective progression even after discontinuation of treatment.

For Patients with Non-Measurable Disease (i.e., Non-Target Disease)

**Non-Target Lesions**	**New Lesions**	**Overall Response**
CR	No	CR
Non-CR/non-PD	No	Non-CR/non-PD*
Not all evaluated	No	not evaluated
Unequivocal PD	Yes or No	PD
Any	Yes	PD

* 'Non-CR/non-PD' is preferred over 'stable disease' for non-target disease since SD is increasingly used as an endpoint for assessment of efficacy in some trials so to assign this category when no lesions can be measured is not advised

6.4 TOXICITY CRITERIA

The following adverse event management guidelines are intended to ensure the safety of each patient while on the study. The descriptions and grading scales found in the revised NCI Common Terminology Criteria for Adverse Events (CTCAE) version 5.0 will be utilized for AE reporting. All appropriate treatment areas should have access to a copy of the CTCAE version 5.0. A copy of the CTCAE version 5.0 can be downloaded from the CTEP web site (http://ctep.cancer.gov/protocolDevelopment/electronic_applications/ctc.htm).

7 NIH REPORTING REQUIREMENTS/DATA SAFETY MONITORING PLAN

7.1 DEFINITIONS

Please refer to definitions provided in Policy 801: Reporting Research Events found here.

7.2 OHSRP OFFICE OF COMPLIANCE AND TRAINING / IC SPECIFIC IRB REPORTING

7.2.1 Expedited Reporting

Please refer to the reporting requirements in Policy 801: Reporting Research Events and Policy 802 Non-Compliance Human Subjects Research found here. Note: Only IND Safety Reports that meet the definition of an unanticipated problem will need to be reported per these policies.

7.2.2 IRB Requirements for PI Reporting at Continuing Review

Please refer to the reporting requirements in Policy 801: Reporting Research Events found here.

7.3 NCI CLINICAL DIRECTOR REPORTING

Problems expeditiously reported to the OHSRP/IRB in iRIS will also be reported to the NCI Clinical Director. A separate submission is not necessary as reports in iRIS will be available to the Clinical Director.

In addition to those reports, all deaths that occur within 30 days after receiving a research intervention should be reported via email to the Clinical Director unless they are due to progressive disease.

To report these deaths, please send an email describing the circumstances of the death to Dr. Dahut at NCICCRQA@mail.nih.gov within one business day of learning of the death.

7.4 INSTITUTIONAL BIOSAFETY COMMITTEE (IBC) REPORTING CRITERIA

7.4.1 Serious Adverse Event Reports to IBC

The Principal Investigator (or delegate) will notify IBC of any unexpected fatal or life-threatening experience associated with the use of BN-Brachyury as soon as possible but in no event later than 7 calendar days of initial receipt of the information. Serious adverse events that are unexpected and associated with the use of the BN-Brachyury, but are not fatal or life-threatening, must be reported to the NIH IBC as soon as possible, but not later than 15 calendar days after the investigator's initial receipt of the information. Adverse events may be reported by using the FDA Form 3500a.

7.4.2 Annual Reports to IBC

Within 60 days after the one-year anniversary of the date on which the IBC approved the initial protocol, and after each subsequent anniversary until the trial is completed, the Principal Investigator (or delegate) shall submit the information described below. Alternatively, the IRB continuing review report can be sent to the IBC in lieu of a separate report. Please include the IBC protocol number on the report.

7.4.3 Clinical Trial Information

A brief summary of the status of the trial in progress or completed during the previous year. The summary is required to include the following information:

- the title and purpose of the trial
- clinical site
- the Principal Investigator
- clinical protocol identifiers;
- participant population (such as disease indication and general age group, e.g., adult or pediatric);
- the total number of participants planned for inclusion in the trial; the number entered into the trial to date whose participation in the trial was completed; and the number who dropped out of the trial with a brief description of the reasons
- the status of the trial, e.g., open to accrual of subjects, closed but data collection ongoing, or fully completed,
- if the trial has been completed, a brief description of any study results.

7.4.4 Progress Report and Data Analysis

Information obtained during the previous year's clinical and non-clinical investigations, including:

- a narrative or tabular summary showing the most frequent and most serious adverse experiences by body system
- a summary of all serious adverse events submitted during the past year
- a summary of serious adverse events that were expected or considered to have causes not associated with the use of the gene transfer product such as disease progression or concurrent medications
- if any deaths have occurred, the number of participants who died during participation in the investigation and causes of death
- a brief description of any information obtained that is pertinent to an understanding of the gene transfer product’s actions, including, for example, information about dose-response, information from controlled trials, and information about bioavailability.

7.5 NIH REQUIRED DATA AND SAFETY MONITORING PLAN

7.5.1 Principal Investigator/Research Team

The clinical research team will meet weekly when patients are being actively treated on the trial to discuss each patient. Decisions about opening study arms to enrollment will be made based on the toxicity data from prior patients.

All data will be collected in a timely manner and reviewed by the principal investigator or a lead associate investigator.

Events meeting requirements for expedited reporting as described in section 7.2.1 will be submitted within the appropriate timelines.

The principal investigator will review adverse event and response data on each patient to ensure safety and data accuracy. The principal investigator will personally conduct or supervise the investigation and provide appropriate delegation of responsibilities to other members of the research staff.

7.5.2 Safety Monitoring Committee (SMC)

This protocol will be periodically reviewed by an intramural Safety Monitoring Committee. Initial review will occur as soon as possible after the annual NIH Intramural IRB continuing review date. Subsequently, each protocol will be reviewed as close to annually as the quarterly meeting schedule permits or more frequently as may be required by the SMC based on the risks presented in the study.

For initial and subsequent reviews, protocols will not be reviewed if there is no accrual within the review period.

The SMC review will focus on unexpected protocol-specific safety issues that are identified during the conduct of the clinical trial.

Written outcome letters will be generated in response to the monitoring activities and submitted to the Principal investigator and Clinical Director or Deputy Clinical Director, CCR, NCI.

8 SPONSOR PROTOCOL/SAFETY REPORTING

8.1 DEFINITIONS

8.1.1 Adverse Event

Any untoward medical occurrence in a patient or clinical investigation subject administered a pharmaceutical product and which does not necessarily have a causal relationship with this treatment. An adverse event (AE) can therefore be any unfavorable and unintended sign (including an abnormal laboratory finding), symptom, or disease temporally associated with the use of a medicinal (investigational) product, whether or not related to the medicinal (investigational) product (ICH E6 (R2))

8.1.2 Serious Adverse Event (SAE)

An adverse event or suspected adverse reaction is considered serious if in the view of the investigator or the sponsor, it results in any of the following:

- Death,
- A life-threatening adverse event (see 8.1.3)
- Inpatient hospitalization or prolongation of existing hospitalization

- A hospitalization/admission that is pre-planned (i.e., elective or scheduled surgery arranged prior to the start of the study), a planned hospitalization for pre-existing condition, or a procedure required by the protocol, without a serious deterioration in health, is not considered a serious adverse event.
- A hospitalization/admission that is solely driven by non-medical reasons (e.g., hospitalization for patient convenience) is not considered a serious adverse event.
- Emergency room visits or stays in observation units that do not result in admission to the hospital would not be considered a serious adverse event. The reason for seeking medical care should be evaluated for meeting one of the other serious criteria.

- Persistent or significant incapacity or substantial disruption of the ability to conduct normal life functions
- A congenital anomaly/birth defect.
- Important medical events that may not result in death, be life-threatening, or require hospitalization may be considered a serious adverse drug experience when, based upon appropriate medical judgment, they may jeopardize the patient or subject and may require medical or surgical intervention to prevent one of the outcomes listed in this definition.

8.1.3 Life-threatening

An adverse event or suspected adverse reaction is considered "life-threatening" if, in the view of either the investigator or sponsor, its occurrence places the patient or subject at immediate risk of death. It does not include an adverse event or suspected adverse reaction that, had it occurred in a more severe form, might have caused death. (21CFR312.32)

8.1.4 Severity

The severity of each Adverse Event will be assessed utilizing the CTCAE version 5.0.

8.1.5 Relationship to Study Product

All AEs will have their relationship to study product assessed using the terms: related or not related.

- Related – There is a reasonable possibility that the study product caused the adverse event. Reasonable possibility means that there is evidence to suggest a causal relationship between the study product and the adverse event.

- Not Related – There is not a reasonable possibility that the administration of the study product caused the event.

8.2 ASSESSMENT OF SAFETY EVENTS

AE information collected will include event description, date of onset, assessment of severity and relationship to study product and alternate etiology (if not related to study product), date of resolution of the event, seriousness and outcome. The assessment of severity and relationship to the study product will be done only by those with the training and authority to make a diagnosis and listed on the Form FDA 1572 as the site principal investigator or sub-investigator. AEs

occurring during the collection and reporting period will be documented appropriately regardless of relationship. AEs will be followed through resolution.

SAEs will be:

- Assessed for severity and relationship to study product and alternate etiology (if not related to study product) by a licensed study physician listed on the Form FDA 1572 as the site principal investigator or sub-investigator.
- Recorded on the appropriate SAE report form, the medical record and captured in the clinical database.
- Followed through resolution by a licensed study physician listed on the Form FDA 1572 as the site principal investigator or sub-investigator.

For timeframe of recording adverse events, please refer to section 6.1. All serious adverse events recorded from the time of first investigational product administration must be reported to the sponsor.

8.3 Reporting of Serious Adverse Events

Any AE that meets a protocol-defined serious criteria or meets the definition of Adverse Event of Special Interest that require expedited reporting must be submitted immediately (within 24 hours of awareness) to OSRO Safety using the CCR SAE report form.

All SAE reporting must include the elements described in 8.2.

SAE reports will be submitted to the Center for Cancer Research (CCR) at: OSROSafety@mail.nih.gov and to the CCR PI and study coordinator. CCR SAE report form and instructions can be found at: https://ccrod.cancer.gov/confluence/pages/viewpage.action?pageId=157942842

Following the assessment of the SAE by OSRO, other supporting documentation of the event may be requested by the OSRO Safety and should be provided as soon as possible.

8.4 Safety Reporting Criteria to the Pharmaceutical Collaborators

Reporting will be per the collaborative agreements.

8.5 Reporting Pregnancy

8.5.1 Maternal exposure

If a patient becomes pregnant during the course of the study, the study treatment should be discontinued immediately, and the pregnancy reported to the Sponsor no later than 24 hours of when the Investigator becomes aware of it. The Investigator should notify the Sponsor no later than 24 hours of when the outcome of the Pregnancy become known,

Pregnancy itself is not regarded as an SAE. However, congenital abnormalities or birth defects and spontaneous miscarriages that meet serious criteria (8.1.2) should be reported as SAEs.

The outcome of all pregnancies (spontaneous miscarriage, elective termination, ectopic pregnancy, normal birth, or congenital abnormality) should be followed up and documented.

8.5.2 Paternal exposure

Male patients should refrain from fathering a child or donating sperm during the study and for 2 months after the last dose of any investigational drug on this protocol, whichever occurs later.

Pregnancy of the patient's partner is not considered to be an AE. However, the outcome of all pregnancies (spontaneous miscarriage, elective termination, ectopic pregnancy, normal birth, or congenital abnormality) occurring from the date of the first dose until 2 months after the last dose should, if possible, be followed up and documented.

8.6 REGULATORY REPORTING FOR STUDIES CONDUCTED UNDER CCR-SPONSORED IND

Following notification from the investigator, CCR, the IND sponsor, will report any suspected adverse reaction that is both serious and unexpected. CCR will report an AE as a suspected adverse reaction only if there is evidence to suggest a causal relationship between the study product and the adverse event. CCR will notify FDA and all participating investigators (i.e., all investigators to whom the sponsor is providing drug under its INDs or under any investigator's IND) in an IND safety report of potential serious risks from clinical trials or any other source, as soon as possible, in accordance to 21 CFR Part 312.32.

All serious events will be reported to the FDA at least annually in a summary format.

8.7 SPONSOR PROTOCOL NON-ADHERENCE REPORTING

Protocol non-adherence is defined as any noncompliance with the clinical trial protocol, GCP, or protocol-specific procedural requirements on the part of the participant, the Investigator, or the study site staff inclusive of site personnel performing procedures or providing services in support of the clinical trial.

It is the responsibility of the study Staff to document any protocol non-adherence identified by the Staff or the site Monitor on the OSRO Site Protocol Non-Adherence Log. The protocol-specific, cumulative non-adherence log should be maintained in the site essential documents file and submitted to OSRO via OSROMonitoring@mail.NIH.gov on the **first business day of each month over the duration of the study**. In addition, any non-adherence to the protocol should be documented in the participant's source records and reported to the local IRB per their guidelines. OSRO required protocol non-adherence reporting is consistent with E6(R2) GCP: Integrated Addendum to ICH E6(R1): 4.5 Compliance with Protocol; 5.18.3 (a), and 5.20 Noncompliance; and ICH E3 16.2.2 Protocol deviations.

9 CLINICAL MONITORING

Clinical site monitoring is conducted to ensure that the rights of the participants are protected, that the study is implemented per the approved protocol, Good Clinical Practice and standard operating procedures, and that the quality and integrity of study data and data collection methods are maintained. Monitoring for this study will be performed by NCI CCR Office of Sponsor and Regulatory Oversight (OSRO) Monitoring based on OSRO standards, FDA Guidance E6(R2) Good Clinical Practice: Integrated Addendum to ICH E6(R1) March 2018, and applicable regulatory requirements.

Details of clinical site monitoring will be documented in a Clinical Monitoring Plan (CMP) developed by OSRO. CMPs will be protocol-specific, risk-based and tailored to address human subject protections and integrity of the study data. The intensity and frequency of monitoring will be based on several factors, including study type, phase, risk, complexity, expected enrollment rate, and any unique attributes of the study and the site. OSRO Monitoring visits and related activities will be conducted throughout the life cycle of each protocol, with the first activity being before study start to conduct a Site Assessment Visit (SAV) (as warranted), followed by a Site Initiation Visit (SIV), Interim Monitoring Visit(s) (IMVs), and a study Close-Out Visit (COV).

Some monitoring activities may be performed remotely, while others will take place at the study site(s). Monitoring visit reports will describe visit activities, observations, findings of protocol non-adherence and associated action items or follow-up required for resolution of findings. Monitoring reports will be distributed to the study PI, NCI CCR QA, coordinating center (if applicable) and the OSRO regulatory file.

If protocol non-adherence is identified by the Monitor (i.e., any noncompliance with the clinical trial protocol, GCP, or protocol-specific procedural requirements on the part of the participant, the Investigator, or the site Staff) the Monitor will note the observation, review with site Staff and if unresolved, request that the Staff document the non-adherence on the protocol-specific OSRO Site Protocol Non-Adherence Log (see Section 8.7).

10 STATISTICAL CONSIDERATIONS

10.1 STATISTICAL HYPOTHESIS

10.1.1 Primary Endpoint(s):

The primary endpoint of this study is to determine if by using any of the three proposed treatments: M7824 alone, M7824 plus TriAd vaccine, or M7824 plus TriAd vaccine plus N803, there is a sufficiently high success rate in patients with head and neck cancer. Success is defined as pathologic complete response (pCR) or clinical to pathologic downstaging in each patient.

10.1.2 Secondary Endpoint(s):

- Estimate the rate of grade 3 or 4 immune related adverse events (irAEs)
- Determine the rate of treatment-related adverse events (AE) causing a delay of 4 weeks or more beyond planned surgery
- Estimate the response rate of the primary disease by CT imaging (RECIST)
- Estimate the one- and two-year recurrence free survival
- One- and two-year overall survival

10.2 SAMPLE SIZE DETERMINATION

Based on prior reports for single agents, no pCRs but an overall 30% success rate may be anticipated. Because of uncertainty involving delays in surgery following each treatment arm, the following three arms will be enrolled sequentially to permit safety evaluation before adding the next agent:

- M7824 alone
- M7824 + Vaccine
- M7824 + Vaccine + N-803

The trial will begin by enrolling 12 patients to the M7824 alone arm (arm A). For sample size estimation purposes, with 12 patients, there would be 81% power for an exact binomial test with a one-sided 0.10 significance level to detect the difference between a 20% success rate and a 50% success rate in this arm. If 0-1 of the 12 patients experiences a dose limiting toxicity, accrual will then proceed to the 2nd arm (arm B), and 12 patients will enroll following the same criteria. Finally, if 0-1 of 12 patients experiences a dose limiting toxicity while treated in the second arm (arm B), accrual will proceed to the 3rd arm (arm C), again with 12 patients enrolled.

After completion of the 3 arms of 12 patients apiece, the success rates will be evaluated in each arm. Because there is no knowledge of what the success rate of M7824 alone will be in this setting, a more definitive evaluation will first require these preliminary results to determine appropriate parameters for further investigation. Based upon the findings from these 3 limited size arms, including both the pCR rate and clinical downstaging, an amendment will detail the planned expansion as appropriate for A-C of the arms, using parameters derived from information obtained during the 12 patients enrolled initially in the trial.

It is expected that up to 20 patients may enroll in one year. Thus, with 3 arms of 12 patients apiece, up to 36 evaluable patients may enroll. Accrual is expected to be completed within 2 years. To allow for a small number of inevaluable patients, the accrual ceiling will initially be set at 40 patients.

10.3 POPULATIONS FOR ANALYSIS

Modified intention to treat. Any patients who receive at least one dose of any of the agents will be included in any toxicity or safety data reported.

Patients who receive at least one dose of each of the intended agents on the three arms will be included in the analyses of primary and secondary endpoints.

10.4 STATISTICAL ANALYSIS

10.4.1 General approach

If the arms can have 0-1 patients with dose limiting toxicity, the number of patients on each arm who have a pCR or who have clinical to pathologic downstaging will be determined and reported with a confidence interval.

10.4.2 Analysis of primary endpoints

For each arm, the number of patients who experience a pCR will be determined and reported. In addition, for each arm, the fraction of patients who experience clinical to pathologic downstaging will be reported. Finally, for each arm, the fraction of patients who experience either a pCR or clinical to pathologic downstaging will be reported. Each of these fractions will be reported along with a 90% one-sided confidence interval.

10.4.3 Analysis of secondary endpoints

The secondary endpoints and their intended analyses are as follows:

- To estimate the rate of grade 3 or 4 immune-related adverse events; the actual number of patients experiencing these events will be reported separately by arm.
- To determine the rate of patients whose surgery is delayed 4 weeks or greater beyond planned surgery will be reported as the number out of the patients per arm, separately by arm.
- To estimate the response rate of the primary disease by RECIST, the fraction with CR+PR out of evaluable patients will be reported along with a 95% confidence interval, by arm.
- To estimate the 1 year and 2-year RFS rates. A Kaplan-Meier curve of RFS will be constructed for each arm, with the 1- and 2-year values reported along with 95% confidence intervals.
- To estimate the 1 year and 2-year overall survival. A Kaplan-Meier curve of OS will be constructed for each arm, with the 1- and 2-year values reported along with 95% confidence intervals.

10.4.4 Safety analyses

Data on adverse events for each arm will be obtained and reported: to estimate the rate of grade 3 or 4 immune-related adverse events; the actual number of patients experiencing these events will be reported separately by arm.

10.4.5 Baseline descriptive analyses

Descriptive statistics will be determined and reported for patients enrolled onto any of the three treatment arms, separately by arm.

10.4.6 Planned interim analyses

None.

10.4.7 Subgroup analyses

None.

10.4.8 Tabulation of individual patient data

None.

10.4.9 Analysis of exploratory objectives.

The following endpoints will be evaluated:

- Immune infiltrate by flow cytometry and localization using multiplex immunofluorescence
- T cell clonality using Deep TCRseq
- TIL responses to shared antigens and antigens predicted from exome and RNA sequencing
- Antigen specific responses using peripheral T cell isolation

- Multiparameter characterization of PBMC by flow cytometry
- Changes in serum cytokines
- Changes in TGFβ signaling in tumor, normal mucosa and skin

These evaluations may result in descriptive statistics or possibly statistical evaluations of effects. If any statistical tests are performed, they will be conducted in an exploratory fashion, without any adjustment for multiple testing performed but interpreted in the context of the number of tests undertaken.

11 COLLABORATIVE AGREEMENTS

11.1 COOPERATIVE RESEARCH AND DEVELOPMENT AGREEMENT (CRADA)

A CRADA (02666) is in place with EMD Serono for the supply of M7824.

A CRADA (03058) is in place with NantCell for the supply of N-803.

A CRADA (02997) is in place with NantCell for the supply of ETBX (TriAd) vaccines

12 HUMAN SUBJECTS PROTECTIONS

12.1 RATIONALE FOR SUBJECT SELECTION

Subjects from all racial/ethnic groups and both genders are eligible for this study if they meet the eligibility criteria. Efforts will be made to extend accrual to a representative population, but in this preliminary study, a balance must be struck between patient safety considerations and limitations on the number of individuals exposed to potentially toxic and/or ineffective treatments on one hand and the need to explore gender and ethnic aspects of clinical research on the other hand. If differences in outcome that correlate with ethnic identity are noted, accrual may be expanded, or a follow-up study may be written to investigate those differences more fully.

12.2 PARTICIPATION OF CHILDREN

Because no dosing or adverse event data are currently available on the use of each individual agent in patients <18 years of age, children are excluded from this study.

12.3 PARTICIPATION OF SUBJECTS UNABLE TO GIVE CONSENT

Adults unable to give consent are excluded from enrolling in the protocol. However, re-consent may be necessary and there is a possibility, though unlikely, that subjects could become decisionally impaired. For this reason and because there is a prospect of direct benefit from research participation (section 12.4), all subjects ≥ age 18 will be offered the opportunity to fill in their wishes for research and care, and assign a substitute decision maker on the “NIH Advance Directive for Health Care and Medical Research Participation” form so that another person can make decisions about their medical care in the event that they become incapacitated or cognitively impaired during the course of the study. Note: The PI or AI will contact the NIH Ability to Consent Assessment Team (ACAT) for evaluation as needed for the following: an independent assessment of whether an individual has the capacity to provide consent; assistance in identifying and assessing an appropriate surrogate when indicated; and/or an assessment of the capacity to appoint a surrogate. For those subjects that become incapacitated and do not have pre-determined substitute decision maker, the procedures described in NIH HRPP SOP 14E for appointing a

surrogate decision maker for adult subjects who are (a) decisionally impaired, and (b) who do not have a legal guardian or durable power of attorney, will be followed.

12.4 RISK/BENEFITS ASSESSMENT

Patients will receive evaluation of their disease at the National Cancer Institute's Clinical Center. This protocol may or may not benefit an individual, but the results may help the investigators learn more about the disease and develop new treatments for patients with this disease.

Potential adverse reactions attributable to the administration of the treatments utilized in this trial are discussed in section 14. All care will be taken to minimize side effects, but they can be unpredictable in nature and severity. Patients will be examined and evaluated prior to enrollment. All evaluations to monitor the treatment of patients will be recorded in the patient chart.

The potential benefit to a patient that goes onto study is a reduction in the bulk of their tumor which may or may not have favorable impact on symptoms and/or survival.

Potential risks related to treatment on the window trial include unexpected or expected toxicity that may delay or prevent surgical resection, or cause post-operative complications precluding or affecting the tolerance of standard of care adjuvant therapy.

Potential risks and discomforts will also be minimized to the greatest extent possible by using procedures such as appropriate training of personnel, monitoring, withdrawal of the subject upon evidence of difficulty or adverse event; referral for another treatment, counseling or other necessary follow-up.

We anticipate that a thorough discussion of the study at the time informed consent is obtained will minimize any susceptibility to undue influences and unnecessary risks to research subjects.

Although no compensation is available, any injury will be evaluated and treated in keeping with the benefits or care to which patients are entitled under applicable regulations. In all publications and presentations resulting from this trial, patients' anonymity will be protected to the maximum extent possible. Authorized personnel from the National Cancer Institute (NCI) and Food and Drug Administration (FDA) or other regulatory authorities may have access to research files in order to verify that patients' rights have been safeguarded.

12.4.1 Known Potential Risks

12.4.1.1 Research Blood Collection

Risks of blood draws include pain and bruising in the area where the needle is placed, lightheadedness, and rarely, fainting. When large amounts of blood are collected, low red blood cell count (anemia) can develop.

12.4.1.2 Tumor, normal mucosa and skin biopsies

Risks of tumor biopsy include bleeding, minor discomfort, and infection. Risk of bleeding will be minimized with the use of lidocaine with epinephrine local anesthesia and the conservative use of electrocautery (if in the operating room) or silver nitrate (if in the clinic). Discomfort is not a concern if the patient is under general anesthesia and with be minimized in the clinic with the use of injectable local anesthetic per standard-of-care approaches. Risk of injection with biopsies is minimal, but short course of oral antibiotics after biopsies may be provided at the discretion of the

investigator. Biopsies of tumor and normal mucosa will be performed with sterilized 1-3 mm cupped biopsy forceps. Skin biopsies will be obtained with 2-4 mm punch biopsies. For skin biopsies, edges of wound will be approximated with suture using sterile, standard of care technique. Suture closure is not required for tumor or normal mucosal biopsies.

In addition to the risks detailed in the consent for associated with this protocol, patients will be consented for all tumor, normal mucosa and skin tissue biopsies with a clinical consent form for either operative intervention under general anesthesia or for clinic-based procedures under local anesthesia.

12.4.1.3 Risks of the study agents

Risks include the possible occurrence of any of a range of side effects which are listed in the Consent Document or this protocol document. Frequent monitoring for adverse effects will help to minimize the risks associated with administration of the study agents.

12.4.1.4 Risks due to contrast dye used in CT scans

If contrast dye is used, there is a risk for allergic reaction to the dye. Patients who are allergic to or sensitive to medications, contrast dye, iodine, or shellfish should notify their physician. Patients with kidney failure or other kidney problems should notify their physician.

12.4.1.5 Nasopharyngo-laryngoscopy

This procedure takes only a few minutes, is painless and does not require any sedation. It is a normal part of an ENT exam.

12.4.1.6 Risks due to Radiation

The study will involve radiation from the following sources:

- Up to 3 CT scans (Neck and Chest) per year for disease assessment

Subjects in this study may be exposed to approximately 0.99 rem.

The CT scans 3.3 years of background radiation. Most of the time, this amount of extra radiation is not harmful to subjects. However, being exposed to too much radiation can cause harmful side effects including a new cancer in about 1 out of every 1000 people who get a very large amount of extra radiation.

12.4.1.7 Non-Physical Risks of Genetic Research

12.4.1.7.1 Risk of receiving unwanted information

Anxiety and stress may arise as a result of the anticipation that unwanted information regarding disease related DNA sequencing or disease tendencies, or misattributed paternity. Patients will be clearly informed that the data related to DNA sequencing and genetic analysis is coded, investigational and will not be shared with patients, family members or health care providers.

12.4.1.7.2 Risk related to possibility that information may be released

This includes the risk that data related to genotype, DNA sequencing or risk for disease tendency or trait can be released to members of the public, insurers, employers, or law enforcement agencies. Although there are no plans to release results to the patients, family members or health care providers, this risk will be included in the informed consent document.

12.4.1.7.3 Risk to family or relatives

Family members or relatives may or may not want to be aware of familial tendencies or genetic risks of disease which may cause anxiety about possible future health problems. As previously noted, patients will be notified of any medically significant and actionable incidental findings. Study results will not be shared with patients.

12.4.2 Known Potential Benefits

The potential benefit to a patient that goes onto study is a reduction in the bulk of their tumor which may or may not have favorable impact on symptoms and/or survival.

12.5 Consent Process and Documentation

The informed consent document will be provided to the participant or consent designee(s) (e.g., the parent/guardian if participant is a minor, legally authorized representative [LAR] if participant is an adult unable to consent) for review prior to consenting. A designated study investigator will carefully explain the procedures and tests involved in this study, and the associated risks, discomforts and benefits. In order to minimize potential coercion, as much time as is needed to review the document will be given, including an opportunity to discuss it with friends, family members and/or other advisors, and to ask questions of any designated study investigator. A signed informed consent document will be obtained prior to entry onto the study.

The initial consent process as well as re-consent, when required, may take place in person in a private area (e.g., clinic consult room) or remotely (e.g., via telephone or other NIH approved remote platforms) per discretion of the designated study investigator and with the agreement of the participant/consent designee(s). For the optional biopsy for research in the protocol, the patient will consent at the time of the procedure. If the patient refuses the optional biopsy at that time, the refusal will be documented in the medical record and in the research record.

12.5.1 Telephone re-consent

It is permitted for re-consent to occur via telephone. Telephone re-consent will be obtained and documented per NIH CC, OHSRP/IRBO and CCR policies and procedures.

13 REGULATORY AND OPERATIONAL CONSIDERATIONS

13.1 Study Discontinuation and Closure

This study may be temporarily suspended or prematurely terminated if there is sufficient reasonable cause. Written notification, documenting the reason for study suspension or termination, will be provided by the suspending or terminating party to study participants, investigator, the Investigational New Drug (IND) sponsor and regulatory authorities. If the study is prematurely terminated or suspended, the Principal Investigator (PI) will promptly inform

study participants, the Institutional Review Board (IRB), and sponsor and will provide the reason(s) for the termination or suspension. Study participants will be contacted, as applicable, and be informed of changes to study visit schedule.

Circumstances that may warrant termination or suspension include, but are not limited to:

- Determination of unexpected, significant, or unacceptable risk to participants
- Demonstration of efficacy that would warrant stopping
- Insufficient compliance to protocol requirements
- Data that are not sufficiently complete and/or evaluable
- Determination that the primary endpoint has been met
- Determination of futility

Study may resume once concerns about safety, protocol compliance, and data quality are addressed, and satisfy the sponsor, IRB and/or Food and Drug Administration (FDA).

13.2 QUALITY ASSURANCE AND QUALITY CONTROL

Each clinical site will perform internal quality management of study conduct, data and biological specimen collection, documentation and completion. An individualized quality management plan will be developed to describe a site's quality management.

Quality control (QC) procedures will be implemented beginning with the data entry system and data QC checks that will be run on the database will be generated. Any missing data or data anomalies will be communicated to the site(s) for clarification/resolution.

Following written Standard Operating Procedures (SOPs), the monitors will verify that the clinical trial is conducted and data are generated and biological specimens are collected, documented (recorded), and reported in compliance with the protocol, International Conference on Harmonisation Good Clinical Practice (ICH GCP), and applicable regulatory requirements (e.g., Good Laboratory Practices (GLP), Good Manufacturing Practices (GMP)).

The investigational site will provide direct access to all trial related sites, source data/documents, and reports for the purpose of monitoring and auditing by the sponsor, and inspection by local and regulatory authorities.

13.3 CONFLICT OF INTEREST POLICY

The independence of this study from any actual or perceived influence, such as by the pharmaceutical industry, is critical. Therefore, any actual conflict of interest of persons who have a role in the design, conduct, analysis, publication, or any aspect of this trial will be disclosed and managed. Furthermore, persons who have a perceived conflict of interest will be required to have such conflicts managed in a way that is appropriate to their participation in the design and conduct of this trial. The study leadership in conjunction with the National Cancer Institute has established policies and procedures for all study group members to disclose all conflicts of interest and will establish a mechanism for the management of all reported dualities of interest.

14 PHARMACEUTICAL INFORMATION

14.1 ETBX 011, 051, 061

The combination ETBX-011, ETBX-051, ETBX-061 vaccine (collectively called as TriAd vaccine) regimen refers to the combination of three investigational adenoviral vaccines. All utilize the same second generation E1(-), E2 (-) vector. Subjects will each receive the three vaccines at the same time, at the same doses, through three separate injections administered in different limbs (proximal limb areas) or separate locations.

ETBX-011 is a CEA-targeting vaccine that comprises the Ad5 [E1-, E2b-] vector and a modified CEA (CEA(6D)) gene insert. The investigational product ETBX-061 is a MUC1-targeting vaccine that comprises the Ad5 [E1-, E2b-] vector and a modified MUC1 (MUC1c) gene insert. The investigational product ETBX-051 is a Brachyury-targeting vaccine that comprises the Ad5 [E1-, E2b-] vector and a modified Brachyury gene insert.

14.1.1 Source/Acquisition and Accountability

TriAd vaccine will be provided by NantCell to the investigator. The investigator or designee (e.g. pharmacist) will maintain an ongoing inventory of the investigational product supply according to standard site procedures. The investigational product will be dispensed at the direction of an investigator for administration to a study participant enrolled on the clinical trial. Disposal of expired or unused product will be returned to the manufacturer or disposed of according to standard site procedures based on agreement between the manufacturer and the site.

14.1.2 Dosage Form:

Suspension for injection

**Product Name**	ETBX-011 (Ad5 [E1-, E2b-]-CEA(6D) Vaccine)
**Dose**	5 x 10^{11} VP (standard dose)
**Route of Administration**	SC injection
**Physical Description**	ETBX vaccine is supplied as a sterile, clear solution in a 2-mL single-dose vial. The vaccine is provided at a concentration of 5 x 10^{11} VP per 1 mL and contains ARM formulation buffer (20 mM TRIS, 25 mM NaCl, 2.5% glycerol, pH 8.0). Each vial contains approximately 1.3 mL of the vaccine. ETBX-011 should be stored at ≤ -60°C until ready for use.
**Manufacturer**	Etubics (a wholly owned subsidiary of NantCell, Inc; hereafter referred to as NantCell)
**Product Name(s):**	ETBX-061 (Ad5 [E1-, E2b-]-MUC1 Vaccine)

**Dosage Form:**	Suspension for injection
**Dose**	5 x 10^{11} VP (standard dose)
**Route of Administration**	SC injection
**Physical Description**	ETBX vaccine is supplied as a sterile, clear solution in a 2-mL single-dose vial. The vaccine is provided at a concentration of 5 x 10^{11} VP per 1 mL and contains ARM formulation buffer (20 mM TRIS, 25 mM NaCl, 2.5% glycerol, pH 8.0). Each vial contains1 mL of extractable vaccine. The product should be stored at ≤ -60°C until ready for use.
**Manufacturer**	NantCell
**Product Name(s):**	ETBX-051 (Ad5 [E1-, E2b-]-Brachyury Vaccine)
**Dosage Form:**	Suspension for injection
**Dose**	5 x 10^{11} VP (standard dose),
**Route of Administration**	SC injection
**Physical Description**	ETBX vaccine is supplied as a sterile, clear solution in a 2-mL single-dose vial. The vaccine is provided at a concentration of 5 x 10^{11} VP per 1 mL and contains ARM formulation buffer (20 mM TRIS, 25 mM NaCl, 2.5% glycerol, pH 8.0). Each vial contains 1 mL of extractable vaccine. The product should be stored at ≤ -60°C until ready for use.
**Manufacturer**	NantCell

14.1.3 Source

The manufacturing department of NantCell will supply each vaccine in 2-mL single-dose vials. Each single-dose vial contains a sterile, clear suspension of the ETBX vaccine at 5 x 10^{11} VP per 1 mL intended for single dose administration and contains ARM formulation buffer (20 mM TRIS, 25 mM NaCl, 2.5% glycerol, pH 8.0).

Upon receipt of the investigational product, the Investigator or delegated designee will verify that an appropriate shipping temperature was maintained, conduct an inventory, and sign the drug receipt form and send a scanned copy to the Sponsor contact as designated on the form. If a

temperature excursion occurs, the Sponsor should be notified immediately, and the investigational product must be quarantined and maintained under the correct storage conditions until further instructions are provided by the Sponsor. The temperature excursion form will need to be completed and returned to the Sponsor. The original drug receipt form and packing slip must be retained in the Investigator's pharmacy records.

14.1.4 Toxicity

The safety of immunizations (injections) with the ETBX-011, ETBX-051, ETBX-061 has been established in a phase I clinical trial. Ten subjects enrolled and no DLTs were observed. Common TRAEs were fever and injection site reactions, all grade 1 or 2.

14.1.5 Formulation and Preparation

Each vaccine is supplied as a clear colorless liquid filled in a 2-mL amber vial at a concentration of $5x10^{11}$ total virus particles (VP) per 1.0 mL. Each vial is sealed with a rubber stopper and has a white flip off seal. End user of the product will need to flip the white plastic portion of the cap up/off with their thumb to expose the rubber stopper, and then puncture the stopper with an injection needle to withdraw the liquid. The rubber stopper is secured to the vial with an aluminum crimped seal.

14.1.6 Stability and Storage

Individual vials (in the desired number) of vaccine will be packaged in a cardboard box and will be shipped over dry ice by overnight courier with a temperature monitoring device included. Upon receipt, one will inspect contents of package for any noticeable damages or defects. Unpack the shipment contents and place the cardboard box containing vaccine vials into a freezer with a monitored temperature control. ETBX-011, ETBX-051 and ETBX-061 vials should be stored at ≤ -60°C. Receiver must stop the temperature monitoring device by turning off the power switch (instructions for handling and operation of temperature monitoring device will be provided with the package).

14.1.7 ETBX dose preparation and administration

The dose of ETBX vaccine to be injected is $5x10^{11}$ VP per 1mL. Prior to injection, the appropriate vial should be removed from the freezer and allowed to thaw at controlled room temperature (20–25°C, 68-77°F) for at least 20 minutes and not more than 30 minutes, after which it should be kept at 2-8°C (35–46°F). The vaccine is stable in the vial for at least 8 hours after removal from the freezer when kept refrigerated at 2-8°C (35-46°F). Once the vaccine has been thawed, it must not be refrozen.

Each vial is sealed with a rubber stopper and has a white flip-off seal. The end user of the product will need to flip the white plastic portion of the cap up/off with their thumb to expose the rubber stopper and then puncture the stopper with an injection needle to withdraw the liquid. The rubber stopper is secured to the vial with an aluminum-crimped seal.

The thawed vial should be swirled and then, using aseptic technique, the pharmacist or pharmacist designee should withdraw the appropriate volume from the appropriate vial using an appropriately sized syringe.

The thawed drug product may be stored at 2 – 8°C in the vial or syringe for a total of up to 8 hours prior to delivery to the patient, if necessary. ***Instructions for Dose Preparation – 5 x 10^{11} Virus Particles***

Withdraw 1 mL of contents from the vial, prepare the injection site with alcohol, and administer to the subject by SC injection without any further manipulation (refer to the vial label for a description of the vialed ETBX vaccine concentration).

14.1.8 Administration

Please refer to section 3.2.3.

14.1.9 Other Information

The Ad5 [E1-, E2b-] vector is non-replicating and its genome does not integrate into the human genome. However, since this is a non-replicating recombinant virus, it is recommended that it be handled under Biosafety Level-2 conditions. Any vialed ETBX vaccine material that has been used in the study should be autoclaved or incinerated after use according to institutional policy and according to local, state and federal regulations.

14.1.10 Incompatibilities

None known

14.2 M7824 (BINTRAFUSP ALFA, MSB0011359C)

14.2.1 Source/Acquisition and Accountability

M7824 is manufactured and supplied for the trial by EMD Serono Research and Development Institute. The investigator or designee (e.g. pharmacist) will maintain an ongoing inventory of the investigational product supply according to standard site procedures. The investigational product will be dispensed at the direction of an investigator for administration to a study participant enrolled on the clinical trial. Disposal of expired or unused product will be returned to the manufacturer or disposed of according to standard site procedures based on agreement between the manufacturer and the site.

14.2.2 Toxicity

In a phase 1, open-label 3+3 dose-escalation study of M7824 in 16 patients, 3 patients experienced grade 3 drug-related adverse events including skin infection secondary to grade 2 bullous pemphigoid, lipase increased, and colitis with associated anemia. There was no grade 4 – 5 treatment related adverse events. Please see table below for details:

Treatment-related TEAE Leading to Permanent Treatment Discontinuation by System Organ Class and Preferred Term in ≥ 2 participants in the Pooled Analysis of Dose Expansion Cohorts (Source: Investigator Brochure v6)

Primary System Organ Class Dictionary-Derived Term	EMR200647-001 (N = 539) N (%)	MS200647-0008 (N = 91) N (%)	Total (N = 630) N (%)
Participants with any SAE	321 (59.6)	50 (54.9)	371 (58.9)
Blood and lymphatic system disorders	27 (5.0)	0	27 (4.3)
Anaemia	19 (3.5)	0	19 (3.0)
Endocrine disorders	11 (2.0)	2 (2.2)	13 (2.1)
Adrenal insufficiency	5 (0.9)	2 (2.2)	7 (1.1)
Gastrointestinal disorders	64 (11.9)	13 (14.3)	77 (12.2)
Abdominal pain	5 (0.9)	1 (1.1)	6 (1.0)
Gastrointestinal haemorrhage	7 (1.3)	1 (1.1)	8 (1.3)
Upper gastrointestinal haemorrhage	2 (0.4)	4 (4.4)	6 (1.0)
Vomiting	6 (1.1)	0	6 (1.0)
General disorders and administration site conditions	70 (13.0)	10 (11.0)	80 (12.7)
Disease progression	43 (8.0)	6 (6.6)	49 (7.8)
General physical health deterioration	6 (1.1)	0	6 (1.0)
Pyrexia	8 (1.5)	1 (1.1)	9 (1.4)
Hepatobiliary disorders	10 (1.9)	8 (8.8)	18 (2.9)
Cholangitis	2 (0.4)	5 (5.5)	7 (1.1)
Infections and infestations	54 (10.0)	9 (9.9)	63 (10.0)
Pneumonia	14 (2.6)	3 (3.3)	17 (2.7)
Sepsis	8 (1.5)	0	8 (1.3)
Metabolism and nutrition disorders	25 (4.6)	3 (3.3)	28 (4.4)
Decreased appetite	5 (0.9)	3 (3.3)	8 (1.3)
Neoplasms benign, malignant and unspecified (incl cysts and polyps)	61 (11.3)	8 (8.8)	69 (11.0)
Keratoacanthoma	9 (1.7)	0	9 (1.4)
Squamous cell carcinoma of skin	22 (4.1)	1 (1.1)	23 (3.7)
Tumour haemorrhage	8 (1.5)	2 (2.2)	10 (1.6)
Renal and urinary disorders	14 (2.6)	0	14 (2.2)
Acute kidney injury	10 (1.9)	0	10 (1.6)
Respiratory, thoracic and mediastinal disorders	68 (12.6)	6 (6.6)	74 (11.7)
Dyspnoea	26 (4.8)	0	26 (4.1)
Pneumothorax	6 (1.1)	1 (1.1)	7 (1.1)
Pulmonary embolism	9 (1.7)	0	9 (1.4)

As of the data cutoff of 24 August 2018, 7 deaths (1.1%) were reported as due to treatment-related TEAE, however an additional death (primary cause of intra-abdominal hemorrhage, assessed as treatment-related) started more than 30 days after the end of treatment and is therefore is not included in above count of treatment-related death. In Study EMR200647-001, 3 participants had a treatment-related death: 1 participant had dyspnea, hemolysis and thrombocytopenia, 1 participant had an intracranial tumor hemorrhage and 1 participant had pneumonia. In Study MS200647-0008, 4 participants had a treatment-related death: 2 participants died due to ILD, 1 participant due to sudden death and 1 participant due to septic shock. Please refer to IB v 6.0 Section 5.2.2.9 for a summary of the SAEs from the Sponsor's Global Drug Safety database from 25 August 2018 to 31 December 2019 for ongoing studies.

Important identified risks have been identified as IRRs including hypersensitivity, irAEs and skin lesions with hyperkeratosis, keratoacanthoma, cutaneous squamous cell carcinoma possibly due to TGFβ inhibition. The important identified risks with M7824 (bintrafusp alfa) observed to date were overall manageable. Anemia, alterations in wound healing or repair of tissue damage, and

embryofetal toxicity remain as important potential risks. Mucosal bleeding events of mild to moderate severity were observed in participants treated with M7824 in ongoing studies and are a potential risk for M7824. Events may include epistaxis, hemoptysis, gingival bleeding or hematuria amongst others. In general, these reactions resolve without discontinuation of treatment.

In addition, after discussion among NCI investigators on multiple protocols using M7824, multiple bleeding events ranging from low grade gingival bleeding and epistaxis to more serious hemoptysis, GI bleeding and hematuria have been observed. Some of these events can be attributed to bleeding events related to cancer directly and others bleeding events can be attributed to colitis or cystitis which is a known toxicity of anti-PD-L1 agents including M7824. However, there remains the possibility that M7824 may increase the overall risk of bleeding in ways that may not be directly related to direct tumor bleeding or inflammatory bleeding events described with checkpoint inhibitors like M7824. It is hypothesized that this possible increased bleeding risk may be due to TGF beta inhibition which has an effect on angiogenesis; bleeding has also been observed in patients receiving M7824 and may be drug-related (e.g., gum bleeding, nose bleeds, coughing up blood, blood in their urine, or blood in the stool). Accordingly, patients will be notified of the same possible risk in the informed consent document for this study (e.g., gum bleeding, nose bleeds, coughing up blood, blood in their urine, or blood in the stool).

Several procedures have been performed on patients receiving M7824 at the NCI and no bleeding complications have been reported.

**Procedure**	**Number of patients**
Percutaneous cholecystostomy	1
Nephrostomy tube placement	1
Liver biopsy	5
Thoracentesis	1
Biliary stent placement	1
Epidural catheter	2
Tracheostomy	1
Resection of CNS metastasis	1
Endoscopic biopsies under anesthesia<br>(13 biopsies per occurrence)	10
Complete debridement of papillomas	4

14.2.3 Formulation and Preparation

M7824 is provided as a sterile liquid formulation and packaged at a 10 mg/mL concentration in USP/ Ph Eur type I 50R vials that are filled with drug product solution to allow an extractable volume of 60 mL (600 mg/60 mL). The vials are closed with rubber stoppers in serum format complying with USP and Ph Eur with an aluminum crimp seal closure. Each single-use vial contains 600mg of M7824, formulated as 10mg/mL of active, 6% (w/v) Trehalose, 40 mM NaCl, 5 mM Methionine, 0.05% (w/v) Tween 20, 10 mM LHistidine at pH 5.5.

The liquid formulation is diluted directly with 0.9% sodium chloride solution for injection. The estimated volumes of delivery are anticipated to be no more than 250mL. The verified concentration range in the infusion solution is 0.16 mg/mL to 9.6 mg/mL.

14.2.4 Stability and storage

M7824 must be stored at 2°C to 8°C until use. Product stored at room temperature for extended periods of time might be subject to degradation. M7824 must not be frozen. Rough shaking of the reconstituted solution must be avoided.

The chemical and physical in-use stability for the infusion solution of M7824 in 0.9% sodium chloride for injection has been demonstrated for a total of 72 hours at room temperature; however, from a microbiological point of view, the diluted solution should be used immediately and is not intended to be stored unless dilution has taken place in controlled and validated aseptic conditions. No other drugs should be added to the infusion containers containing M7824.

14.2.5 Administration procedures

See section 3.2.2.

14.2.6 Incompatibilities

None known

14.3 N-803 (ALT-803)

14.3.1 Acquisition and Accountability

N-803 is manufactured and supplied by NantCell to the Investigator. The investigator or designee (e.g. pharmacist) will maintain an ongoing inventory of the investigational product supply according to standard site procedures. The investigational product will be dispensed at the direction of an investigator for administration to a study participant enrolled on the clinical trial. Disposal of expired or unused product will be returned to the manufacturer or disposed of according to standard site procedures based on agreement between the manufacturer and the site.

14.3.2 Toxicity

In patients receiving the 10 mcg/kg subcutaneous injection, the following were reported grade 1 and 2 toxicities: worsening anemia, nausea/vomiting, constipation, fatigue, fever, peripheral IV infiltration, injection site reactions, pain/aches, sinusitis, hypoalbuminemia, hypocalcemia, hypophosphatemia, decreased iron, headaches, dysgeusia, and cough. Grade 3 hypertension and decreased lymphocyte count were also reported.

14.3.3 Formulation and preparation

N-803 is a soluble complex consisting of two protein subunits of a human IL-15 variant associated with high affinity to a dimeric human IL-15 receptor α (IL-15Rα) sushi domain/human IgG1 Fc fusion protein. The IL-15 variant is a 114 aa polypeptide comprising the mature human IL-15 cytokine sequence with an Asn to Asp substitution at position 72 of helix C (N72D). The human IL-15Rα sushi domain/human IgG1 Fc fusion protein comprises the sushi domain of the human IL-15 receptor α subunit (IL-15Rα) (aa 1-65 of the mature human IL-15Rα protein) linked with the human IgG1 CH2-CH3 region containing the Fc domain (232 amino acids). Aside from the N72D substitution, all of the protein sequences are human. Based on the amino acid sequence of the subunits, calculated molecular weight of complex comprising two IL-15N72D polypeptides and a disulfide linked homodimeric IL-15RαSu/IgG1 Fc protein is 92.4 kilodaltons (kDa). Each IL-15N72D polypeptide has a calculated molecular weight of approximately 12.8 kDa and the IL-15RαSu/IgG1 Fc fusion protein has a calculated molecular weight of approximately 33.4 kDa. Both the IL-15N72D and IL-15RαSu/IgG1 Fc proteins are glycosylated resulting in an apparent molecular weight of N-803 as approximately 113 kDa by size exclusion chromatography. The isoelectric point (pI) determined for N-803 ranges from approximately 5.5 to 6.5. Thus, the fusion protein is negatively charged at pH 7. The calculated molar extinction coefficient at A280 for N-803 is 116,540 M-1, or 1.26 OD280 for a 1 mg/mL solution of N-803, or one OD280 is equivalent to 0.79 mg/mL solution of N-803.

The biological drug product, N-803, is formulated in a phosphate buffered saline solution. The drug substance is produced by a recombinant mammalian cell line and is manufactured using protein-free media.

14.3.4 N-803 is supplied in a 2-mL single-dose/single-use vial containing 0.6 mL of N-803 (extractable volume is 0.5 mL) at a concentration of 1 mg/mL or 2 mg/mL. Stability and storage

Study medication is provided in a 2mL vial containing 0.6 mL of ALT 803 at a concentration of 2 mg/mL. Study medication must be maintained at a temperature between 2 °C and 8°C. The duration of time during which the product remains stable at room temperature will be obtained from NantCell.

14.3.5 Administration procedures

N-803 is administered subcutaneously. See section 3.2.4

14.3.6 Incompatibilities

None known

15 REFERENCES

1. Ang KK, Harris J, Wheeler R, Weber R, Rosenthal DI, Nguyen-Tan PF, Westra WH, Chung CH, Jordan RC, Lu C *et al*: **Human papillomavirus and survival of patients with oropharyngeal cancer**. *N Engl J Med* 2010, **363**(1):24-35.
2. Lewis JS, Beadle B, Bishop JA, Chernock RD, Colasacco C, Lacchetti C, Moncur JT, Rocco JW, Schwartz MR, Seethala RR *et al*: **Human Papillomavirus Testing in Head and Neck Carcinomas: Guideline From the College of American Pathologists**. *Arch Pathol Lab Med* 2018, **142**(5):559-597.
3. Cooper JS, Pajak TF, Forastiere AA, Jacobs J, Campbell BH, Saxman SB, Kish JA, Kim HE, Cmelak AJ, Rotman M *et al*: **Postoperative concurrent radiotherapy and chemotherapy for high-risk squamous-cell carcinoma of the head and neck**. *N Engl J Med* 2004, **350**(19):1937-1944.
4. Machtay M, Moughan J, Trotti A, Garden AS, Weber RS, Cooper JS, Forastiere A, Ang KK: **Factors associated with severe late toxicity after concurrent chemoradiation for locally advanced head and neck cancer: an RTOG analysis**. *J Clin Oncol* 2008, **26**(21):3582-3589.
5. Seiwert TY, Burtness B, Mehra R, Weiss J, Berger R, Eder JP, Heath K, McClanahan T, Lunceford J, Gause C *et al*: **Safety and clinical activity of pembrolizumab for treatment of recurrent or metastatic squamous cell carcinoma of the head and neck (KEYNOTE-012): an open-label, multicentre, phase 1b trial**. *Lancet Oncol* 2016, **17**(7):956-965.
6. Cancer Genome Atlas N: **Comprehensive genomic characterization of head and neck squamous cell carcinomas**. *Nature* 2015, **517**(7536):576-582.
7. Mandal R, Senbabaoglu Y, Desrichard A, Havel JJ, Dalin MG, Riaz N, Lee KW, Ganly I, Hakimi AA, Chan TA *et al*: **The head and neck cancer immune landscape and its immunotherapeutic implications**. *JCI Insight* 2016, **1**(17):e89829.
8. Ferris RL, Blumenschein G, Jr., Fayette J, Guigay J, Colevas AD, Licitra L, Harrington K, Kasper S, Vokes EE, Even C *et al*: **Nivolumab for Recurrent Squamous-Cell Carcinoma of the Head and Neck**. *N Engl J Med* 2016, **375**(19):1856-1867.
9. Forde PM, Chaft JE, Smith KN, Anagnostou V, Cottrell TR, Hellmann MD, Zahurak M, Yang SC, Jones DR, Broderick S *et al*: **Neoadjuvant PD-1 Blockade in Resectable Lung Cancer**. *N Engl J Med* 2018, **378**(21):1976-1986.
10. **https://ascopubs.org/doi/abs/10.1200/JCO.2017.35.15_suppl.6012**.
11. **https://oncologypro.esmo.org/Meeting-Resources/ESMO-2017-Congress/An-Open-label-Multicohort-Phase-1-2-Study-in-Patients-With-Virus-Associated-Cancers-CheckMate-358-Safety-and-Efficacy-of-Neoadjuvant-Nivolumab-in-Squamous-Cell-Carcinoma-of-the-Head-and-Neck-SCCHN**.
12. Knudson KM, Hicks KC, Luo X, Chen JQ, Schlom J, Gameiro SR: **M7824, a novel bifunctional anti-PD-L1/TGFbeta Trap fusion protein, promotes anti-tumor efficacy**

as monotherapy and in combination with vaccine. *Oncoimmunology* 2018, **7**(5):e1426519.

13. Fujii R, Jochems C, Tritsch SR, Wong HC, Schlom J, Hodge JW: **An IL-15 superagonist/IL-15Ralpha fusion complex protects and rescues NK cell-cytotoxic function from TGF-beta1-mediated immunosuppression**. *Cancer Immunol Immunother* 2018, **67**(4):675-689.

14. Strauss L, Bergmann C, Szczepanski M, Gooding W, Johnson JT, Whiteside TL: **A unique subset of CD4+CD25highFoxp3+ T cells secreting interleukin-10 and transforming growth factor-beta1 mediates suppression in the tumor microenvironment**. *Clin Cancer Res* 2007, **13**(15 Pt 1):4345-4354.

15. Mariathasan S, Turley SJ, Nickles D, Castiglioni A, Yuen K, Wang Y, Kadel EE, III, Koeppen H, Astarita JL, Cubas R *et al*: **TGFbeta attenuates tumour response to PD-L1 blockade by contributing to exclusion of T cells**. *Nature* 2018, **554**(7693):544-548.

16. Lan Y, Zhang D, Xu C, Hance KW, Marelli B, Qi J, Yu H, Qin G, Sircar A, Hernandez VM *et al*: **Enhanced preclinical antitumor activity of M7824, a bifunctional fusion protein simultaneously targeting PD-L1 and TGF-beta**. *Sci Transl Med* 2018, **10**(424).

17. Strauss J, Heery CR, Schlom J, Madan RA, Cao L, Kang Z, Lamping E, Marte JL, Donahue RN, Grenga I *et al*: **Phase I Trial of M7824 (MSB0011359C), a Bifunctional Fusion Protein Targeting PD-L1 and TGFbeta, in Advanced Solid Tumors**. *Clin Cancer Res* 2018, **24**(6):1287-1295.

18. Kim PS, Kwilas AR, Xu W, Alter S, Jeng EK, Wong HC, Schlom J, Hodge JW: **IL-15 superagonist/IL-15RalphaSushi-Fc fusion complex (IL-15SA/IL-15RalphaSu-Fc; ALT-803) markedly enhances specific subpopulations of NK and memory CD8+ T cells, and mediates potent anti-tumor activity against murine breast and colon carcinomas**. *Oncotarget* 2016, **7**(13):16130-16145.

19. Jochems C, Tritsch SR, Pellom ST, Su Z, Soon-Shiong P, Wong HC, Gulley JL, Schlom J: **Analyses of functions of an anti-PD-L1/TGFbetaR2 bispecific fusion protein (M7824)**. *Oncotarget* 2017, **8**(43):75217-75231.

20. Wrangle JM, Velcheti V, Patel MR, Garrett-Mayer E, Hill EG, Ravenel JG, Miller JS, Farhad M, Anderton K, Lindsey K *et al*: **ALT-803, an IL-15 superagonist, in combination with nivolumab in patients with metastatic non-small cell lung cancer: a non-randomised, open-label, phase 1b trial**. *Lancet Oncol* 2018, **19**(5):694-704.

21. Heery CR, Singh BH, Rauckhorst M, Marte JL, Donahue RN, Grenga I, Rodell TC, Dahut W, Arlen PM, Madan RA *et al*: **Phase I Trial of a Yeast-Based Therapeutic Cancer Vaccine (GI-6301) Targeting the Transcription Factor Brachyury**. *Cancer Immunol Res* 2015, **3**(11):1248-1256.

22. Heery CR, Ibrahim NK, Arlen PM, Mohebtash M, Murray JL, Koenig K, Madan RA, McMahon S, Marte JL, Steinberg SM *et al*: **Docetaxel Alone or in Combination With a Therapeutic Cancer Vaccine (PANVAC) in Patients With Metastatic Breast Cancer: A Randomized Clinical Trial**. *JAMA Oncol* 2015, **1**(8):1087-1095.

23. Murphy CT, Galloway TJ, Handorf EA, Egleston BL, Wang LS, Mehra R, Flieder DB, Ridge JA: **Survival Impact of Increasing Time to Treatment Initiation for Patients With Head and Neck Cancer in the United States**. *J Clin Oncol* 2016, **34**(2):169-178.

24. Brahmer JR, Lacchetti C, Schneider BJ, Atkins MB, Brassil KJ, Caterino JM, Chau I, Ernstoff MS, Gardner JM, Ginex P *et al*: **Management of Immune-Related Adverse Events in Patients Treated With Immune Checkpoint Inhibitor Therapy: American Society of Clinical Oncology Clinical Practice Guideline**. *J Clin Oncol* 2018, **36**(17):1714-1768.

25. Eisenhauer EA, Therasse P, Bogaerts J, Schwartz LH, Sargent D, Ford R, Dancey J, Arbuck S, Gwyther S, Mooney M *et al*: **New response evaluation criteria in solid tumours: revised RECIST guideline (version 1.1)**. *Eur J Cancer* 2009, **45**(2):228-247.

16 APPENDIX A: -PERFORMANCE STATUS CRITERIA

**ECOG Performance Status Scale**		**Karnofsky Performance Scale**	
Grade	Descriptions	Percent	Description
0	Normal activity. Fully active, able to carry on all pre-disease performance without restriction.	100	Normal, no complaints, no evidence of disease.
		90	Able to carry on normal activity; minor signs or symptoms of disease.
1	Symptoms, but ambulatory. Restricted in physically strenuous activity, but ambulatory and able to carry out work of a light or sedentary nature (e.g., light housework, office work).	80	Normal activity with effort; some signs or symptoms of disease.
		70	Cares for self, unable to carry on normal activity or to do active work.
2	In bed <50% of the time. Ambulatory and capable of all self-care, but unable to carry out any work activities. Up and about more than 50% of waking hours.	60	Requires occasional assistance but is able to care for most of his/her needs.
		50	Requires considerable assistance and frequent medical care.
3	In bed >50% of the time. Capable of only limited self-care, confined to bed or chair more than 50% of waking hours.	40	Disabled, requires special care and assistance.
		30	Severely disabled, hospitalization indicated. Death not imminent.
4	100% bedridden. Completely disabled. Cannot carry on any self-care. Totally confined to bed or chair.	20	Very sick, hospitalization indicated. Death not imminent.
		10	Moribund, fatal processes progressing rapidly.
5	Dead.	0	Dead.
